# Prognostic and Clinicopathological Significance of *CCND1*/Cyclin D1 Upregulation in Melanomas: A Systematic Review and Comprehensive Meta-Analysis

**DOI:** 10.3390/cancers13061314

**Published:** 2021-03-15

**Authors:** Lucía González-Ruiz, Miguel Ángel González-Moles, Isabel González-Ruiz, Isabel Ruiz-Ávila, Pablo Ramos-García

**Affiliations:** 1 Dermatology Service, Ciudad Real General University Hospital, 13005 Ciudad Real, Spain; gruizlucia@gmail.com; 2 School of Dentistry, University of Granada, 18010 Granada, Spain; isagonzru@gmail.com (I.G.-R.); pramos@correo.ugr.es (P.R.-G.); 3 Instituto de Investigación Biosanitaria ibs.GRANADA, 18012 Granada, Spain; iruizavila@gmail.com; 4 WHO Collaborating Group for Oral Cancer, 1211 Geneva, Switzerland; 5 Pathology Service, San Cecilio Hospital Complex, 18016 Granada, Spain

**Keywords:** melanoma, cyclin D1, CCND1, systematic review, meta-analysis

## Abstract

**Simple Summary:**

The incidence of cutaneous melanoma is increasing worldwide, currently responsible for 287,723 new cases and 60,712 deaths per year (GLOBOCAN, IARC, WHO). It should be also highlighted that some less frequent subtypes of melanomas—i.e., acral, uveal, and mucous melanoma—are responsible for significant morbidity associated with metastasis, responding typically worse to newer therapies. Therefore, new biomarkers are needed to improve the prognosis in individual patients. In this sense, the present study showed that CCND1/cyclin D1 upregulation is a common molecular oncogenic alteration in melanomas that probably favors the growth and expansion on cutaneous primary melanomas. Furthermore, immunohistochemical cyclin D1 overexpression strongly predicted a higher Breslow thickness, currently considered the most relevant prognostic factor in individual patients with melanomas. Finally, special attention should be paid to the CCND1/cyclin D1 complex in mucosal melanomas, whose upregulation was strikingly altered.

**Abstract:**

Our objective was to evaluate the prognostic and clinicopathological significance of cyclin D1 (CD1) overexpression/CCND1 amplification in melanomas. We searched studies published before September 2019 (PubMed, Embase, Web of Science, Scopus). We evaluated the quality of the studies included (QUIPS tool). The impact of CD1 overexpression/CCND1 amplification on overall survival and relevant clinicopathological characteristic were meta-analyzed. We performed heterogeneity, sensitivity, small-study effects, and subgroup analyses. Forty-one studies and 3451 patients met inclusion criteria. Qualitative evaluation demonstrated that not all studies were performed with the same rigor, finding the greatest risk of bias in the study confounding domain. Quantitative evaluation showed that immunohistochemical CD1 overexpression had a statistical association with Breslow thickness (*p* = 0.007; OR = 2.09,95% CI = 1.23–3.57), significantly higher frequency of CCND1/cyclin D1 abnormalities has been observed in the primary tumor compared to distant metastases (*p* = 0.004), revealed also by immunohistochemical overexpression of the protein (*p* < 0.001; OR = 0.53,95% CI = 0.40–0.71), while the CCND1 gene amplification does not show association (*p* = 0.43); while gene amplification, on the contrary, appeared more frequently in distant metastases (*p* = 0.04; OR = 1.70,95% CI = 1.01–2.85) and not in the primary tumor. In conclusion, CCND1/cyclin D1 upregulation is a common molecular oncogenic alteration in melanomas that probably favors the growth and expansion of the primary tumor. This upregulation is mainly consequence to the overexpression of the cyclin D1 protein, and not to gene amplification.

## 1. Introduction

The incidence of cutaneous melanoma is currently 287,723 new cases, this tumor being responsible for 60,712 deaths per year (GLOBOCAN, IARC, WHO) [1]. These figures are increasing and is expected to continue growing worldwide [2] as a consequence essentially of excessive exposure to sunlight related to leisure, which undoubtedly will increase the health investment dedicated to the diagnosis and treatment of this tumor [3]. It should be also highlighted that some less frequent subtypes of melanomas—i.e., acral, uveal, and mucous melanomas—are responsible for significant morbidity associated with metastasis, responding typically worse to newer therapies [4]. Although the use of advanced therapies based on new drugs approved by the FDA have been applied to treatment since 2011 [5], all of the aforementioned advises the development of extensive research, which provides enough scientific evidence especially on new therapeutic targets, with the aim of developing new drugs specifically aimed toward these new targets that can improve patient prognosis.

Cyclin D1, encoded by the CCND1 gene located on chromosome band 11q13, promotes cell cycle progression during the G1-S phase [6]. Since some years, it has been known that CCND1 can act as a relevant oncogene in some types of tumors, having established in addition to its pro-proliferative activity several others emerging oncogenic functions including increase of cell migration capacity, inhibition of cell differentiation, inhibition of DNA repair, and involvement in mitochondrial metabolism [7]. The canonical CCND1/cyclin D1 general role—i.e., sustaining cell proliferation—has also been also shown in in vitro [8,9,10] and in vivo [11,12,13] recent studies in melanoma. Although no studies have been designed to provide direct evidence of the other emerging functions of cyclin D1 in melanoma, some studies have observed an increase in cell migration after the activation of upstream regulators of cyclin D1 in melanomas [14,15]. Finally, the pro-proliferative activity of cyclin D1 has also been shown in melanomas in population-based studies, verified through the evaluation of the ki-67 proliferative index [16,17,18].

Gene amplification is the main mechanism of CCND1/cyclin D1 upregulation in cancer [19,20,21], although in melanoma, other genetic alterations seem to be also involved in the dysregulation of the cyclin D1 function, including mutations, polymorphisms, chromosomal translocations, and the activation of several oncogenic pathways (MAPK, PI3K, Wnt, NF-κβ) [22]. CCND1/cyclin D1 upregulation has been reported to be associated with reduced survival and lack of response to antitumor treatment in melanoma [22,23,24]. CCND1/cyclin D1 have also been proposed as a candidate therapeutic target, because their transcription depends on several upstream pathways essential for tumor development. Cyclin D1 could be targeted in melanoma in several ways—extensively reviewed in Yadav et al. (2015) and González-Ruiz et al. (2020)—by inhibition of chromosome band 11q13 and/or CCND1; by direct inhibition of cyclin D1; by CDK inhibition, acting upstream in melanoma-related pathways involving cyclin D1; or combining agents that act against cyclin D1 with other antitumor drugs. This latter therapy has been proposed as the most promising option [22], particulary the combination of BRaf inhibitory drugs with cyclin D1-CDK4/6 inhibitory agents (i.e., abemaciclib [LY2835219], palbociclib [PD0332991], or ribociclib [LEE011]), with some ongoing or completed phase II trials and clinical trials (NCT02202200, NCT02159066, NCT01820364) [13,25,26]. CCND1/cyclin D1 upregulation could also have specific importance in melanoma context as in the prediction of clinicopathological (e.g., higher Breslow thickness [27] or clinical stage [28]) and/or prognostic (e.g., poor survival [29]) outcomes. Although published data support an empirical assumption about the direction of the effect, and our hypothesis is that *CCND1*/cyclin D1 could behave as an useful biomarker, some primary-level studies have reported disparities or a lack of statistical significance [30,31].

With this background, the present paper aims to qualitatively and quantitatively evaluate for first time, through a systematic review and comprehensive meta-analysis, the available scientific evidence on the clinicopathological and prognostic implications of CCND1 gene amplification and cyclin D1 immunohistochemical protein overexpression in melanomas.

## 2. Materials and Methods

The present systematic review and meta-analysis complied with PRISMA guidelines [32] and closely followed the criteria of Cochrane Prognosis Methods Group [33], Cochrane Handbook for Systematic Reviews of Interventions [34], and Centre for Reviews and Dissemination (CRD)’s guidance for undertaking reviews in healthcare [35].

### 2.1. Protocol

In order to minimize the risk of bias and improve the transparency, precision, and integrity of our systematic review and meta-analysis, a protocol on its methodology was registered a protocol in PROSPERO international prospective register of systematic reviews (www.crd.york.ac.uk/PROSPERO (accessed on 15 March 2021), registration number CRD42020153664; a copy of the protocol was included in the Supplementary Materials, pp. 60–64) [36]. The protocol adhered to PRISMA-P guidelines in order to ensure a rigorous approach [37].

### 2.2. Search Strategy

We searched PubMed, Embase, Web of Science, and Scopus databases for studies published before the search date (upper limit = September 2019), with no lower date limit. Searches were conducted by combining thesaurus terms used by the databases (e.g., MeSH and EMTREE) with free terms (the syntax adapted to each database can be found in the Supplementary Materials, Table S1, p. 5), and constructed to maximize sensitivity. We also manually screened the reference lists of retrieved studies for additional relevant studies. All references were managed using Mendeley v.1.19.3 (Elsevier. Amsterdam, The Netherlands); duplicate references were eliminated using this software.

### 2.3. Selection Criteria

Study eligibility criteria were applied independently by two authors (LGR and PRG). Any discrepancies were resolved by consensus with the senior author (MAGM).

Inclusion criteria: (1) Original research studies published in English. (2) Evaluation of CCND1 amplification or cyclin D1 overexpression in human cutaneous, uveal or mucosal melanomas. (3) Analysis of the association of CCND1 and/or cyclin D1 upregulation with clinicopathological and/or prognostic variables. Given the lack of international consensus standards to define survival endpoints, we included studies that used the direct designation of the terms overall survival (OS) or disease-free survival (DFS). OS was defined as the time elapsed from date of diagnosis/surgery to date of death by any cause. DFS was defined as the time elapsed from surgery to the detection of locoregional or distant recurrence or to death without recurrence. Other terms defined in the original studies as in the present article or compatible with our definitions (e.g., relapse-free survival) were also accepted. (4) The author names, affiliations, recruitment periods, and setting were examined to determine whether studies were conducted in the same study population. In such cases, we included the most recent study or that which published more complete data.

Exclusion criteria: (1) Retractions, reviews, meta-analyses, case reports, editorials, letters, abstracts of scientific meetings, personal opinions or comments, book chapters, and any study in a language other than English; (2) Studies with no melanoma cases; (3) Reports of in vitro or animal experiments; (4) Evaluations of *CCND1* gene alterations other than gene amplification (e.g., polymorphisms) and studies of chromosome band 11q13 that do not specifically discriminate the *CCND1* gene; (5) No analysis of the relationship of upregulation with clinicopathological or prognostic variables; (6) Insufficient data for the estimation of odds ratios (ORs) for clinicopathological variables and, in studies only reporting time-to-event variables (OS/DFS), the absence of hazards ratios (HRs) with 95% confidence intervals (CIs) or the lack of adequate data for their calculation by survival analysis.

We first screened the titles and abstracts of retrieved articles to select those that appeared to meet the review eligibility criteria. In a second phase, we examined the full texts of the selected articles and removed any that did not fulfill these criteria.

### 2.4. Data Extraction

Two authors (LGR and PRG) independently extracted data from the articles selected for full text study in a standardized fashion using an Excel data collection template (Excel v.2015, Microsoft. Redmond, WA, USA). The extracted data were then reviewed by the senior author (MAGM). Discrepancies were solved by consensus. Data were gathered on: first author, year of publication, country and continent, sample size, alteration under study (*CCND1* amplification and/or cyclin D1 overexpression), type of melanomas, location, recruitment and follow-up periods, methodology, and the upregulation frequency. Furthermore, in immunohistochemical studies, information was also recorded on the cutoff point, anti-cyclin D1 antibody, and intracellular immunopositivity pattern (nuclear/cytoplasmic).

### 2.5. Evaluation of Quality and Risk of Bias

Two authors (LGR and PRG) evaluated the quality of studies and the risk of bias using the Quality in Prognosis Studies (QUIPS) tool of the Cochrane Prognosis Methods Group [38], which explores six main potential bias domains: (1) Study participation, (2) Study attrition, (3) Prognostic factor measurement, (4) Outcome measurement, (5) Study confounding, and (6) Statistical analysis and reporting [39]. The potential risk of bias was evaluated as low, moderate, or high for each domain. Discrepancies were resolved with a senior author (MAGM).

### 2.6. Statistical Analysis

*CCND1* amplification was considered “positive” or “negative” according to the methodology adopted in each study. Cyclin D1 expression was considered “high” or “low” according to the cutoff values applied in each study. We used hazards ratio (HR) with 95% confidence intervals (CIs) to estimate the impact of CCND1/cyclin D1 upregulation on time-to-event variables (OS and DFS). When reported, HRs and 95% CIs were directly extracted from the original articles. If HRs were not explicitly provided by the authors, they were calculated using the methods described in Parmar et al. [40] and Tierney et al. [41]. When HRs were evaluated in both univariable and multivariable models, we used the data from the multivariable model, which reflect a greater adjustment for potentially confounding variables [42]. When data were only depicted in Kaplan–Meier curves or HRs and 95% CIs were only expressed graphically, data were measured and extracted using Engauge Digitizer 4.1 (open-source digitizing software developed by M. Mitchell). In meta-analyses, HRs and 95% CIs were pooled using a random-effect model (REM, Der Simonian and Laird (D-L) method), because logic dictates the presence of a considerable degree of between-study heterogeneity.

Clinicopathological parameters were meta-analyzed using odds ratios (OR) with 95% CIs. As the authors only reported raw data, this information was collected and re-expressed as ORs, which were pooled by Mantel–Haenszel’s method, using a fixed-effect model. A random-effects model using the inverse variance method was not considered a priori, because when data are sparse, heterogeneity is uncommon and the Mantel–Haenszel weighting method has been shown to have better statistical properties [43]. Peto’s method was not considered because many studies presented unbalanced arms.

Finally, meta-analyses were carried out trying to estimate the overall frequency of cyclin D1/CCND1 expression and amplification levels for the different types of melanoma under investigation (i.e., cutaneous [nodular, superficial spreading, lentigo malignant, and acral melanoma], uveal and mucosal). To achieve this, pooled proportions (PP) with their corresponding 95% CIs were estimated. Proportions in individual studies were calculated by extracting raw numerators (number of cases with cyclin D1 high expression or *CCND1* positive amplification) and denominators (total number of melanoma cases). The 95% CIs were constructed based on the score-test statistic [44].The influence of studies with extremely small or high values (0 or 100, or close to 0 or 100) was minimized by using Freeman–Tukey double-arcsine transformation to stabilize the variance of proportions [45]. PP were estimated using a REM (D-L method).

In all conducted meta-analyses, forest plots were constructed to graphically represent the overall effect and for its subsequent analysis (a *p*-value < 0.05 was considered statistically significant) [46]. Heterogeneity between studies was checked using the χ^2^ based Cochran’s Q test. Given the low statistical power of this test, *p* < 0.1 was considered significant. We also used Higgins I^2^ statistic to quantify the percentage heterogeneity, considering values of 25, 50, and 75% to indicate low, moderate, and high heterogeneity, respectively [46,47]. Additionally, subgroup analyses—preplanned in our protocol—were performed (stratifying by CCND1/cyclin D1 upregulation, geographic area, cut-off value, and immunostaining pattern) to identify potential sources of heterogeneity and to explore the relationship between the meta-analyzed parameters in these subgroups. Sensitivity analyses were additionally carried out to test the reliability of combined results, evaluating the influence of each individual study on the final estimations for each meta-analysis performed [48]. For this, the “leave-one-out” method was used (i.e., the meta-analyses were repeated sequentially, omitting one study at a time). Funnel plots were constructed and the Egger regression test (*p*_Egger_ < 0.1) was applied to evaluate small-study effects, such as publication bias [49,50]. Stata version 14.1 (Stata Corporation, College Station, TX, USA) was employed for all tests, using commands written by the user.

## 3. Results

### 3.1. Literature Search

**Figure 1:**
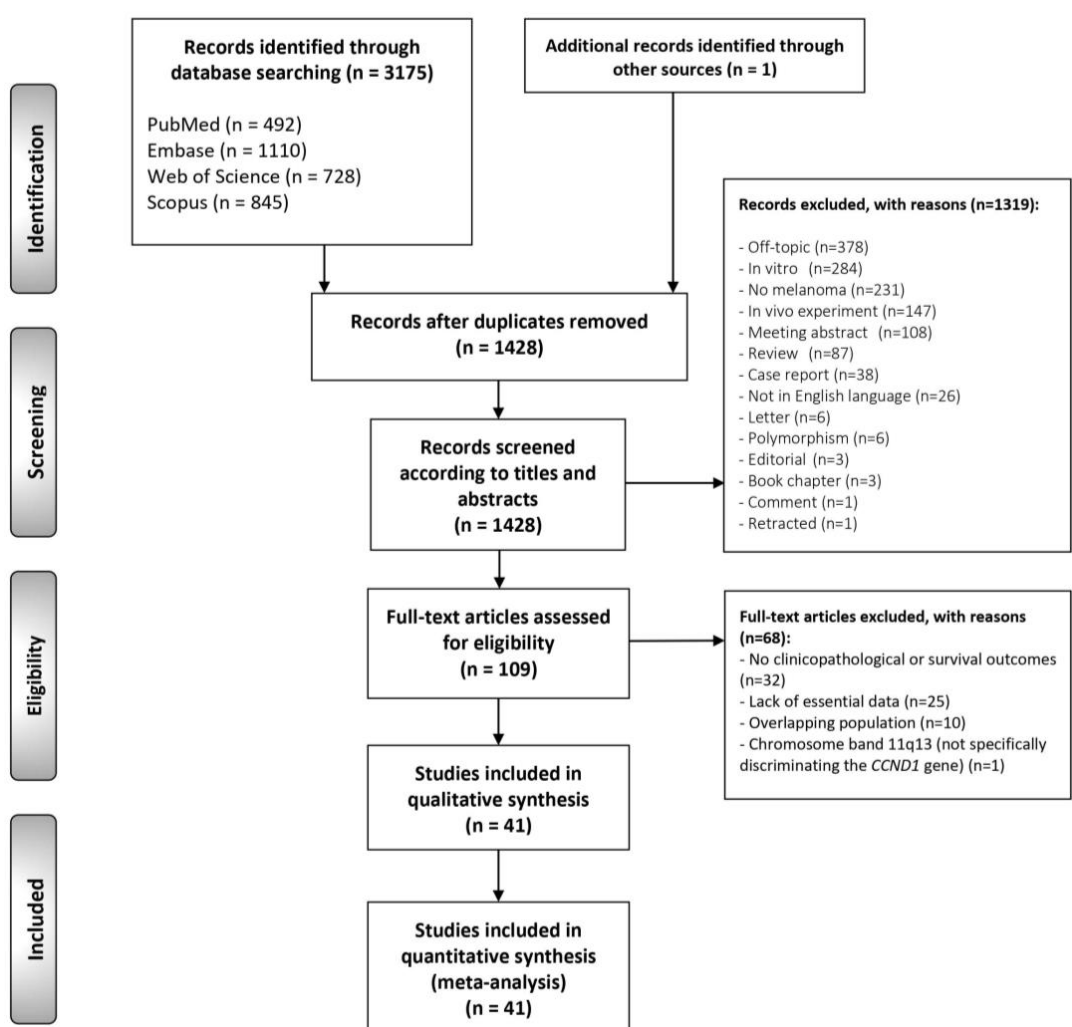
Flow diagram of the identification and selection of relevant studies, analyzing the prognostic and clinicopathological significance of *CCND1*/cyclin D1 alterations in melanomas.

The flow diagram in Figure 1 depicts search, study selection process, and the results obtained. A total of 3175 records published before September 2019 were retrieved from PubMed (*n* = 492), Embase (*n* = 1110), Web of Science (*n* = 728), and Scopus (*n* = 845). One additional record was also identified handsearching the references lists of the studies included. After duplicates removal, 1428 studies were considered potentially eligible. After the titles and abstracts screening, 109 records were selected for full-text evaluation, of which 68 did not meet all inclusion criteria, leaving a final sample of 41 studies (the references of the studies included in the systematic review and meta-analysis are listed in the Supplementary Materials, pp. 56–59).

### 3.2. Study Characteristics

Table 1 summarizes the main characteristics of the 41 studies analyzing the prognostic and clinicopathological implications of CCND1/cyclin D1 upregulation in 3451 melanomas. Cyclin D1 protein overexpression was assessed by 21 studies in 1473 melanomas (range: 7–245); CCND1 gene amplification was also analyzed by 22 studies in 2096 melanomas (range: 6–514). Two studies analyzed both CCND1/cyclin D1 alterations (118 melanomas) [24,51]. The studies were conducted in 6 continents (Europe, 17 studies/1727 melanomas; North America, 12 studies/498 melanomas; Asia, 5 studies/893 melanomas; South and Central America, 3 studies/110 melanomas; and two global multicentric studies, 60 melanomas) and 17 countries (Australia, 2 studies; Brazil, 1 study; China, 3 studies; France, 1 study; Germany, 4 studies; Greece, 1 study; Hungary, 2 studies; Italy, 3 studies; Japan, 2 studies; global multicentric, 2 studies (Australia-USA, and Germany-Japan-South Korea-USA); South and Central America multicentric, 2 studies (Bolivia-Brazil and Brazil-Guatemala-Mexico-Peru); Norway, 2 studies; Spain, 3 studies; Switzerland, 1 study; and USA, 12 studies). Table S2 (Supplementary Materials, pp. 6–12) exhibits in more detail the characteristics (type of melanoma, affected sites, and methods) gathered from each study.

### 3.3. Qualitative Evaluation

**Figure 2:**
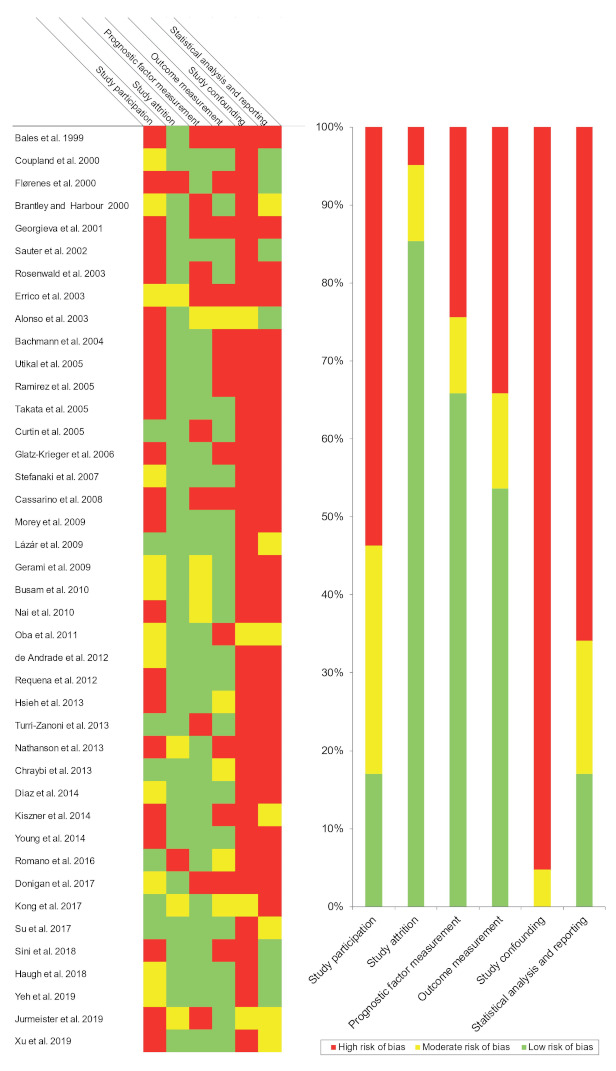
Evaluation of the risk of bias using the Quality in Prognosis Studies (QUIPS) tool (the references cited in this figure are listed in Supplementary Materials).

Qualitative analysis was conducted using the QUIPS tool, which evaluates potential sources of bias in six domains (Figure 2).

Study participation. The studies had a high (53.66%), moderate (29.27%), or low (17.07%) risk of bias (Figure 2), related to the lack of reporting relevant data (type of melanomas, affected site, age, sex, recruitment period, and/or relevant parameters with prognostic implications, e.g., Breslow thickness).

Study attrition. The studies evaluated had a high (4.88%), moderate (9.76%), or low (85.36%) risk of bias (Figure 2), mainly due to the lack of information on the follow-up period. The attempt to gather information on patients lost in the follow up period was not described by the studies.

Prognostic factor measurement. The studies had a high (24.39%), moderate (9.76%), or low (65.85%) risk of bias (Figure 2). The biases encountered were due to insufficient information on the techniques used to measure the levels of amplification or overexpression (e.g., laboratory methods or scoring system) or questionable methodologies (e.g., use of inappropriate cutoff points). In immunohistochemical studies, insufficient information about anti-cyclin D1 antibodies, immunopositivity pattern (nuclear or cytoplasmic), or the failure to report images of the technique.

Outcome measurement. The studies had a high (34.15%), moderate (12.19%), or low (53.66%) risk of bias (Figure 2), due to failure to define the survival outcomes evaluated (this is essential, due to the lack of international consensus on survival endpoints). Furthermore, the reporting of N and M status in combination, or an inadequate measurement of prognostic parameters (e.g., optimized cut-off points for clinicopathological variables to obtain significant *p*-values).

Study confounding. The studies had a high (95.24%) or moderate (4.76%) risk of bias (Figure 2), due to the failure considering potential confounders. None of the studies defined a priori the confounding factors under evaluation or a posteriori discussed potentially candidate factors or the biological principles by which they might distort the impact of CCND1/cyclin D1 upregulation on study parameters.

Statistical analysis and reporting. The studies had a high (65.86%), moderate (17.07%), or low (17.07%) risk of bias (Figure 2) due to selective outcome reporting, lack of essential information in survival analysis (i.e., Kaplan–Meier courves or hazard ratios with confidence intervals), suspected data errors, or an inappropriate statistical analysis.

## 4. Quantitative Evaluation (Meta-Analysis)

### 4.1. Association between CCND1/Cyclin D1 Upregulation and Cutaneous Melanoma

#### 4.1.1. Upregulation Frequency

Upregulation was a frequent phenomenon in acral melanomas (overexpression: PP = 62.07%, 95% CI = 31.17–89.27; amplification: PP = 25.06%, 95% CI = 15.80–35.44), and less frequent—although also present—in nodular (overexpression: PP = 13.69%, 95% CI = 2.33–29.67; amplification: PP = 22.66%, 95% CI = 3.65–48.29), superficial spreading (overexpression: PP = 36.72%, 95% CI = 17.57–57.90; amplification: PP = 15.79%, 95% CI = 3.44–32.85), and lentigo malignant melanomas (overexpression: PP = 52.24%, 95% CI = 33.36–70.82; amplification: PP = 16.16%, 95% CI = 2.53–35.62) (Table 2, Figures S1–S4, Supplementary Materials, pp. 13–16). Although considerable degrees of heterogeneity were reached for the alterations analyzed together, the subgroups were more homogeneous after applying the stratification by overexpression and amplification.

#### 4.1.2. Overall Survival (OS)

A non-significant association was found for both cyclin D1 overexpression (HR = 1.00, 95% CI = 0.64–1.58, *p* = 0.99) and CCND1 amplification (HR = 1.32, 95% CI = 0.81–1.52, *p* = 0.11) (Table 2, Figure S5, Supplementary Materials, p. 17). There was no evidence of heterogeneity among studies (*p*_het_ = 0.30, I^2^ = 16.7%). In the stratified analyses by geographic area and immunohistochemical pattern, the results did not vary significantly among the subgroups under analysis (Table 2, Figures S6 and S7, Supplementary Materials, pp. 18, 19). Additional planned analyses (by anti-cyclin D1 antibody and cutoff point) were not possible for any parameter due to the heterogeneous and small amount of data reported by the studies.

#### 4.1.3. Disease-Free Survival (DFS)

*CCND1*/cyclin D1 upregulation was not significantly associated with poor DFS (HR = 1.45, 95% CI = 0.60–3.51, *p* = 0.41) (Table 2, Figure S8, Supplementary Materials, p. 20). As only 2 studies were included for this variable, subgroups analyses were not performed.

#### 4.1.4. Breslow Thickness

**Figure 3:**
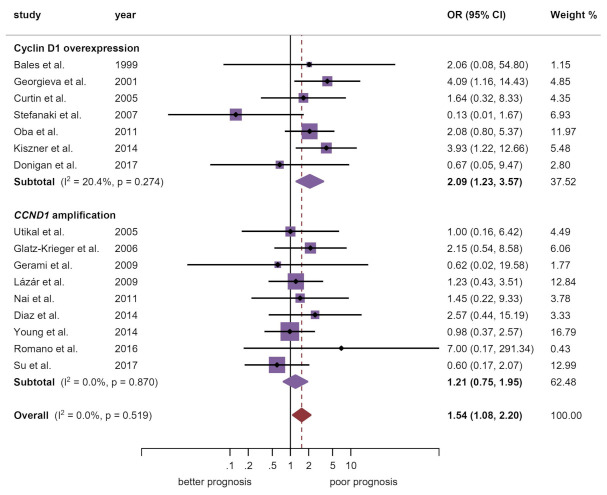
Forest plot graphically representing the association between *CCND1*/cyclin D1 alterations and Breslow thickness in cutaneous melanomas. OR, odds ratio; CI, confidence intervals. Fixed-effect model (Mantel–Haenszel method) pooling odds ratios (the references cited in this figure are listed in Supplementary Materials).

A significant large effect size (OR = 2.09, 95% CI = 1.23–3.57, *p* = 0.007) was found with cyclin D1 overexpression, while this association was not found for gene amplification (OR = 1.21, 95% CI = 0.75–1.95, *p* = 0.43) (Table 2, Figure 3). Heterogeneity across studies was not evident (*p*_het_ = 0.52, I^2^ = 0.0%). In the stratified analysis, the European (OR = 1.91, 95% CI = 1.15–3.19, *p* = 0.01) subgroup maintained the significant association (Table 2, Figure S9, Supplementary Materials, p. 21).

#### 4.1.5. Distance Metastatic vs. Primary Tissue

A strong significant association was found between cyclin D1 and its overexpression in primary tissues (OR = 0.53, 95% CI = 0.40–0.71, *p* < 0.001). On the other hand, a weaker association was found for *CCND1* amplification in metastatic tissue (OR = 1.70, 95% CI = 1.01–2.85, *p* = 0.04). Although a considerable heterogeneity degree was observed (*p*_het_ < 0.001; I^2^ = 68.5%), the subgroups were more homogeneous after being stratified by alteration, and heterogeneity lost its significance for gene amplification (*p*_het_ = 0.16, I^2^ = 35.5%) (Table 2, Figure S21, Supplementary Materials, p. 33). In the stratified analyses, the European (OR = 0.73, 95% CI = 0.55–0.97, *p* = 0.03) subgroup and the nuclear immunopositive pattern (OR = 0.56, 95% CI = 0.41–0.76, *p* < 0.001) strongly maintained their significant associations with primary tissues upregulation (Table 2, Figures S22 and S23, Supplementary Materials, pp. 33, 34).

#### 4.1.6. Additional Clinicopathological Variables

Neither cyclin D1 overexpression nor *CCND1* amplification were significantly associated with the other variables analyzed (ulceration, N and M status, clinical stage, mitotic rate, Clark levels and type of melanoma; Table 2, Figures S11–S19, Supplementary Materials, pp. 23–31). A considerable significant degree of heterogeneity was only reached by clinical stage (*p*_het_ = 0.03, I^2^ = 70.5%). Due to the low number of studies included in these variables, subgroups analyses were only performed for type of melanoma. Nevertheless, relevant subpopulations harboring higher levels of cyclin D1 or *CCND1* amplification were not found (Table 2, Figures S17–S19, Supplementary Materials, pp. 29–31).

### 4.2. Association between CCND1/Cyclin D1 Upregulation and Uveal Melanoma

Upregulation frequency. Upregulation was very inconsistent across studies in uveal melanomas (PP = 14.42%, 95% CI = 0.00–46.91; *p*_het_ < 0.001, I^2^ = 95.91%) (Table 2, Figure S24, Supplementary Materials, p. 36). Due to the low number of studies included (*n* = 3), heterogeneity sources—performing subgroups analyses—could not be investigated or explained.

Prognostic and clinicopathological variables. Meta-analyses could only be performed for the variables largest basal dimension (N.S) and pathologic cell type, in which the epithelioid shaped cells showed a significant and imprecise association (very large magnitude of effect) with *CCND1*/cyclin D1 upregulation (OR = 4.59, 95% CI = 1.47–14.36, *p* = 0.009) (Table 2, Figures S25 and S26, Supplementary Materials, pp. 37, 38).

### 4.3. Association between CCND1/Cyclin D1 Upregulation and Mucosal Melanoma

#### 4.3.1. Upregulation Frequency

Upregulation was a very frequent phenomenon in mucosal melanomas (overexpression: PP = 75.69%, 95% CI = 55.48–91.63; amplification: PP = 25.08%, 95% CI = 17.29–33.66), and the subgroups of melanomas arising in the oral cavity showed the highest prevalence of the present meta-analysis (PP = 80.05%, 95% CI = 66.67–90.94) (Table 2, Figures S27 and S28, Supplementary Materials, pp. 39, 40). These frequencies were very inconsistent across studies and responsible sources of heterogeneity could not be detected (*p*_het_ < 0.001, I^2^ = 86.76%).

#### 4.3.2. Prognostic and Clinicopathological Variables

Meta-analyses could only be performed for tumor thickness, status M, and necrosis, but none of the variables was statistically associated with CCND1/cyclin D1 upregulation (Table 2, Figures S29–S32, Supplementary Materials, pp. 41–44).

### 4.4. Quantitative Evaluation (Secondary Analyses)

#### 4.4.1. Sensitivity Analysis

In general, the overall results did not substantially vary after the sequential repetition of meta-analyses, omitting one study each turn (“leave-one-out” method) (Tables S3–S8, Supplementary Materials, pp. 50–55). Therefore, these sensitivity analyses series suggest that the precedent pooled estimations are robust and do not depend on the influence of a particular individual study.

#### 4.4.2. Analysis of Small-Study Effects

**Figure 4:**
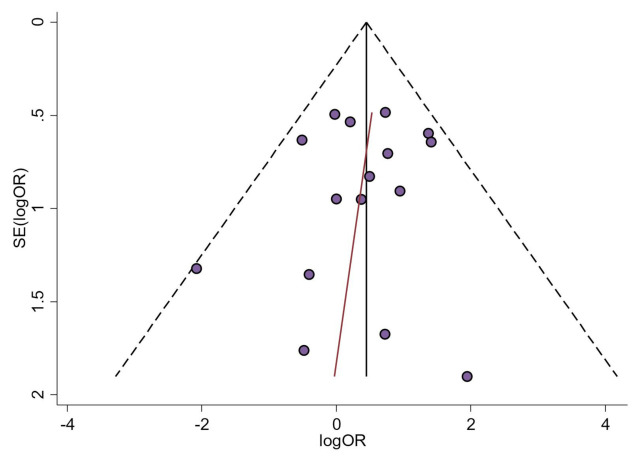
A funnel plot of estimated logOR against its standard error, graphically representing the analysis of “small-study” effects on Breslow thickness. The black vertical line corresponds to the pooled estimated prevalence. The two diagonal intermittent lines represent the pseudo-95% confidence interval. The purple circles represent the published studies. The blue line represents the fitted line corresponding to Egger’s regression test (*p*_Egger_ = 0.56) for funnel-plot asymmetry. SE, standard error; OR, odds ratio.

Visual inspection of the asymmetry of funnel plots and of the statistical tests conducted for the same purpose confirmed the absence of small-study effects on the variables OS (*p*_Egger_ = 0.82), Breslow thickness (*p*_Egger_ = 0.56; Figure 4), distance metastatic vs. primary tissue (*p*_Egger_ = 0.17), type of melanoma (*p*_Egger_ = 0.23), ulceration (*p*_Egger_ = 0.37), and Clark levels (*p*_Egger_ = 0.43) in cutaneous melanomas (Figures S33–S37, Supplementary Materials, pp. 45–49), for which biases (e.g., publication bias) can be ruled out. The variables DFS, N, and M status, ganglionar metastatic vs. primary tissue, clinical stage and mitotic rate in cutaneous melanomas (idem for uveal and mucosal) did not meet the conditions for the statistical analysis of small-study effects, since a low number of studies were included in their respective meta-analyses.

## 5. Discussion

This systematic review and meta-analysis of 41 studies and 3451 patients, shows *CCND1*/cyclin D1 upregulation as a common oncogenic alteration in cutaneous, mucous, and uveal melanomas, and we carry out a comparative study of the alterations of this oncogene and its product in these different types of melanomas under the hypothesis that perhaps the upregulation of *CCND1*/cyclin D1 may be different in these types of melanomas and to some extent justify their different clinical behavior and prognosis. In cutaneous melanomas, the highest frequency was observed in lentigo malignant melanoma (37.73% of cases) followed by acral melanoma, superficial spreading melanoma, and nodular melanoma (30.90%, 24.71%, and 19.19%, respectively). In uveal melanomas, *CCND1*/cyclin D1 upregulation is observed in 14.41% of cases, while in mucous melanomas, appeared in 42.33% of cases, with melanoma of the oral cavity presenting the highest frequency—80.05% of cases. It is interesting to note that also in the most frequent tumor of the oral cavity, the oral squamous cell carcinoma, cyclin D1 is frequently overexpressed which has a strong impact on the patients prognosis [19,52]. Although our meta-analysis in oral mucosa melanomas is based on a small number of studies, these results justify more experimental studies that increase the scientific evidence on the frequency of alterations of *CCND1*/cyclin D1 and that ratify its prognostic implications in these type of melanomas. The results indicate that the positive regulation of this gene and its protein, which are key to promoting the progression of the cell cycle, is a oncogenic mechanism probably involved in the malignant transformation of melanocytic cells, justifying its frequent presentation by the number of oncogenic aberrations—gene amplification, mutations, polymorphisms, chromosomal translocations—and molecular pathways that conclude with the upregulation of *CCND1*/cyclin D1 in melanomas [22]; however, this upregulation, although frequent, was not different in the different types of cutaneous melanomas. Among the latter, in recent years, upregulation of *CCND1*/cyclin D1 dependent on activation of the MAPK pathway (Ras/Raf/MEK/Erk) has been reported, with the MAPK pathway constitutive activation being observed in 90% of melanomas [53], in which the BRaf and NRas mutations [54,55] are especially important. Mechanisms of upregulation dependent on activation of the PI3K/Akt pathway have also been reported, where mTor activates the mRNA *CCND1* translation and promotes the formation of cyclin D1/CDK4 and CDK6 complexes, involved in cell cycle progression [55]. Furthermore, *CCND1*/cyclin D1 upregulation mechanisms linked to the failure of senescence associated with the actions of tumor suppressor gene CDKN2A/p16INK4, mechanisms linked to alterations in growth receptors, mechanisms linked to activation of the Wnt/βcatenin pathway, and mechanisms linked to epigenetic regulations of *CCND1*/cyclin D1 have been also documented [22]. Usually, papers focusing on *CCND1*/cyclin D1 upregulation do so by analyzing *CCND1* gene amplification and/or cyclin D1 immunohistochemical overexpression protein. Our results demonstrate a significantly higher frequency of cyclin D1 immunohistochemical overexpression in melanomas in comparison with the *CCND1* gene amplification, which is probably related with the multiple and frequent molecular pathways, previously discussed, that lead to upregulate *CCND1*/cyclin D1 activity in the absence of gene amplification. In our meta-analysis, *CCND1* amplification was most frequently upregulated in acral melanomas (25.06%, 95% CI = 15.80–35.44; out of 85 cases) than in other cutaneous melanomas subtypes. A recently published integrative analysis from the “Pan-Cancer Analysis of Whole Genomes” of the Consortium of the International Cancer Genome Consortium (ICGC) reported chromothripsis involving *CCND1* on chromosome 11 [56]. This alteration was also frequently observed in 11/21 acral melanomas (52.38% of cases), and in 10/86 cutaneous melanomas (11.63% of cases), most of which harboring gene amplifications [56]. Therefore, *CCND1* focal amplification could be considered as a potential driver in the context of chromothripsis in acral melanomas. The Cancer Genome Atlas (TCGA) also confirmed variations in copy-number gains across melanoma subtypes. *CCND1* amplification was also determined as one of the most relevant copy-number alterations in Triple-WT subtype (11% of cases were focally amplified), with a statistically significant increase (Fisher’s exact test: *p* < 0.01) compared to BRAF (3%, out of 150 cases), RAS (8%, out of 92 cases), and NF1 (0%, out of 58 cases) [57]. Future somatic copy number alteration bioinformatics analyses are needed in melanomas in order to comprehensively describe the genomic aberrations located in 11q13 chromosomal band—and singularly *CCND1* amplification—from large-scale sequencing-based datasets (e.g., TCGA and ICGC projects). In relation to cyclin D1 overexpression, whose relative frequency was more common, our results are relevant since the immunohistochemistry is a simple, inexpensive, and commonly used technique in pathology laboratories, which facilitates and probably advises its routine incorporation into the melanoma diagnostic process. This meta-analysis has also revealed that the true value of immunohistochemistry is obtained when the nuclear protein expression is considered (*p* < 0.01; OR = 0.56, 95% CI = 0.41–0.76). Only one study has evaluated both the nuclear and cytoplasmic expression of cyclin D1 in melanomas, so it has not been possible to obtain data to meta-analyze; however, our research group has recently reported in oral squamous cell carcinoma [58], the association of cytoplasmic cyclin D1 overexpression with the development of actin-based protrusive structures—invadopodia and lamellipodia—in tumor cells, which functions are related with the acquisition of invasive advantages of malignant cells. In our opinion, it is advisable to expand the knowledge about the meaning and true value of immunolocalization of cyclin D1 in the cytoplasm in melanoma. The frequent *CCND1*/cyclin D1 upregulation in melanomas has stimulated the research of cyclin D1 inhibitors as a therapeutic tool [59]. Cyclin D1 complies with the concept of oncogenic addiction described by Weinstein and Joe [60]—i.e., dependency of carcinogenic cells on the expression of some oncoproteins, which allows them to survive and maintain their proliferation—which implies that the therapeutic blockage of a single protein could have benefits in the treatment of the tumor. Accordingly, cyclin D1 is a candidate protein that can be targeted in different ways: by inhibition of chromosome band 11q13 and/or *CCND1*; by direct cyclin D1 inhibition; by CDK inhibition, acting upstream on melanoma-related pathways that involve cyclin D1; or by combining agents that act against cyclin D1 with other antitumor drugs. There are reports on several drugs that inhibit these pathways being tested in melanoma (it is advisable to consult Table 1 of [22]).

Our meta-analysis demonstrates associations between CCND1/cyclin D1 upregulation and some clinicopathological features of melanoma, although no value has been shown in predicting patient survival. Among them, stands out the significant association found between *CCND1*/cyclin D1 upregulation and Breslow thickness (*p* = 0.02) revealed essentially through immunohistochemical overexpression of the protein (*p* = 0.007; OR = 2.09, 95% CI = 1.23–3.57), while the *CCND1* gene amplification does not show association (*p* = 0.43). This observation could be explained, as mentioned above, by the multiple active oncogenic pathways in melanoma that converge in CCND1/cyclin D1 upregulation without genetic amplification. Likewise, a significantly higher frequency of *CCND1*/cyclin D1 abnormalities has been observed in the primary tumor compared to distant metastases (*p* = 0.004), revealed also by immunohistochemical overexpression of the protein (*p* < 0.001; OR = 0.53, 95% CI = 0.40–0.71), while gene amplification, on the contrary, appeared more frequently in distant metastases (*p* = 0.04; OR = 1.70, 95% CI = 1.01–2.85) and not in the primary tumor, which could be indicating gene amplifications as late events in melanocytic oncogenesis probably linked to a phenomenon of genomic instability in highly proliferative cell clones. This observed event could also be explained by the previously commented chromothripsis on chromosome 11 associated to CCND1. These alterations seem to be early events in the development of acral melanomas, but perhaps could be late events in cutaneous melanomas, so the timing of amplification events could be different between cutaneous and acral melanomas. On the other hand, cyclin D1 overexpression could be an early event not associated to gene amplification as occurring in other cancers [19]. If confirmed in future studies, cyclin D1 overexpression could be an early event in melanomas due to the activation of oncogenic aberrant upstream pathways (e.g., MAPK, PI3K, and/or WNT canonical) [6,22]. Taken together—greater expression in primary tumor vs. distant metastases and association with Breslow thickness—these data seem to indicate that *CCND1*/cyclin D1 upregulation is especially influential in the local growth and expansion of the tumor. On the other hand, the significantly lower frequency of cyclin D1 overexpression in distant metastases compared to the primary tumor is probably revealing its less important role in the development of the metastatic process, which could explain its lack of value as a predictor of survival in melanomas, since distant metastases are the main cause of death in this tumor.

According to our qualitative evaluation, carried out using the Quality in Prognosis Studies (QUIPS) tool of the Cochrane Prognosis Methods Group [38], all studies were not conducted with the same rigor. The domain “study confounding” harbored the highest risk of potential bias, caused by the failure considering or measuring confounding factors. Future studies assessing the prognostic and clinicopathological significance of CCND1/cyclin D1 in melanomas should consider the potential biases reported in the present systematic review and meta-analysis, using the QUIPS tool to improve the validity of findings and facilitating comparisons.

Some potential limitations of our meta-analysis should be discussed. First, the restriction to studies published in English may imply a loss of information published in other languages, which would have been missed. Second, consistent heterogeneity was observed in some variables (mainly in meta-analyses of proportions). In order to overcome this limitation, a random-effect statistical model was applied in these meta-analyses. We also conducted several secondary stratified analyses, obtaining more homogeneous subgroups of studies. One potential source of methodological and statistical heterogeneity was the combination *CCND1* amplification and cyclin D1 overexpression at protein level (assessed using immunohistochemistry), as observed after several stratifications. In general, the amplification-based meta-analyses seem less significant in many-analyses and the reason for this phenomenon could be due to various explanations among which are the following: *CCND1* amplification was not evaluated homogeneously across the studies, so a heterogeneity extent could be due to the inherent differences of the wide range of experimental methods; Primary-level studies also presented differences in their statistical study designs, and some considered the number of copies as a continuous variable (i.e., means of copies with standard deviations), so when papers did not openly report individual patient data, those reporting continuous variables had to be excluded under the “lack of essential data” criterion, since in our meta-analysis, we estimated effect sizes for categorical variables (i.e., odds ratios, hazard ratios, and pooled proportions); Finally, the non-significant results in amplification-based meta-analyses could also be due to the low sample sizes of some included cohorts, with statistical analyses in underpowered conditions, yielding non-significant results mainly due to type II errors (i.e., false negative results), so, another important recommendation of the present work is the development of future better designed studies—preferably prospective cohorts—assessing CCND1 amplification on higher sample sizes. Another relevant recommendation, as previously commented, is the necessity of future integrative bioinformatics analyses in melanomas in order to comprehensively describe the somatic copy number alteration linked to 11q13/CCND1 from massive datasets (e.g., TCGA, ICGC projects, or from Gene Expression Omnibus (GEO). Specific bioinformatics study design could better respond to additional research questions that could not be addressed in this meta-analysis design (e.g., relationships between CCND1 amplification and CCND1 RNA expression). Third, some variables (e.g., mitotic rate or ulceration) were reported by a low number of studies and their meta-analyses were probably underpowered to detect significant differences. Future studies should further elucidate the potential influence of *CCND1*/cyclin D1 on these parameters. Finally, some studies reported a low amount of data (e.g., anti-cyclin D1 antibody or cutoff points), limiting additional analyses. The lack of essential information in the survival analysis (i.e., HR or 95% CI) was countered estimating HR from the data provided by these studies, following the methodology of Tierney et al. [41] and Parmar et al. [40]. Despite the above limitations, the results of our meta-analysis are robust, as demonstrated by sensitivity and small-study effects analyses, and as depicted in forest plots.

## 6. Conclusions

In conclusion, CCND1/cyclin D1 upregulation is a common molecular oncogenic alteration in melanomas that probably favors the growth and expansion of the primary tumor. This upregulation is mainly a consequence to the overexpression of the cyclin D1 protein, and not to gene amplification, which probably suggests the inclusion of the immunohistochemical expression of cyclin D1 in the global evaluation of melanomas, and opens the possibility of its use as a therapeutic target.

## Figures and Tables

**Table 1:** Summarizes the main characteristics of reviewed studies. Table S2 (in Supplementary Materials, pp. 6–12) exhibits in detail the characteristics (type of melanoma, affected sites, and methods) of each study. * —Two studies analyzed both *CCND1*/cyclin D1 alterations (118 melanomas).

Total	41 Studies
Year of publication	1999–2019
Number of melanomas analyzed	
Total	3451
Sample size, range	6–514
Cyclin D1 protein overexpression *	
Total	21 studies (1473 melanomas)
Sample size, range	7–245
CCND1 gene amplification *	
Total	22 studies (2096 melanomas)
Sample size, range	6–514
Geographical region	
Europe	17 studies (1727 melanomas)
North America	12 studies (498 melanomas)
Asia	5 studies (893 melanomas)
South and Central America	3 studies (110 melanomas)
Australia	2 studies (163 melanomas)
Global multicentric	2 studies (60 melanomas)
Total	6 continents, 17 countries

**Table 2:** Cyclin D1/CCND1 upregulation in melanoma: frequency, clinicopathological, and prognostic significance.

Meta-Analyses	No. of Studies	No. of Cases	Wt	Stat. Model	Pooled Data		Hetero Geneity		Supplementary Materials ^a^
					ES (95% CI)	*p*-value	*p*_het_	I^2^(%)	
**CUTANEOUS MELANOMA**									
**Frequency of cyclin D1/*CCND1* upregulation**									
Nodular melanoma ^b^	15	219	D-L	REM	PP = 19.19% (7.12–34.06)	-	<0.001	70.63	Figure S1, p. 13
Nodular melanoma by alteration ^c^									Figure S1, p. 13
Cyclin D1 overexpression	7	103	D-L	REM	PP = 13.69% (2.33–29.67)	-	0.13	39.69	
CCND1 amplification	8	116	D-L	REM	PP = 22.66% (3.65–48.29)	-	<0.001	80.92	
Superficial spreading melanoma ^b^	15	553	D-L	REM	PP = 24.71% (13.29–37.84)	-	<0.001	86.26	Figure S2, p. 14
Superficial spreading melanoma by alteration ^c^									Figure S2, p. 14
Cyclin D1 overexpression	7	325	D-L	REM	PP = 36.72% (17.57–57.90)	-	<0.001	88.53	
CCND1 amplification	8	228	D-L	REM	PP = 15.79% (3.44–32.85)	-	<0.001	83.30	
Lentigo malignant melanoma ^b^	5	63	D-L	REM	PP = 34.73% (10.61–63.09)		<0.001	76.77	Figure S3, p. 15
Lentigo malignant melanoma by alteration ^c^						-			Figure S3, p. 15
Cyclin D1 overexpression	2	29	D-L	REM	PP = 52.24% (33.36–70.82)	-	-	-	
*CCND1* amplification	3	34	D-L	REM	PP = 16.16% (2.53–35.62)	-	0.27	23.42	
Acral melanoma ^b^	7	98	D-L	REM	PP = 30.90% (18.27–44.86)	-	0.15	36.98	Figure S4, p. 16
Acral melanoma by alteration ^c^									Figure S4, p. 16
Cyclin D1 overexpression	2	13	D-L	REM	PP = 62.07% (31.17–89.27)	-	-	-	
*CCND1* amplification	5	85	D-L	REM	PP = 25.06% (15.80–35.44)	-	0.44	0.00	
**Survival parameters**									
Overall survival ^d^	7	1022	D-L	REM	HR = 1.11 (0.81–1.52)	0.51	0.30	16.7	Figure S5, p. 17
Overall survival by alteration ^e^									Figure S5, p. 17
Cyclin D1 overexpression	5	399	D-L	REM	HR = 1.00 (0.64–1.58)	0.99	0.26	24.3	
*CCND1* amplification	2	623	D-L	REM	HR = 1.32 (0.81–1.52)	0.11	-	-	
Overall survival by geographic area ^e^									Figure S6, p. 18
Asian	2	592	D-L	REM	HR = 1.30 (0.92–1.82)	0.13	-	-	
Non-Asian	5	430	D-L	REM	HR = 1.03 (0.63–1.69)	0.90	0.23	28.3	
Overall survival by IHQ pattern ^e^									Figure S7, p. 19
Nuclear	3	235	D-L	REM	HR = 0.77 (0.50–1.17)	0.22	0.58	0.0	
Nuclear and cytoplasmic	1	78	-	-	-	-	-	-	
Not available	1	86	-	-	-	-	-	-	
**Clinicopathological parameters**									
Disease-free survival ^d^	2	70	D-L	REM	HR = 1.45 (0.60–3.51)	0.41	-	-	Figure S8, p. 20
Breslow thickness ^d^	16	760	M-H	FEM	OR = 1.54 (1.08–2.20)	0.02	0.52	0.0	Manuscript, Figure 3
Breslow thickness by alteration ^e^									
Cyclin D1 overexpression	7	264	M-H	FEM	OR = 2.09 (1.23–3.57)	0.007	0.27	20.4	
*CCND1* amplification	9	496	M-H	FEM	OR = 1.21 (0.75–1.95)	0.43	0.87	0.0	
Breslow thickness by geographic area ^e^									Figure S9, p. 21
Asia	2	120	M-H	FEM	OR = 1.31 (0.63–2.74)	0.47	-	-	
Australia	1	143	-	-	-	-	-	-	
Europe	7	355	M-H	FEM	OR = 1.91 (1.15–3.19)	0.01	0.21	28.4	
Global multicentric	1	39	-	-	-	-	-	-	
North America	4	41	M-H	FEM	OR = 1.36 (0.31–5.90)	0.68	0.73	0.0	
South America	1	62	-	-	-	-	-	-	
Breslow thickness by IHQ pattern ^e^									Figure S10, p. 22
Nuclear	3	89	M-H	FEM	OR = 1.83 (0.72–4.61)	0.20	0.06	65.3	
Nuclear and cytoplasmic	1	78	-	-	-	-	-	-	
Not available	3	97	M-H	FEM	OR = 2.41 (0.98–5.90)	0.06	0.41	0.0	
Ulceration ^d^	4	759	M-H	FEM	OR = 0.99 (0.70–1.42)	0.97	0.40	0.0	Figure S11, p. 23
N Status ^d^	2	16	M-H	FEM	OR = 0.77 (0.06–9.14)	0.83	-	-	Figure S12, p. 24
M Status ^d^	2	34	M-H	FEM	OR = 0.86 (0.15–5.02)	0.87	-	-	Figure S13, p. 25
Clinical Stage ^d^	3	585	M-H	FEM	OR = 1.13 (0.78–1.66)	0.51	0.03	70.5	Figure S14, p. 26
Mitotic rate ^d^	2	117	M-H	FEM	OR = 1.66 (0.72–3.84)	0.23	0.71	0.0	Figure S15, p. 27
Clark levels ^d^	7	195	M-H	FEM	OR = 1.10 (0.59–2.06)	0.76	0.34	12.1	Figure S16, p. 28
Type (nodular vs. SSM/LMM/AM) ^d^	13	646	M-H	FEM	OR = 0.84 (0.52–1.35)	0.47	0.14	30.4	Figure S17, p. 29
Type by alteration ^e^									Figure S17, p. 29
Cyclin D1 overexpression	6	337	M-H	FEM	OR = 0.53 (0.26–1.08)	0.08	0.30	17.1	
*CCND1* amplification	7	309	M-H	FEM	OR = 1.29 (0.66–2.51)	0.45	0.23	26.4	
Type by geographic area ^e^									Figure S18, p. 30
Australia	1	9	-	-	-	-	-	-	
Europe	4	275	M-H	FEM	OR = 1.54 (0.80–2.97)	0.19	0.52	0.0	
Global multicentric	1	33	-	-	-	-	-	-	
North America	6	268	M-H	FEM	OR = 0.34 (0.14–0.84)	0.02	0.08	50.1	
South America	1	61	-	-	-	-	-	-	
Type by IHQ pattern ^e^									Figure S19, p. 31
Nuclear	5	304	M-H	FEM	OR = 0.54 (0.26–1.11)	0.09	0.20	33.5	
Not available	1	33	-	-	-	-	-	-	
Lymph node metastasis vs. primary tissue ^d^	2	184	M-H	FEM	OR = 1.07 (0.43–2.68)	0.88	0.21	35.7	Figure S20, p. 32
Distance metastasis vs. primary tissue ^d^	16	1651	M-H	FEM	OR = 0.70 (0.55–0.89)	0.004	<0.001	68.5	Figure S21, p. 33
By alteration ^e^									Figure S21, p. 33
Cyclin D1 overexpression	9	987	M-H	FEM	OR = 0.53 (0.40–0.71)	<0.001	<0.001	71.8	
*CCND1* amplification	7	664	M-H	FEM	OR = 1.70 (1.01–2.85)	0.04	0.16	35.5	
By geographic area ^e^									Figure S22, p. 34
Asia	2	122	M-H	FEM	OR = 0.42 (0.18–1.00)	0.05	-	-	
Australia	1	20	-	-	-	-	-	-	
Europe	9	1237	M-H	FEM	OR = 0.73 (0.55–0.97)	0.03	<0.001	73.0	
North America	4	272	M-H	FEM	OR = 0.72 (0.39–1.34)	0.30	0.001	82.2	
By IHQ pattern ^e^									Figure S23, p. 35
Nuclear	7	826	M-H	FEM	OR = 0.56 (0.41–0.76)	<0.001	<0.001	78.3	
Nuclear and cytoplasmic	1	101	-	-	-	-	-	-	
Not available	1	60	-	-	-	-	-	-	
**UVEAL MELANOMA**									
**Frequency of cyclin D1/*CCND1* upregulation**									
Uveal melanoma ^b^	3	197	D-L	REM	PP = 14.42% (0.00–46.91)	-	<0.001	95.91	Figure S24, p. 36
**Survival parameters**									
OS	0	0	-	-	-	-	-	-	-
DFS ^d^	1	45	-	-	-	-	-	-	-
**Clinicopathological parameters**									
Thickness	1	32	-	-	-	-	-	-	-
Distance metastasis	1	82	-	-	-	-	-	-	-
Sclera infiltration	1	45	-	-	-	-	-	-	-
Largest basal dimension^d^	2	77	M-H	FEM	OR = 2.78 (0.950–8.16)	0.06	-	-	Figure S25, p. 37
Pathology (epithelioid vs. spindle/mixed) ^d^	2	77	M-H	FEM	OR = 4.59 (1.47–14.36)	0.009	-	-	Figure S26, p. 38
**MUCOSAL MELANOMA**									
**Frequency of cyclin D1/*CCND1* upregulation**									
Mucosal melanoma ^b^	12	356	D-L	REM	PP = 42.33% (27.24–58.12)	-	<0.001	86.76	Figure S27, p. 39
Mucosal melanoma by alteration ^c^									Figure S27, p. 39
Cyclin D1 overexpression	4	94	D-L	REM	PP = 75.69% (55.48–91.63)	-	0.01	74.22	
*CCND1* amplification	8	262	D-L	REM	PP = 25.08% (17.29–33.66)	-	0.09	42.72	
Mucosal melanoma by anatomical site ^c^									Figure S28, p. 40
Ano-rectal	1	45	-	-	-	-	-	-	
Conjunctival	1	6	-	-	-	-	-	-	
Head and neck mixed	1	94	-	-	-	-	-	-	
Esophageal	2	29	D-L	REM	PP = 13.51% (2.47–29.52)	-	-	-	
Genitourinary	1	55	-	-	-	-	-	-	
Oral cavity	2	46	D-L	REM	PP = 80.05% (66.67–90.94)	-	-	-	
Sinonasal	4	81	D-L	REM	PP = 41.47% (14.11–71.73)	-	<0.001	86.1	
**Survival parameters**									
OS	0	0	-	-	-	-	-	-	-
DFS	0	0	-	-	-	-	-	-	-
Recurrence ^d^	2	41	M-H	FEM	OR = 0.90 (0.18–4.38)	0.89	-	-	Figure S29, p. 41
**Clinicopathological parameters**									
Thickness ^d^	4	241	M-H	FEM	OR = 1.19 (0.67–2.11)	0.56	0.17	39.6	Figure S30, p. 42
N Status	0	0	-	-	-	-	-	-	-
M Status ^d^	2	40	M-H	FEM	OR = 1.95 (0.21–18.30)	0.56	0.77	0.0	Figure S31, p. 43
Necrosis ^d^	3	65	M-H	FEM	OR = 0.90 (0.30–2.70)	0.85	0.70	0.0	Figure S32, p. 44

Abbreviations: Stat., statistical; Wt, method of weighting; ES, estimation; CI, confidence intervals; REM, random-effects model; FEM, fixed-effects model; D-L, DerSimonian and Laird method; M-H, Mantel-Haenszel method; PP, pooled proportion; HR, hazard ratio; OR, odds ratio. a- More information in the appendix; b- Proportion meta-analyses; c- Proportion meta-analyses (Subgroup analyses); d- Prognosis meta-analyses; e- Prognosis meta-analyses (Subgroup analyses).

## Data Availability

The data that supports the findings of this study are available in the Supplementary Material of this article.

## Supplementary Materials

The following are available online at https://www.mdpi.com/2072-6694/13/6/1314/s1, Table S1. Search strategy for each database, number of results, and execution date; Table S2. Characteristics of studies; Table S3. Sensitivity analysis of the studies pooled in the meta-analysis on the association between *CCND1*/cyclin D1 alterations and overall survival in cutaneous melanoma; Table S4. Sensitivity analysis of the studies pooled in the meta-analysis on the association between *CCND1*/cyclin D1 alterations and Breslow Thickness in cutaneous melanoma; Table S5. Sensitivity analysis of the studies pooled in the meta-analysis on the association between *CCND1*/cyclin D1 alterations and Ulceration in cutaneous melanoma; Table S6. Sensitivity analysis of the studies pooled in the meta-analysis on the association between *CCND1*/cyclin D1 alterations and Clark in cutaneous melanoma; Table S7. Sensitivity analysis of the studies pooled in the meta-analysis on the association between *CCND1*/cyclin D1 alterations and Type of cutaneous melanoma; Table S8. Sensitivity analysis of the studies pooled in the meta-analysis on the association between *CCND1*/cyclin D1 alterations and Distance metastasis vs primary in cutaneous melanoma; List of studies included in this systematic review and meta-analysis; and copy of protocol. Figure S1. Forest plot graphically representing the frequency of *CCND1*/cyclin D1 alterations in Nodular Melanomas; Figure S2. Forest plot graphically representing the frequency of *CCND1*/cyclin D1 alterations in Superficial Spreading Melanomas; Figure S3. Forest plot graphically representing the frequency of *CCND1*/cyclin D1 alterations in Lentigo Malignant Melanomas; Figure S4. Forest plot graphically representing the frequency of *CCND1*/cyclin D1 alterations in Acral Melanomas; Figure S5. Forest plot graphically representing the association between *CCND1*/cyclin D1 alterations and Overall Survival in Cutaneous Melanomas; Figure S6. Forest plot graphically representing the association between *CCND1*/cyclin D1 alterations and Overall Survival in Cutaneous Melanomas by geographic area; Figure S7. Forest plot graphically representing the association between *CCND1*/cyclin D1 alterations and Overall Survival in Cutaneous Melanomas by immunohistochemical pattern; Figure S8. Forest plot graphically representing the association between *CCND1*/cyclin D1 alterations and Disease-Free Survival in Cutaneous Melanomas; Figure S9. Forest plot graphically representing the association between *CCND1*/cyclin D1 alterations and Breslow Thickness in Cutaneous Melanomas by geographic area; Figure S10. Forest plot graphically representing the association between *CCND1*/cyclin D1 alterations and Breslow Thickness in Cutaneous Melanomas by immunohistochemical pattern; Figure S11. Forest plot graphically representing the association between *CCND1*/cyclin D1 alterations and Ulceration in Cutaneous Melanomas; Figure S12. Forest plot graphically representing the association between *CCND1*/cyclin D1 alterations and N status in Cutaneous Melanomas; Figure S13. Forest plot graphically representing the association between *CCND1*/cyclin D1 alterations and M status in Cutaneous Melanomas; Figure S14. Forest plot graphically representing the association between *CCND1*/cyclin D1 alterations and Clinical Stage in Cutaneous Melanomas; Figure S15. Forest plot graphically representing the association between *CCND1*/cyclin D1 alterations and Mitotic Rate in Cutaneous Melanomas; Figure S16. Forest plot graphically representing the association between *CCND1*/cyclin D1 alterations and Clark levels in Cutaneous Melanomas; Figure S17. Forest plot graphically representing the association between *CCND1*/cyclin D1 alterations and types of melanoma (nodular vs. SSM/LMM/AM) in Cutaneous Melanomas; Figure S18. Forest plot graphically representing the association between *CCND1*/cyclin D1 alterations and types of melanoma (nodular vs. SSM/LMM/AM) in Cutaneous Melanomas by geographic area; Figure S19. Forest plot graphically representing the association between *CCND1*/cyclin D1 alterations and types of melanoma (nodular vs. SSM/LMM/AM) in Cutaneous Melanomas by immunohistochemical pattern; Figure S20. Forest plot graphically representing the association between *CCND1*/cyclin D1 alterations and tisular expression (Lymph node metastasis vs. primary tissue) in Cutaneous Melanomas; Figure S21. Forest plot graphically representing the association between *CCND1*/cyclin D1 alterations and tisular expression (distance metastasis vs. primary tissue) in Cutaneous Melanomas; Figure S22. Forest plot graphically representing the association between *CCND1*/cyclin D1 alterations and tisular expression (distance metastasis vs. primary tissue) in Cutaneous Melanomas by geographic area; Figure S23. Forest plot graphically representing the association between *CCND1*/cyclin D1 alterations and tisular expression (distance metastasis vs. primary tissue) in Cutaneous Melanomas by geographic area; Figure S24. Forest plot graphically representing the frequency of *CCND1*/cyclin D1 alterations in Uveal Melanomas; Figure S25. Forest plot graphically representing the association between *CCND1*/cyclin D1 alterations and Largest Basal Dimension in Uveal Melanomas; Figure S26. Forest plot graphically representing the association between *CCND1*/cyclin D1 alterations and pathology (epithelioid vs. spindle/mixed cell shape) in Uveal Melanomas; Figure S27. Forest plot graphically representing the frequency of *CCND1*/cyclin D1 alterations in Mucosal Melanomas; Figure S28. Forest plot graphically representing the frequency of *CCND1*/cyclin D1 alterations in Mucosal Melanomas by anatomical site; Figure S29. Forest plot graphically representing the association between *CCND1*/cyclin D1 alterations and Recurrence in Mucosal Melanomas; Figure S30. Forest plot graphically representing the association between *CCND1*/cyclin D1 alterations and Thickness in Mucosal Melanomas; Figure S31. Forest plot graphically representing the association between *CCND1*/cyclin D1 alterations and M status in Mucosal Melanomas; Figure S32. Forest plot graphically representing the association between *CCND1*/cyclin D1 alterations and Necrosis in Mucosal Melanomas; Figure S33. A funnel plot of estimated logHR against its standard error, graphically representing the analysis of small-study effects on Overall Survival in Cutaneous Melanomas; Figure S34. A funnel plot of estimated logOR against its standard error, graphically representing the analysis of small-study effects on Distance Metastasis vs. Primary tissue in Cutaneous Melanomas; Figure S35. A funnel plot of estimated logOR against its standard error, graphically representing the analysis of small-study effects on Type of Cutaneous Melanomas; Figure S36. A funnel plot of estimated logOR against its standard error, graphically representing the analysis of small-study effects on Ulceration in Cutaneous Melanomas; Figure S37. A funnel plot of estimated logOR against its standard error, graphically representing the analysis of small-study effects on Ulceration in Cutaneous Melanomas.
