# Supplementary material for: Prognostic and Clinicopathological Significance of CCND1/Cyclin D1 Upregulation in Melanomas: A Systematic Review and Comprehensive Meta-Analysis
Source: Cancers (Basel). 2021 Mar 15;13(6):1314. doi: 10.3390/cancers13061314 (PMC7999631; doi:10.3390/cancers13061314)
Supplement: Supplementary file 1 [file cancers-13-01314-s001.pdf]

Appendix to the manuscript

Prognostic and clinicopathological significance of CCND1/cyclin D1 upregulation in melanomas: a systematic review and comprehensive meta-analysis.

Lucía González-Ruiz [1], Miguel Ángel González-Moles [2, 3, 4 *], Isabel González-Ruiz [2,3], Isabel Ruiz-Ávila [3,5], and Pablo Ramos-García [2, 3]

1 - Dermatology Service, Ciudad Real General University Hospital, Ciudad Real, Spain.

2 - School of Dentistry, University of Granada, Granada, Spain

3 - Instituto de Investigación Biosanitaria ibs.GRANADA, Granada, Spain

4 - WHO Collaborating Group for Oral Cancer

5 - Pathology Service, San Cecilio Hospital Complex, Granada, Spain.

Corresponding Author:

*Miguel Ángel González Moles

Full Professor, Oral Medicine Department, School of Dentistry, University of Granada, Granada, Paseo de Cartuja s/n, 18071 Granada, Spain.

Tel.:+34958243804; fax:+34958240908.E-mail: magonzal@ugr.es

Table of contents

1. Search strategy. Table S1. 5

2. Characteristics of analyzed studies. Table S2 6

3. Meta-analysis on the frequency of CCND1/cyclin D1 alterations in Cutaneous Melanoma 13

3.1 *CCND1*/cyclin D1 alterations in Nodular Melanoma 13

3.1 *CCND1*/cyclin D1 alterations in Superficial Spreading Melanoma 14

3.1 *CCND1*/cyclin D1 alterations in Lentigo Malignant Melanoma 15

3.1 *CCND1*/cyclin D1 alterations in Acral melanoma....16

4. Meta-analysis on the association between *CCND1*/cyclin D1 alterations and Overall Survival in Cutaneous Melanoma 17

4.1 *CCND1*/cyclin D1 and overall survival by alteration 17

4.2 *CCND1*/cyclin D1 and overall survival by geographic area....18

4.3 *CCND1*/cyclin D1 and overall survival by immunohistochemical pattern......19

5. Meta-analysis on the association between *CCND1*/cyclin D1 alterations and Disease-Free Survival in Cutaneous Melanoma 20

6. Meta-analysis on the association between *CCND1*/cyclin D1 alterations and Breslow Thickness in Cutaneous Melanoma....21

6.1 *CCND1*/cyclin D1 and Breslow Thickness by geographic area 21

6.2 *CCND1*/cyclin D1 and Breslow Thickness by immunohistochemical pattern 22

7. Meta-analysis on the association between *CCND1*/cyclin D1 alterations and Ulceration in Cutaneous Melanoma 23

8. Meta-analysis on the association between *CCND1*/cyclin D1 alterations and N status in Cutaneous Melanoma 24

9. Meta-analysis on the association between *CCND1*/cyclin D1 alterations and M status in Cutaneous Melanoma 25

10. Meta-analysis on the association between *CCND1*/cyclin D1 alterations and Clinical Stage in Cutaneous Melanoma 26

11. Meta-analysis on the association between *CCND1*/cyclin D1 alterations and Mitotic Rate in Cutaneous Melanoma....27

12. Meta-analysis on the association between *CCND1*/cyclin D1 alterations and Clark levels in Cutaneous Melanoma....28

13. Meta-analysis on the association between *CCND1*/cyclin D1 alterations and Type of Melanoma in Cutaneous Melanoma....29

13.1 *CCND1*/cyclin D1 and Type of Melanoma by alteration....29

13.1 *CCND1*/cyclin D1 and Type of Melanoma by geographic area 30

13.1 *CCND1*/cyclin D1 and Type of Melanoma by immunohistochemical pattern....31

14. Meta-analysis on the association between *CCND1*/cyclin D1 alterations and Lymph node metastasis vs. primary tissue in Cutaneous Melanoma....32

15. Meta-analysis on the association between *CCND1*/cyclin D1 alterations and Distance metastasis vs. Primary tissue in Cutaneous Melanoma....33

15.1 *CCND1*/cyclin D1 and Distance metastasis vs. Primary tissue by alteration 33

15.1 *CCND1*/cyclin D1 and Distance metastasis vs. Primary tissue by geographic area....34

15.1 *CCND1*/cyclin D1 and Distance metastasis vs. Primary tissue by immunohistochemical pattern....35

16. Meta-analysis on the frequency of CCND1/cyclin D1 alterations in uveal Melanoma....36

17. Meta-analysis on the association between *CCND1*/cyclin D1 alterations and Largest Basal Dimension in Uveal Melanoma....37

18. Meta-analysis on the association between *CCND1*/cyclin D1 alterations and Pathology in Uveal Melanoma....38

19. Meta-analysis on the frequency of CCND1/cyclin D1 alterations in Mucosal Melanoma....39

19.1 *CCND1*/cyclin D1 in Mucosal Melanomas by alteration....39

19.2 *CCND1*/cyclin D1 in Mucosal Melanomas by anatomical site....40

20. Meta-analysis on the association between *CCND1*/cyclin D1 alterations and Recurrence in Mucosal Melanoma....41

21. Meta-analysis on the association between *CCND1*/cyclin D1 alterations and Thickness in Mucosal Melanoma....42

22. Meta-analysis on the association between *CCND1*/cyclin D1 alterations and M status in Mucosal Melanoma....43

23. Meta-analysis on the association between *CCND1*/cyclin D1 alterations and Necrosis in Mucosal Melanoma....44

24. Analysis of small-study effects....45

24.1 *CCND1*/cyclin D1 alterations and Overall Survival in Cutaneous Melanomas....45

24.2 *CCND1*/cyclin D1 alterations and Distance Metastasis vs. Primary tissue in Cutaneous Melanomas....46

24.3 *CCND1*/cyclin D1 alterations and Type of Cutaneous Melanomas....47

24.4 *CCND1*/cyclin D1 alterations and Ulceration in Cutaneous Melanomas....48

24.5 *CCND1*/cyclin D1 alterations and Clark levels in Cutaneous Melanomas....49

25 Sensitivity analysis (leave-one-out method)....50
25.1 CCND1/cyclin D1 and overall survival in cutaneous melanoma 50
25.2 CCND1/cyclin D1 and Breslow Thickness in cutaneous melanoma....51
25.3 CCND1/cyclin D1 and Ulceration in cutaneous melanoma 52
25.4 CCND1/cyclin D1 and Clark in cutaneous melanoma 53
25.5 CCND1/cyclin D1 and Type of cutaneous melanoma....54
25.6 CCND1/cyclin D1 and Distance metastasis vs primary tissue in cutaneous melanoma 55
26. List of included studies in this systematic review and meta-analysis... 56
27. Protocol....60

1. Search strategy

Table S1. Search strategy for each database, number of results, and execution date.

Database	Query	Results	Upper limit
PubMed	("cyclin d1"[MeSH Terms] OR ("cyclin"[All Fields] AND "d1"[All Fields]) OR "cyclin d1"[All Fields] OR "cyclind1"[All Fields] OR “ccnd1”[All Fields] OR “ccnd 1”[All Fields]) AND ("melanoma"[MeSH Terms] OR "melanoma"[All Fields])	492	September 2019
Embase	('cyclin d1'/exp OR 'cyclin d1' OR 'cyclind1' OR 'ccnd1' OR 'ccnd 1') AND ('melanoma'/exp OR 'melanoma')	1,110	September 2019
Web of Science	TS=(cyclin d1 OR cyclind1 OR ccnd1 OR ccnd 1) AND TS=(melanoma)	728	September 2019
Scopus	TITLE-ABS-KEY(("cyclin d1" OR "cyclind1" OR "ccnd1" OR "ccnd 1") AND ("melanoma"))	845	September 2019
Total			3,175

2. Table S2. Characteristics of studies (n=41).

Study	Year	Country	Alteration analyzed (sample size)	Type of melanomas (n)	Affected sites (n)	Recruitment period	Follow-up (months)	Methods	Anti-cyclin D1 antibody	IHC pattern	IHQ Cutoff point (%)	*CCND1* Cyclin D1 (+) (%)
Bales *et al.*	1999	USA	Cyclin D1 overexpression (32 melanomas)	Cutaneous:<br>NM (2)<br>SSM (4)<br>LMM (1)<br>N/A (17)<br>Metastatic (8)	N/A (32)	N/A	N/A	IHQ	N/A	Nuclear	1	82.60 (o)
Coupland *et al.*	2000	Germany	Cyclin D1 overexpression (82 melanomas)	Ocular (82)	Uvea (82) (iris excluded)	1973-1997	≤288	IHQ	P2D11F11 (monoclonal)	Nuclear	15	24.39 (o)
Flørenes *et al.*	2000	Norway	Cyclin D1 overexpression (245 melanomas)	Cutaneous:<br>SSM (110)<br>NM (62)<br>Metastatic (73)	N/A (245)	N/A	≤180	IHQ	DCS-6 (monoclonal)	Nuclear	5	9.30 (o)
Brantley and Harbour	2000	USA	Cyclin D1 overexpression (32 melanomas)	Ocular (32)	Uvea (32)	N/A	N/A	IHQ	P2D11F11 (monoclonal)	N/A	20	34.37 (o)
Georgieva *et al.*	2001	Germany	Cyclin D1 overexpression (60 melanomas)	Cutaneous:<br>NM (7)<br>SSM (25)<br>LMM (11)<br>N/A (6)	N/A (67)	1991-1996	N/A	IHQ	HD11 (monoclonal)	N/A	5	51.67 (o)
Sauter *et al.*	2002	USA	*CCND1* amplification (102 melanomas)<br>Cyclin D1 overexpression (102 melanomas)	Cutaneous:<br>NM (17)<br>SSM (57)<br>LMM (18)<br>ALM (10)	N/A (102)	N/A	N/A	FISH<br>IHQ	ASM29 (monoclonal)	Nuclear	10	11.76 (a)<br>30.39 (o)

Rosenwald *et al.*	2003	USA	Cyclin D1 overexpression (7 melanomas)	Cutaneous: NM (4) SSM (3)	N/A (7)	N/A	N/A	IHQ	N/A (monoclonal)	Nuclear	Intensity-based	42.86 (o)
Errico *et al.*	2003	Italy	Cyclin D1 overexpression (45 melanomas)	Uveal (45)	Choroid (45)	1998-1996	3-103	IHQ	N/A	Nuclear	N/A	51.11 (o)
Alonso *et al.*	2004	Spain	Cyclin D1 overexpression (155 melanomas)	Cutaneous: Primary (90) Metastatic (65)	N/A	1995-2000	≤72	IHQ	DCS-6 (monoclonal)	Nuclear	30	23.33 (o)
Bachmann *et al.*	2004	Norway	Cyclin D1 overexpression (187 melanomas)	Cutaneous: Nodular (130) Metastatic (57)	N/A	1981-1997	≤120	IHQ	RM-9104-SO (monoclonal)	Nuclear	Staining index≥4	61.50 (o)
Utikal *et al.*	2005	Germany	Cyclin D1 overexpression (lack of essential data, not meta-analyzed) *CCND1* amplification (31 melanomas)	Cutaneous: NM (13) SSM (4) LMM (1) ALM (1) Metastatic (12)	N/A	N/A	N/A	IHQ FISH	DCS-6 (monoclonal)	N/A	N/A	40.0 (a)
Ramirez *et al.*	2005	USA	Cyclin D1 overexpression (87 melanomas)	Cutaneous: NM (13) SSM (15) LMM (2) In situ (28) Metastatic (29)	N/A	N/A	N/A	IHQ	DCS-6 (monoclonal)	Nuclear	1	41.38 (o)
Takata *et al.*	2005	Japan	*CCND1* amplification (21 melanomas)	Cutaneous: ALM (10) Metastatic (11)	Sole (13) Toe (3) Finger nail (3) Toe nail (1) Palm (1)	N/A	N/A	FISH	N/A	N/A	N/A	23.80 (a)

Curtin *et al.*	2005	USA<br>Japan<br>South Korea<br>Germany	*CCND1* amplification (lack of essential data, not meta-analyzed)<br>Cyclin D1 overexpression (39 melanomas)	Cutaneous:<br>NM (2)<br>SSM (17)<br>LMM (11)<br>ALM (3)<br>N/A (6)	Trunk (13)<br>Head (10)<br>Upper extremity (6)<br>Lower extremity (6)<br>Sole (2)<br>Subungueal(1)	N/A	N/A	IHQ	ASM29 (monoclonal)	N/A	1	25.64 (o)
Glatz-Krieger *et al.*	2006	Switzerland	*CCND1* amplification (326 melanomas)	Cutaneous:<br>NM (39)<br>SSM (70)<br>LMM (4)<br>ALM (12)<br>Mucosa(10)<br>Uvea (83)<br>Metastatic (108) (from cutaneous melanomas)	N/A	N/A	N/A	FISH	N/A	N/A	N/A	5.21 (a)
Stefanaki *et al.*	2007	Greece	Cyclin D1 overexpression (16 melanomas)	Cutaneous:<br>NM (5)<br>SSM (9)<br>Satellite (2)	Trunk (2)<br>Head (7)<br>Upper extremity (2)<br>Lower extremity (5)	N/A	N/A	IHQ	DCS-6 (monoclonal)	Nuclear	10	56.25 (o)
Cassarino *et al.*	2008	USA	Cyclin D1 overexpression (37 melanomas)	Cutaneous:<br>NM (11)<br>N/A (13)<br>Metastatic (13)	N/A	N/A	≤94	IHQ	N/A	Nuclear	N/A	24.32 (o)

Morey *et al.*	2009	Australia	*CCND1* amplification (20 melanomas)	Cutaneous: NM (3) SSM (6) N/A (1) Metastatic (10)	Primary: Trunk (1) Head (2) Upper extremity (6) Lower extremity (1) Metastatic: Brain (7) Lung (1) Oral cavity (1) Upper extremity (1)	N/A	N/A	FISH	N/A	N/A	N/A	75.0 (a)
Lázár *et al.*	2009	Hungary	*CCND1* amplification (74 melanomas)	Cutaneous: NM (26) SSM (42) metastatic (6)	Chronically sun-exposed sites (15) Intermittently sun-exposed site (53)	N/A	≥36 (56 cases)	Q-PCR FISH	N/A	N/A	N/A	32.35 (a)
Gerami *et al.*	2009	USA	*CCND1* amplification (10 melanomas)	Cutaneous: Primary (10)	Head and neck (3) Trunk (1) Upper extremities (2) Lower extremities (4)	N/A	<60	FISH	N/A	N/A	N/A	30.0 (a)
Busam *et al.*	2010	USA	*CCND1* amplification (6 melanomas)	Mucosal (6)	Conjuntival (6)	N/A	N/A	FISH	N/A	N/A	N/A	66.67 (a)

Nai *et al.*	2010	Brazil	Cyclin D1 overexpression (62 melanomas; lack of essential data, not meta-analyzed)<br>*CCND1* amplification (62 melanomas)	Cutaneous:<br>SSM (26)<br>NM (13)<br>LMM (12)<br>ALM (10)<br>N/A (1)	N/A	N/A	N/A	IHQ<br>FISH	clone RBT14 (monoclonal)	Nuclear	25	54.83 (o)<br>8.06 (a)
Oba *et al.*	2011	Japan	Cyclin D1 overexpression (101 melanomas,)	Cutaneous:<br>ALM (49)<br>SSM (15)<br>LMM (5)<br>NM (7)<br>Mucosal (2)<br>Metastatic (23)	Extremity (58)<br>Trunk (8)<br>Head and neck (9)<br>Genital area (3)	1999-2009	40-110	IHQ	DCS-6 (monoclonal)	Nuclear and cytoplasmic	5	65.38 (o)
de Andrade *et al.*	2012	Brazil<br>Guatemala<br>Peru<br>Mexico	Cyclin D1 overexpression (13 melanomas)	Mucosal (13)	Oral cavity (13)	N/A	N/A	IHQ	DCS-6 (monoclonal)	Nuclear	1	100 (o)
Requena *et al.*	2012	Spain	*CCND1* amplification (8 melanomas)	Spitzoid melanomas (8)	Head and neck (1)<br>Trunk (3)<br>Upper extremities (1)<br>Upper extremities (3)	2008-2011	3-120	FISH	N/A	N/A	N/A	87.50 (a)
Hsieh *et al.*	2013	Brazil<br>Bolivia	Cyclin D1 overexpression (35 melanomas)	Mucosal (35)	Oral cavity (35)	1983-2013	N/A	IHQ	Sp4 (monoclonal)	Nuclear and cytoplasmic	30	66.67 (o)

Turri–Zanoni *et al.*	2013	Italy	Cyclin D1 overexpression (32 melanomas; lack of essential data)<br>*CCND1* amplification (17 melanomas)	Mucosal (17)	Sinonasal (17)	2003-2010	3-94	IHQ<br>FISH	N/A	N/A	25	65.62 (o)<br>5.88 (a)
Nathanson *et al.*	2013	Australia<br>USA	*CCND1* amplification (21 melanomas)	Metastastatic (21)	N/A (21)	2009-2012	≥100	CGH	N/A	N/A	N/A	52.17 (a)
Chraybi *et al.*	2013	France	*CCND1* amplification (16 melanomas)<br>Cyclin D1 overexpression (16 melanomas)	Mucosal (16)	Sinonasal (16)	1995-2012	N/A	IHQ<br>Q-PCR<br>FISH	Sp4 (monoclonal)	Nuclear	10	37.5 (a)<br>62.5 (o)
Diaz *et al.*	2014	Spain	Cyclin D1 overexpression (34 melanomas; lack of essential data, not meta-analyzed)<br>*CCND1* amplification (34 melanomas)	ALM (34)	Lower extremities (30)<br>Upper extremities (4)	2002-2008	N/A	IHQ<br>FISH	Sp4 (monoclonal)	Nuclear	N/A	23.53 (a)
Kiszner *et al.*	2014	Hungary	Cyclin D1 overexpression (83 melanomas)	Cutaneous:<br>Primary (62)<br>Metastatic (21)	N/A (83)	2003-2006	N/A	IHQ	Sp4 (monoclonal)	Nuclear	N/A	69.88 (o)
Young *et al.*	2014	Australia	Cyclin D1 overexpression (130 melanomas; lack of essential data, not meta-analyzed)<br>*CCND1* amplification (143 melanomas)	Cutaneous:<br>Primary (143)	N/A (143)	2003-2004	Median78	IHQ<br>FISH	Sp4 (monoclonal)	N/A	>10 and intensity-based	94.61 (o)<br>15.38 (a)
Romano *et al.*	2016	USA	*CCND1* amplification (7 melanomas)	ALM (3)<br>NM (2)<br>In situ (1)<br>Unknown (1)	Nail (7)	2000-2013	N/A	FISH	N/A	N/A	N/A	42.85 (a)

Donigan *et al.*	2017	USA	Cyclin D1 overexpression (13 melanomas)	Skin:<br>Primary (7)	N/A (7; missing data)	2007-2015	N/A	IHQ	N/A	N/A	Intensity-based	69.23 (o)
Kong *et al.*	2017	China	*CCND1* amplification (514 melanomas)	Cutaneous:<br>ALM (514)	N/A	2007-2015	≤96	Q-PCR	N/A	N/A	N/A	26.65 (a)
Su *et al.*	2017	China	*CCND1* amplification (44 melanomas)	Cutaneous:<br>ALM (44)	Upper extremities (6)<br>Lower extremities (38)	2010-2014	N/A	FISH	N/A	N/A	N/A	43.18 (a)
Sini *et al.*	2018	Italy	*CCND1* amplification (262 melanomas)	Cutaneous:<br>Primary (118)<br>Metastatic (144)	N/A	N/A	N/A	FISH	N/A	N/A	N/A	5.72 (a)
Haugh *et al.*	2018	USA	*CCND1* amplification (43 melanomas)	ALM (19)<br>SSM (19)<br>LMM (1)<br>NM (4)	Dorsal (21)<br>Foot (16)<br>Hand (5)	N/A	N/A	FISH	N/A	N/A	N/A	16.27 (a)
Yeh *et al.*	2019	USA	*CCND1* amplification (122 melanomas)	N/A	Acral sites hands and feet (122)	N/A	N/A	NGS	N/A	N/A	N/A	19.67 (a)
Jurmeister *et al.*	2019	Germany	Cyclin D1 overexpression (86 melanomas)	N/A	N/A	1980-2008	8-328	IHQ	Sp4 (monoclonal)	N/A	Median-based	50.0 (o)
Xu *et al.*	2019	China	*CCND1* amplification (213 melanomas)	Mucosal (213)	head and neck (94)<br>Oesophagus (19)<br>Anorectum (45)<br>Genitourinary (55)	2007-2018	Median 39.5	Q-PCR	N/A	N/A	N/A	27.69 (a)

NM, nodular melanoma; SSM, superficial spreading melanoma; LMM, lentigo malignant melanoma; ALM, acral lentiginous melanoma; IHC, immunohistochemistry; FISH, fluorescence *in situ* hybridization; Q-PCR, real-time quantitative PCR; CGH, comparative genomic hybridization; (a), *CCND1* amplification; (o) Cyclin D1 overexpression; N/A, not available.

3. Meta-analysis on the frequency of *CCND1*/cyclin D1 alterations in cutaneous melanoma

3.1 *CCND1*/cyclin D1 alterations in Nodular Melanoma

Figure S1. Forest plot graphically representing the frequency of *CCND1*/cyclin D1 alterations in Nodular Melanomas.

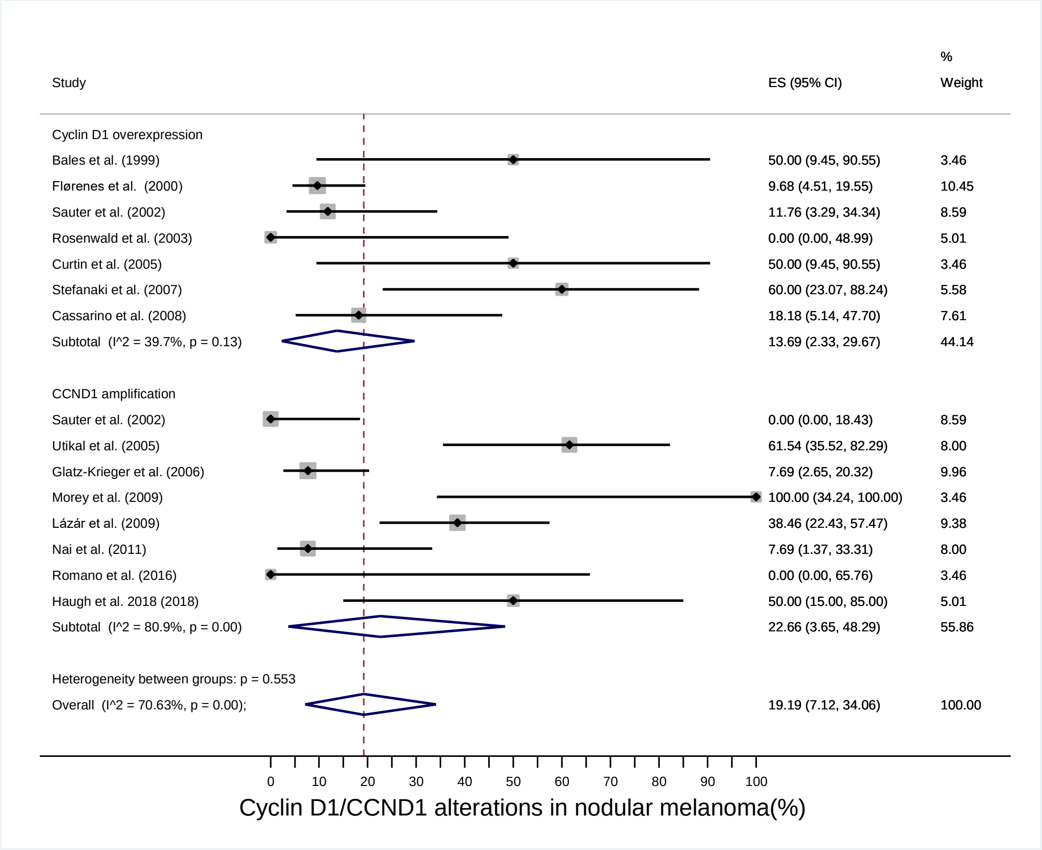

ES, estimation; CI, confidence intervals. Random-effects model (based on the DerSimonian and Laird method) using pooled proportions.

3.2 *CCND1*/cyclin D1 alterations in Superficial Spreading Melanoma

Figure S2. Forest plot graphically representing the frequency of *CCND1*/cyclin D1 alterations in Superficial Spreading Melanomas.

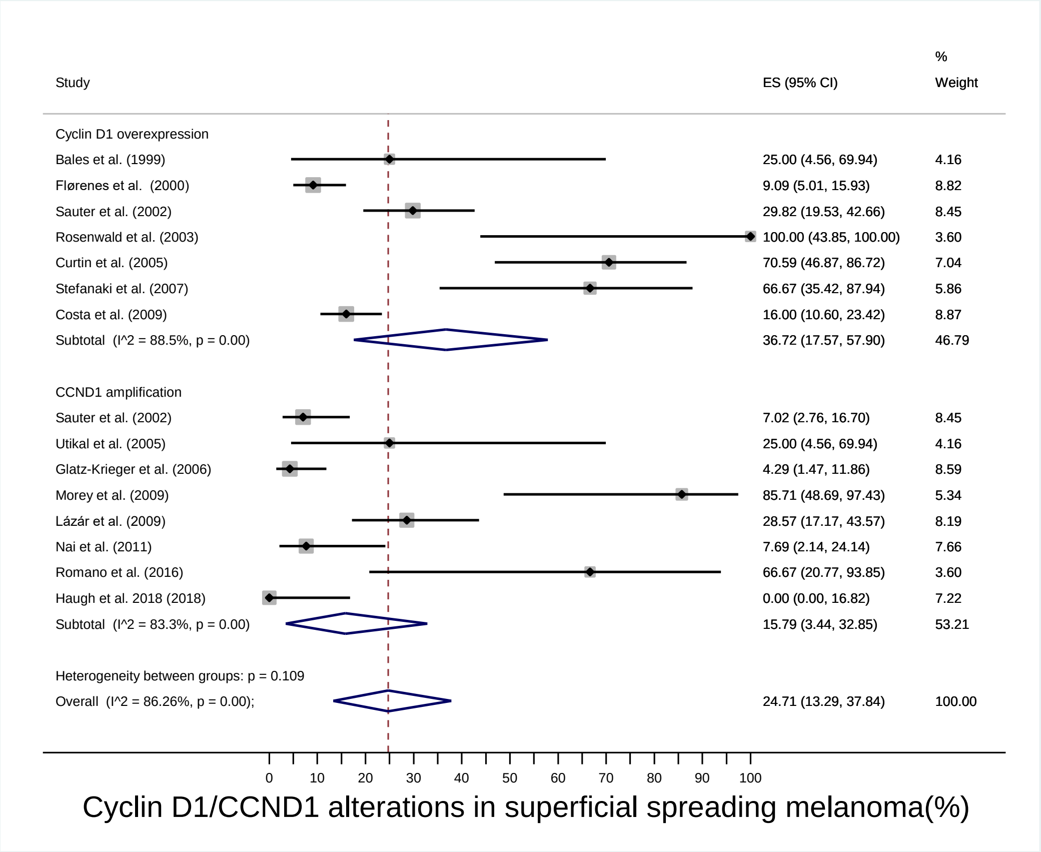

ES, estimation; CI, confidence intervals. Random-effects model (based on the DerSimonian and Laird method) using pooled proportions.

3.3 *CCND1*/cyclin D1 alterations in Lentigo Malignant Melanoma

Figure S3. Forest plot graphically representing the frequency of *CCND1*/cyclin D1 alterations in Lentigo Malignant Melanomas.

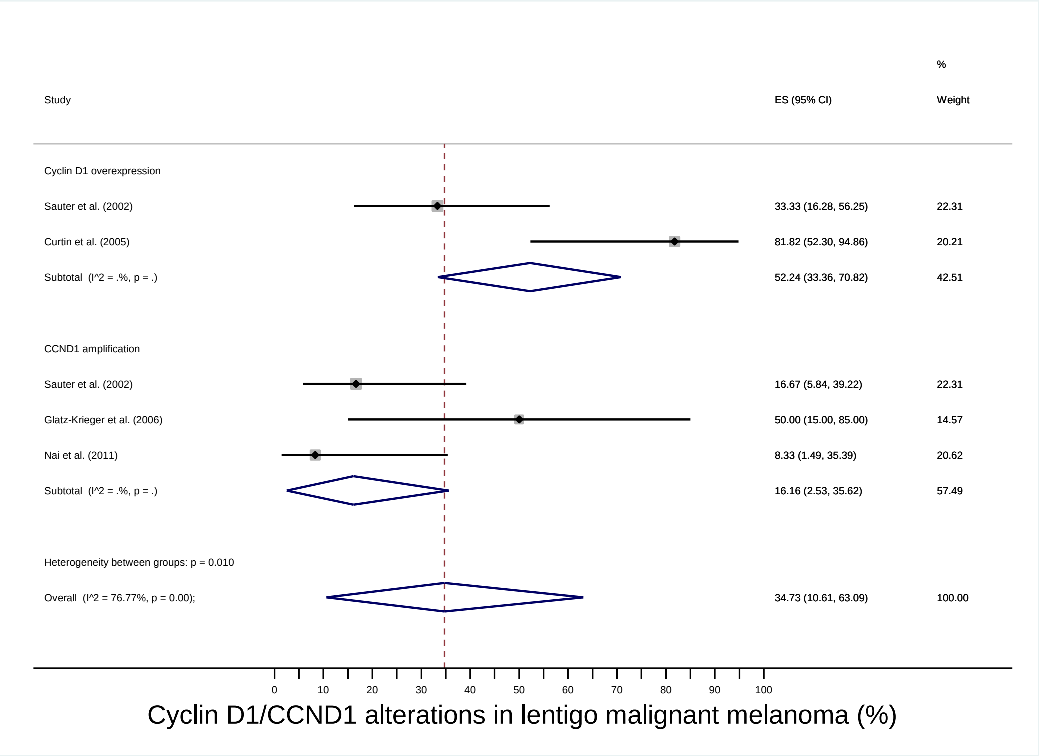

ES, estimation; CI, confidence intervals. Random-effects model (based on the DerSimonian and Laird method) using pooled proportions.

3.4 *CCND1*/cyclin D1 alterations in Acral Melanoma

Figure S4. Forest plot graphically representing the frequency of *CCND1*/cyclin D1 alterations in Acral Melanomas.

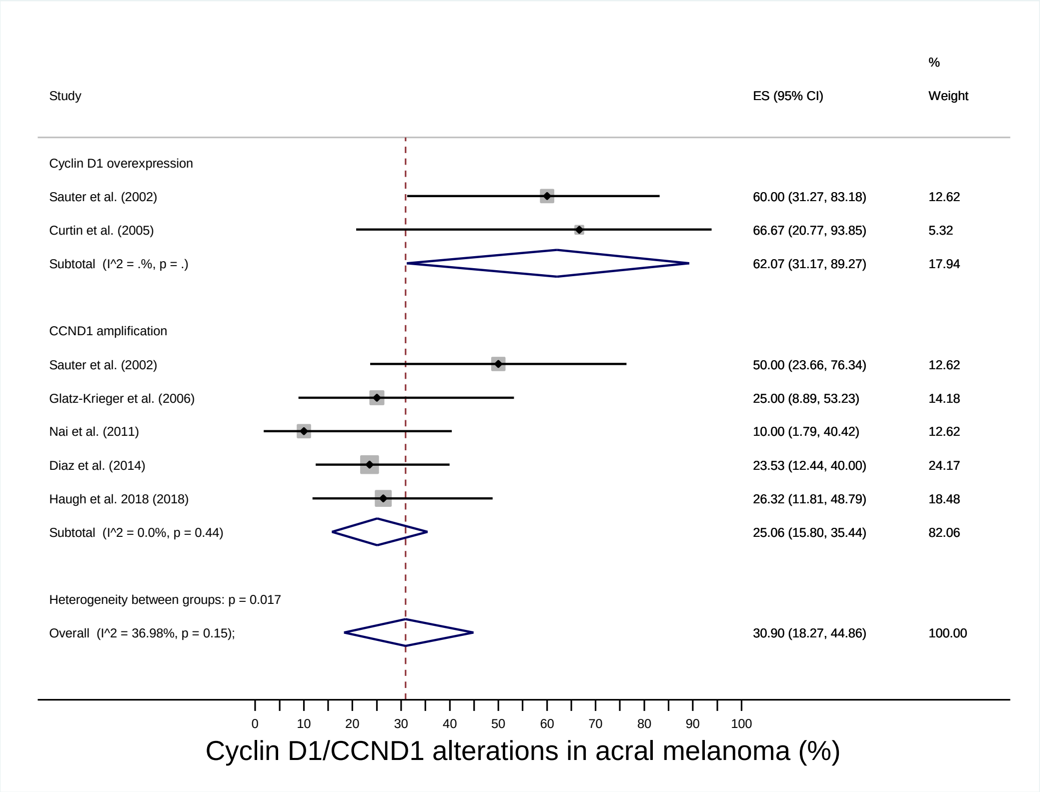

ES, estimation; CI, confidence intervals. Random-effects model (based on the DerSimonian and Laird method) using pooled proportions.

4. Meta-analysis on the association between CCND1/cyclin D1 alterations and Overall Survival in Cutaneous Melanoma

4.1 CCND1/cyclin D1 and overall survival by alteration

Figure S5. Forest plot graphically representing the association between *CCND1*/cyclin D1 alterations and Overall Survival in Cutaneous Melanomas.

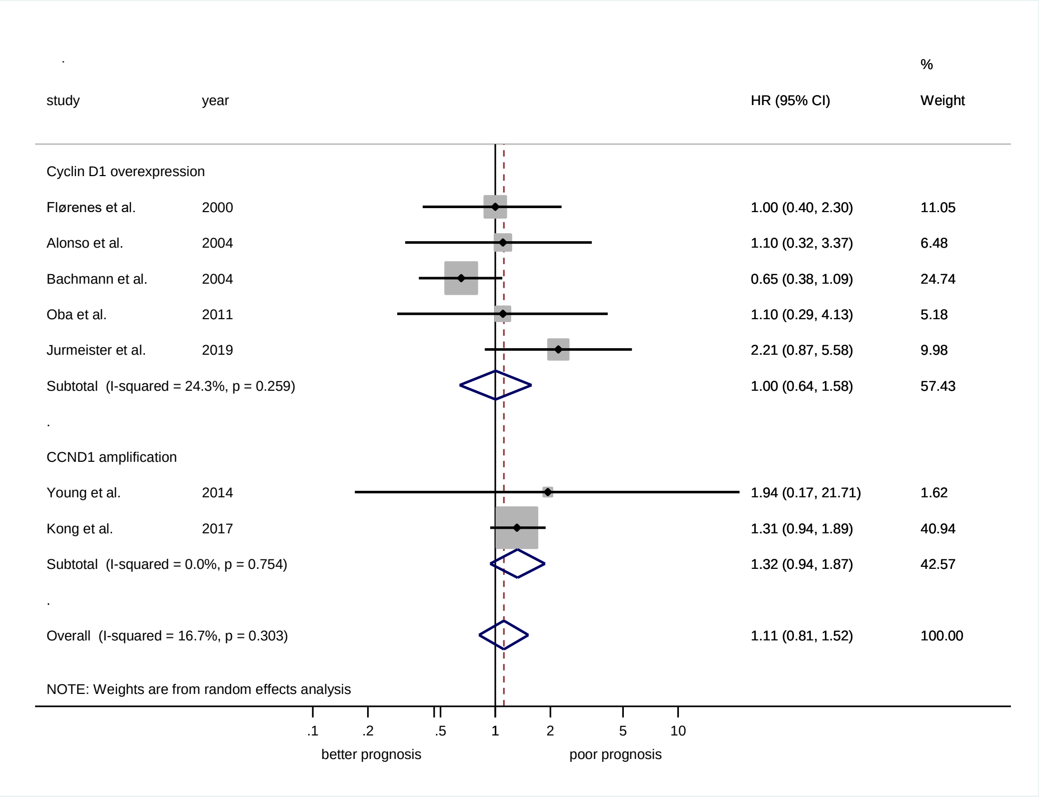

HR, hazard ratio; CI, confidence intervals. Random-effects model (based on the DerSimonian and Laird method) pooling hazard ratios.

4.2 CCND1/cyclin D1 and overall survival by geographic area

Figure S6. Forest plot graphically representing the association between *CCND1*/cyclin D1 alterations and Overall Survival in Cutaneous Melanomas by geographic area.

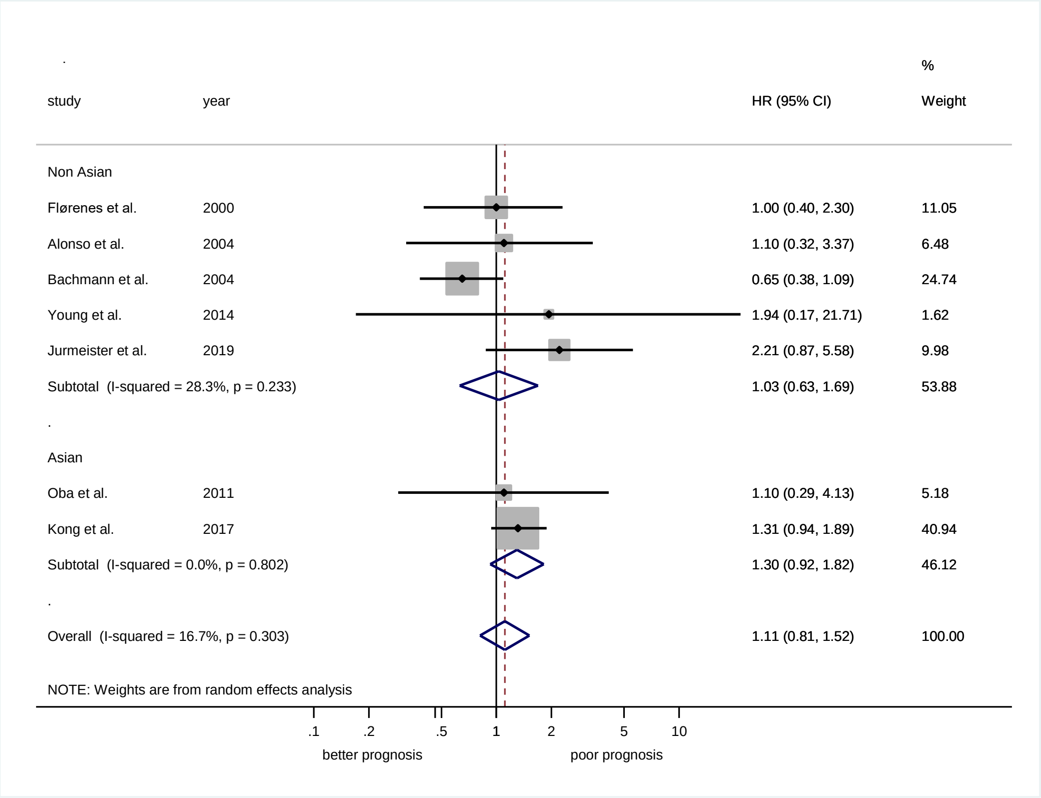

HR, hazard ratio; CI, confidence intervals. Random-effects model (based on the DerSimonian and Laird method) pooling hazard ratios.

4.3 CCND1/cyclin D1 and overall survival by immunohistochemical pattern

Figure S7. Forest plot graphically representing the association between *CCND1*/cyclin D1 alterations and Overall Survival in Cutaneous Melanomas by immunohistochemical pattern.

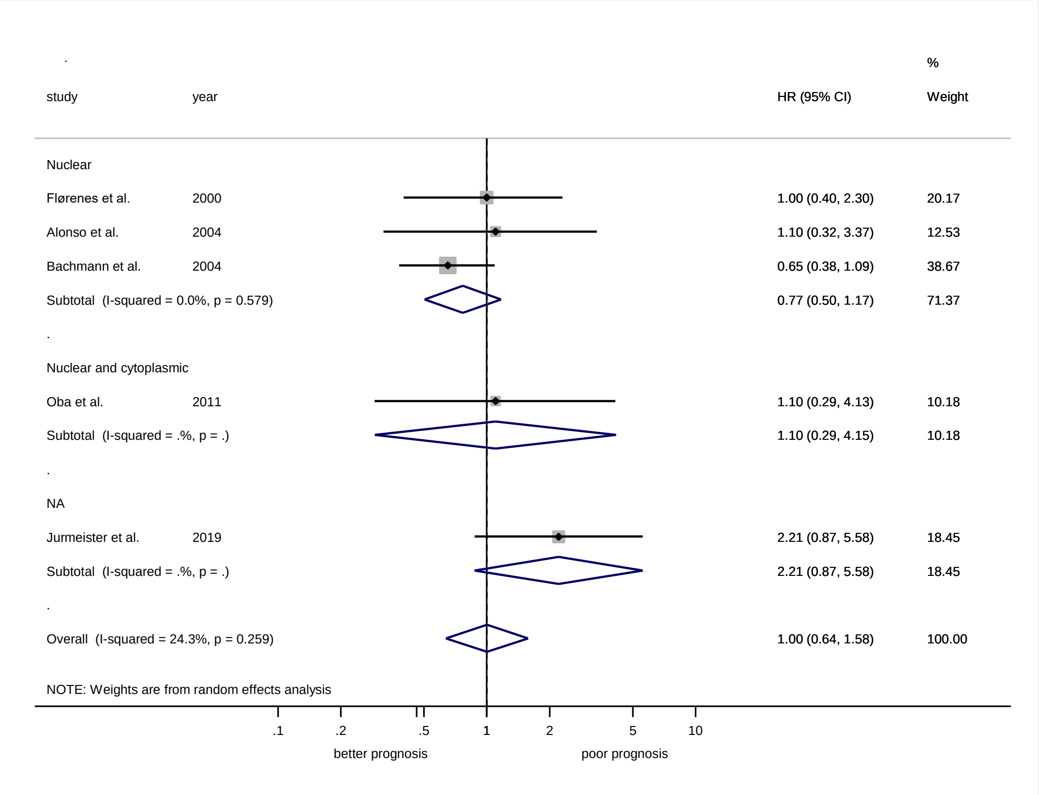

HR, hazard ratio; CI, confidence intervals. Random-effects model (based on the DerSimonian and Laird method) pooling hazard ratios.

5. Meta-analysis on the association between CCND1/cyclin D1 alterations and Disease-Free Survival in Cutaneous Melanoma

Figure S8. Forest plot graphically representing the association between *CCND1*/cyclin D1 alterations and Disease-Free Survival in Cutaneous Melanomas.

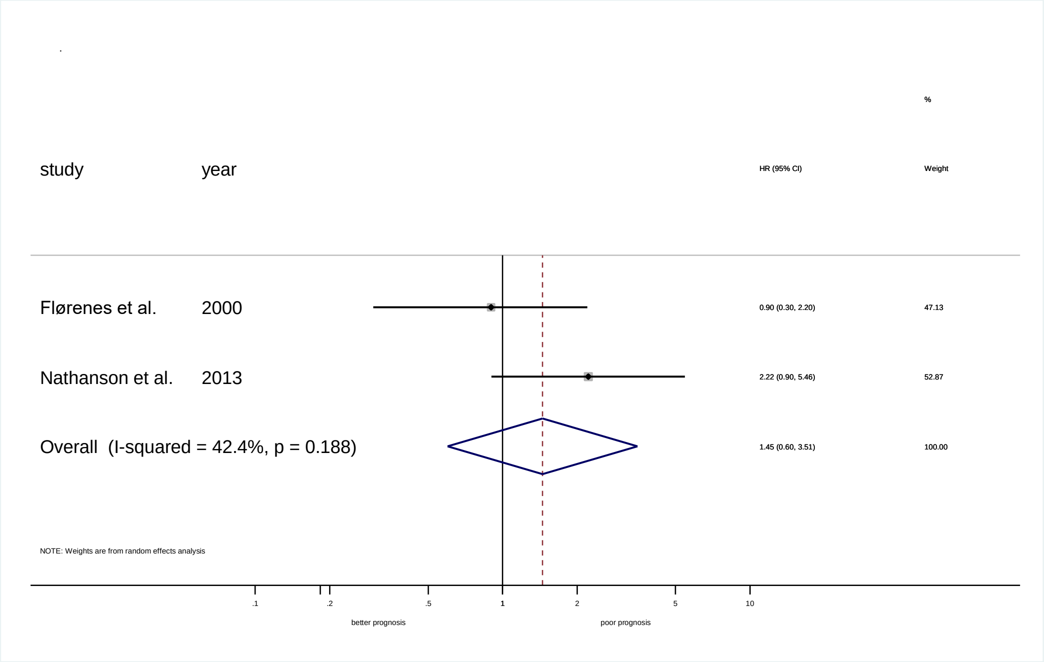

HR, hazard ratio; CI, confidence intervals. Random-effects model (based on the DerSimonian and Laird method) pooling hazard ratios.

6. Meta-analysis on the association between CCND1/cyclin D1 alterations and Breslow Thickness in Cutaneous Melanoma

6.1 CCND1/cyclin D1 and Breslow Thickness by geographic area

Figure S9. Forest plot graphically representing the association between *CCND1*/cyclin D1 alterations and Breslow Thickness in Cutaneous Melanomas by geographic area.

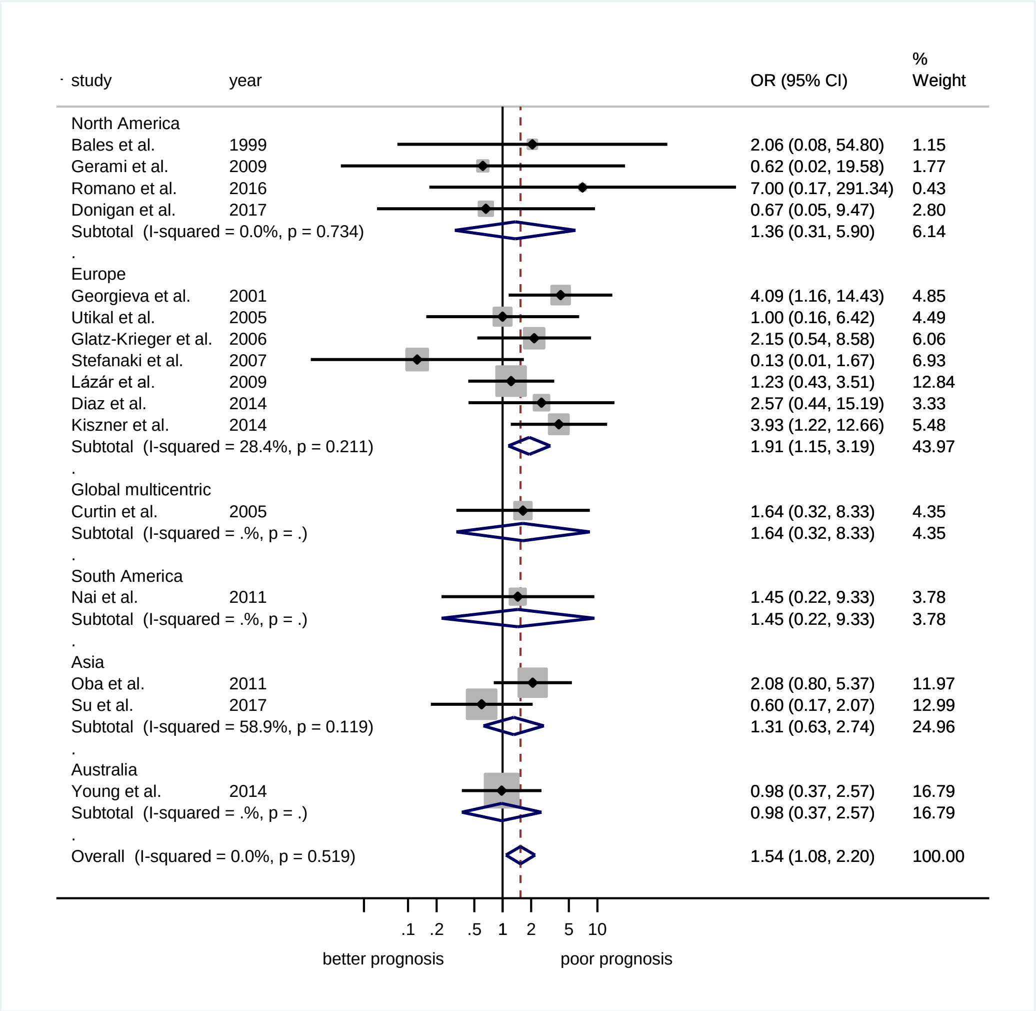

OR, odds ratio; CI, confidence intervals. Fixed-effect model (Mantel-Haenszel method) pooling odds ratios.

6.2 CCND1/cyclin D1 and Breslow Thickness by immunohistochemical pattern

Figure S10. Forest plot graphically representing the association between *CCND1*/cyclin D1 alterations and Breslow Thickness in Cutaneous Melanomas by immunohistochemical pattern.

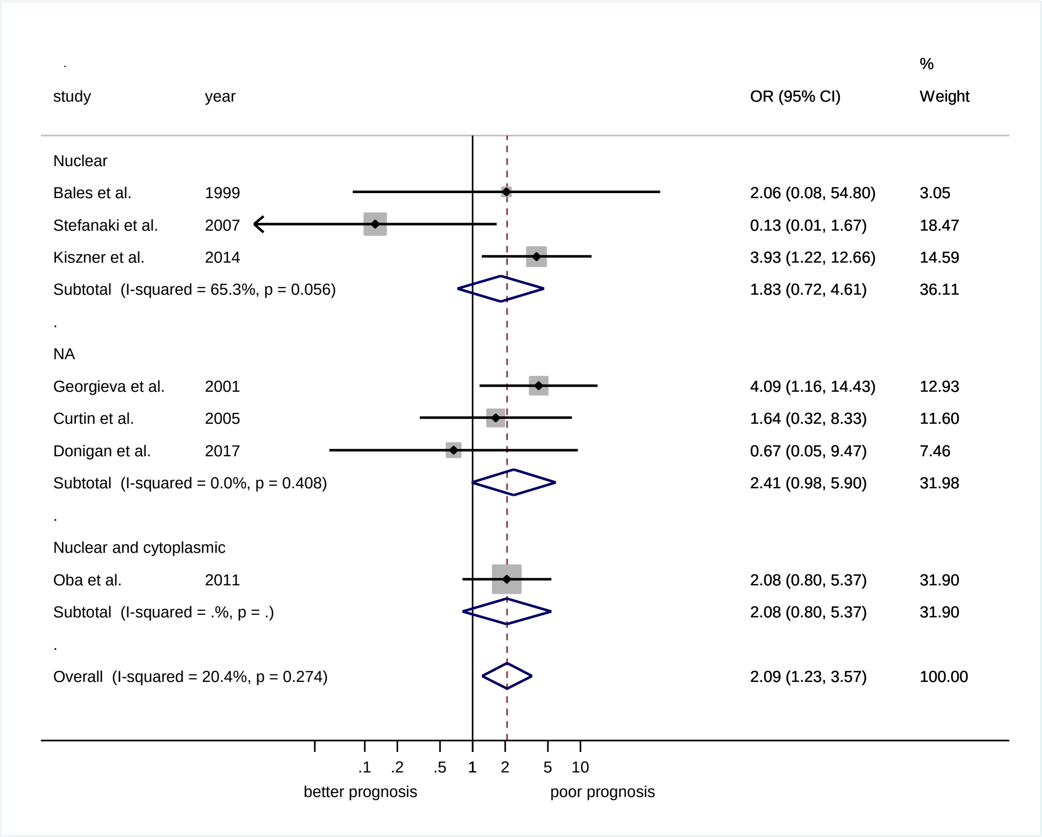

OR, odds ratio; CI, confidence intervals. Fixed-effect model (Mantel-Haenszel method) pooling odds ratios.

7. Meta-analysis on the association between CCND1/cyclin D1 alterations and Ulceration in Cutaneous Melanoma

Figure S11. Forest plot graphically representing the association between *CCND1*/cyclin D1 alterations and Ulceration in Cutaneous Melanomas.

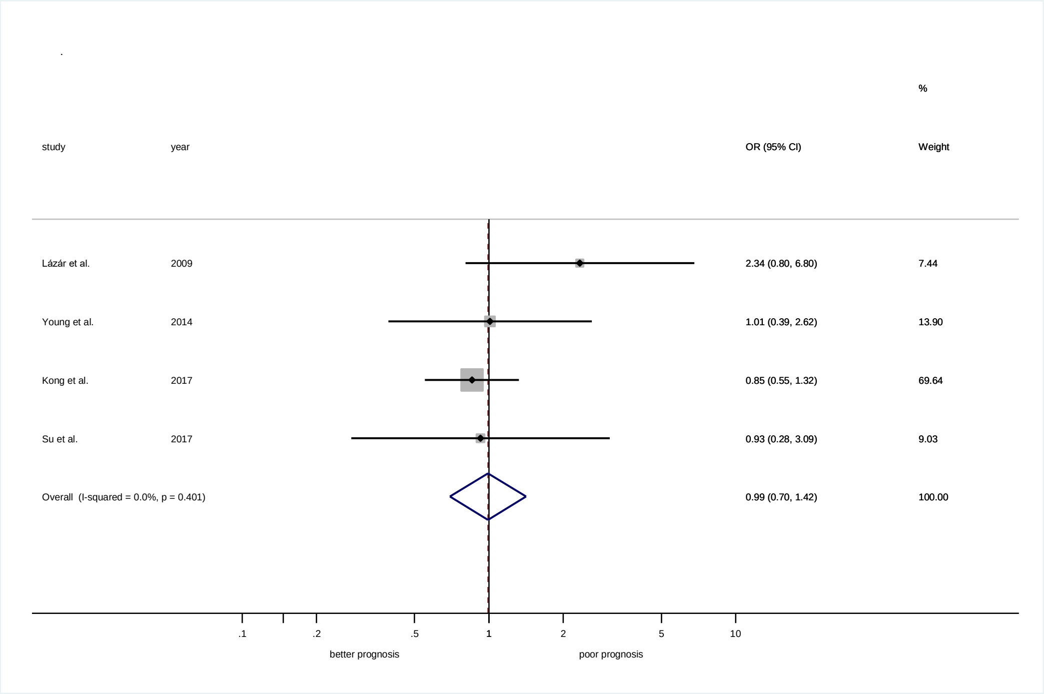

OR, odds ratio; CI, confidence intervals. Fixed-effect model (Mantel-Haenszel method) pooling odds ratios.

8. Meta-analysis on the association between CCND1/cyclin D1 alterations and N status in Cutaneous Melanoma

Figure S12. Forest plot graphically representing the association between *CCND1*/cyclin D1 alterations and N status in Cutaneous Melanomas.

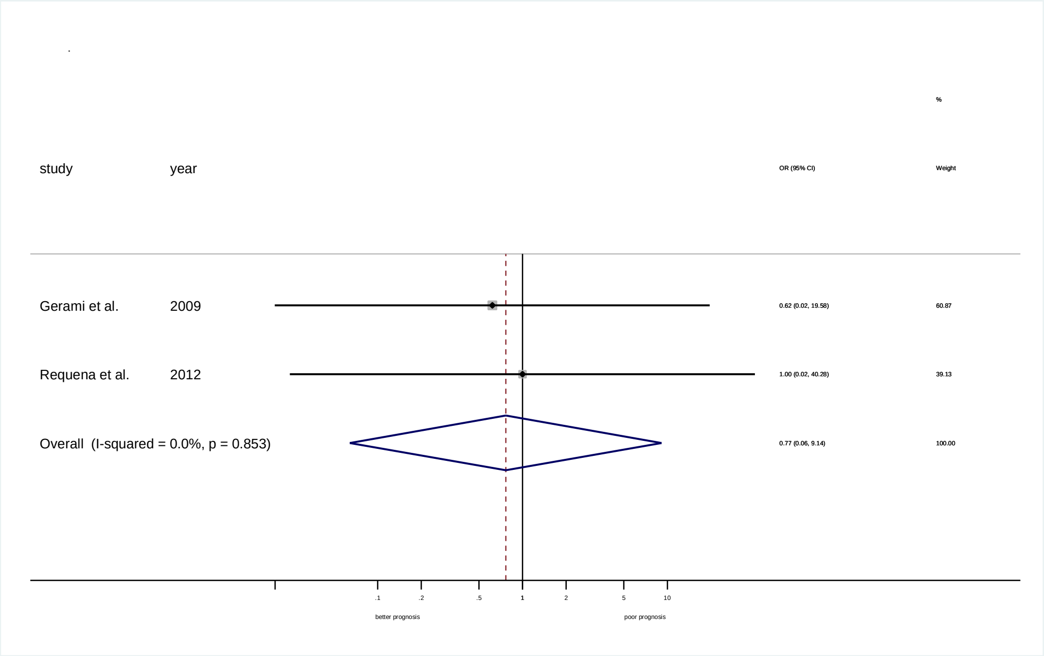

OR, odds ratio; CI, confidence intervals. Fixed-effect model (Mantel-Haenszel method) pooling odds ratios.

9. Meta-analysis on the association between CCND1/cyclin D1 alterations and M status in Cutaneous Melanoma

Figure S13. Forest plot graphically representing the association between *CCND1*/cyclin D1 alterations and M status in Cutaneous Melanomas.

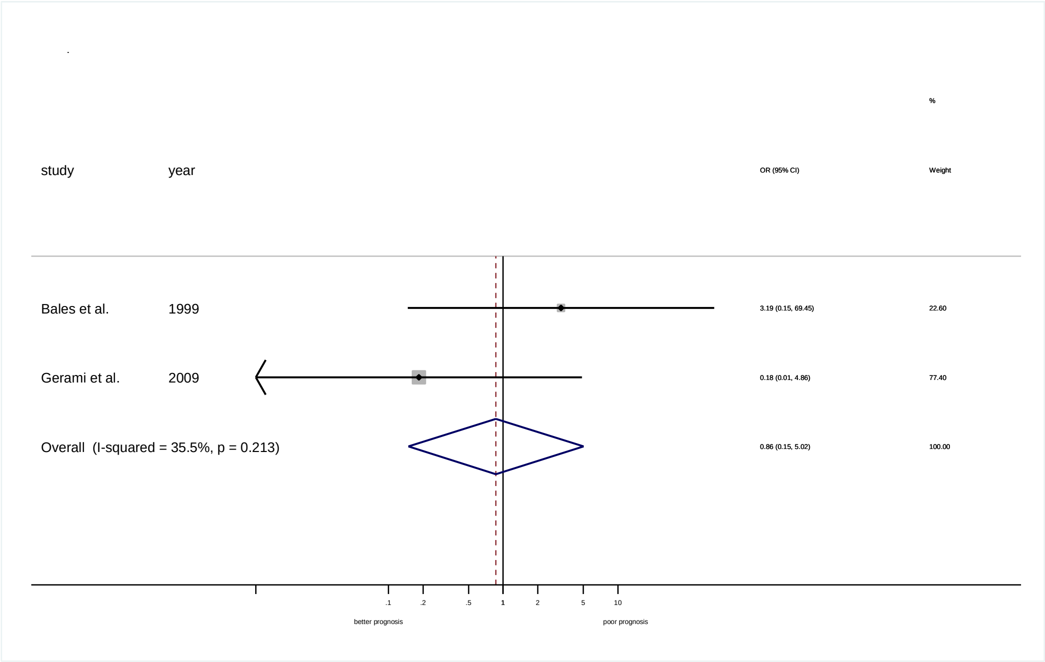

OR, odds ratio; CI, confidence intervals. Fixed-effect model (Mantel-Haenszel method) pooling odds ratios.

10. Meta-analysis on the association between CCND1/cyclin D1 alterations and Clinical Stage in Cutaneous Melanoma

Figure S14. Forest plot graphically representing the association between *CCND1*/cyclin D1 alterations and Clinical Stage in Cutaneous Melanomas.

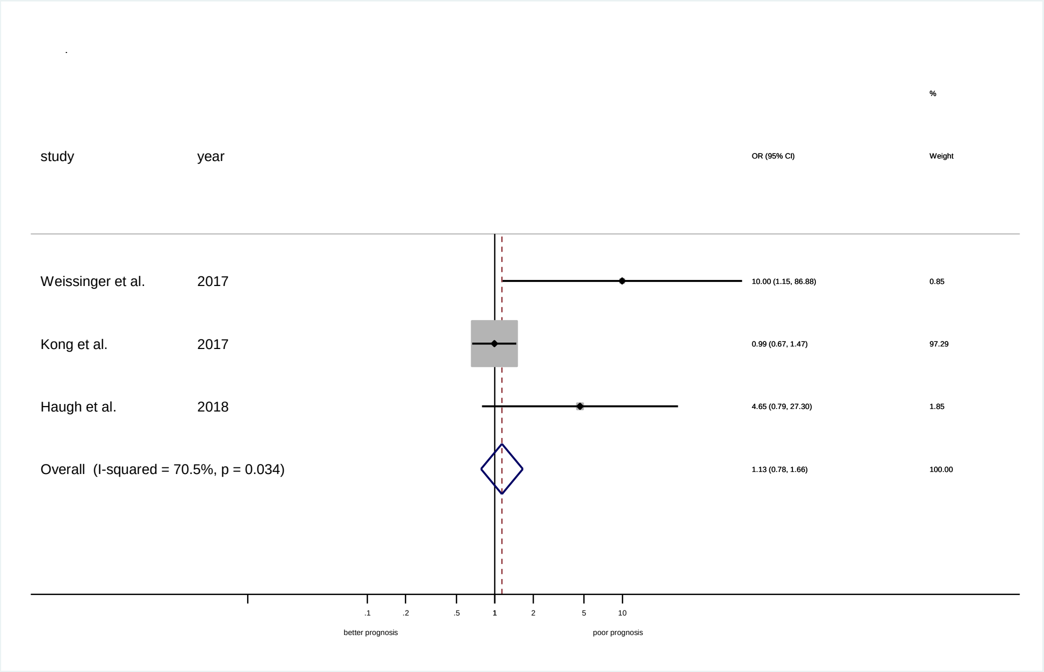

OR, odds ratio; CI, confidence intervals. Fixed-effect model (Mantel-Haenszel method) pooling odds ratios.

11. Meta-analysis on the association between CCND1/cyclin D1 alterations and Mitotic Rate in Cutaneous Melanoma

Figure S15. Forest plot graphically representing the association between *CCND1*/cyclin D1 alterations and Mitotic Rate in Cutaneous Melanomas.

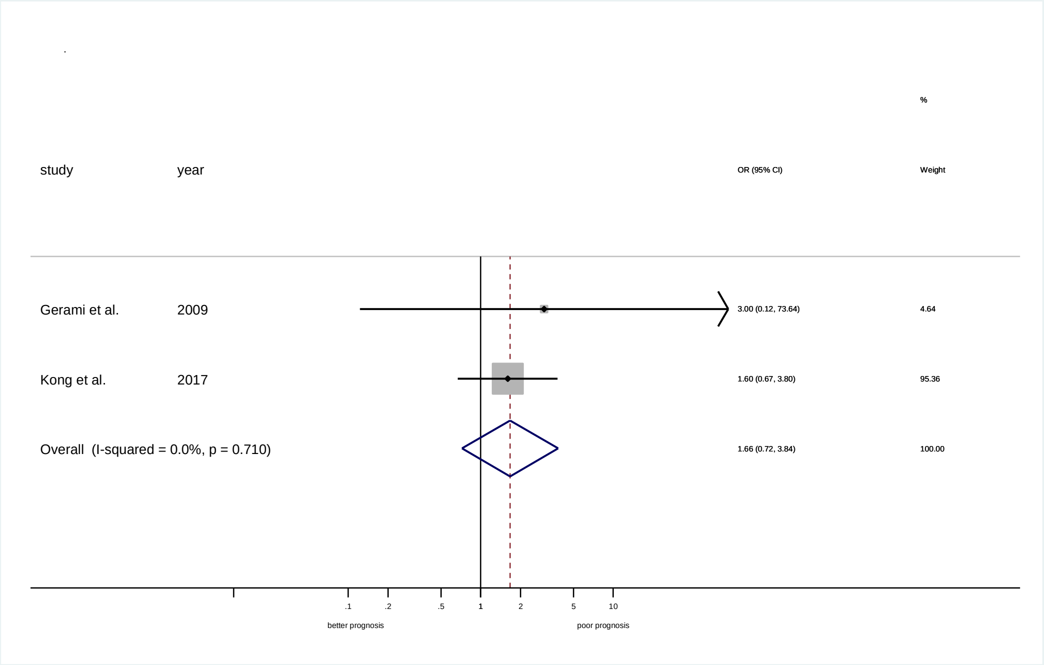

OR, odds ratio; CI, confidence intervals. Fixed-effect model (Mantel-Haenszel method) pooling odds ratios.

12. Meta-analysis on the association between CCND1/cyclin D1 alterations and Clark levels in Cutaneous Melanoma

Figure S16. Forest plot graphically representing the association between *CCND1*/cyclin D1 alterations and Clark levels in Cutaneous Melanomas.

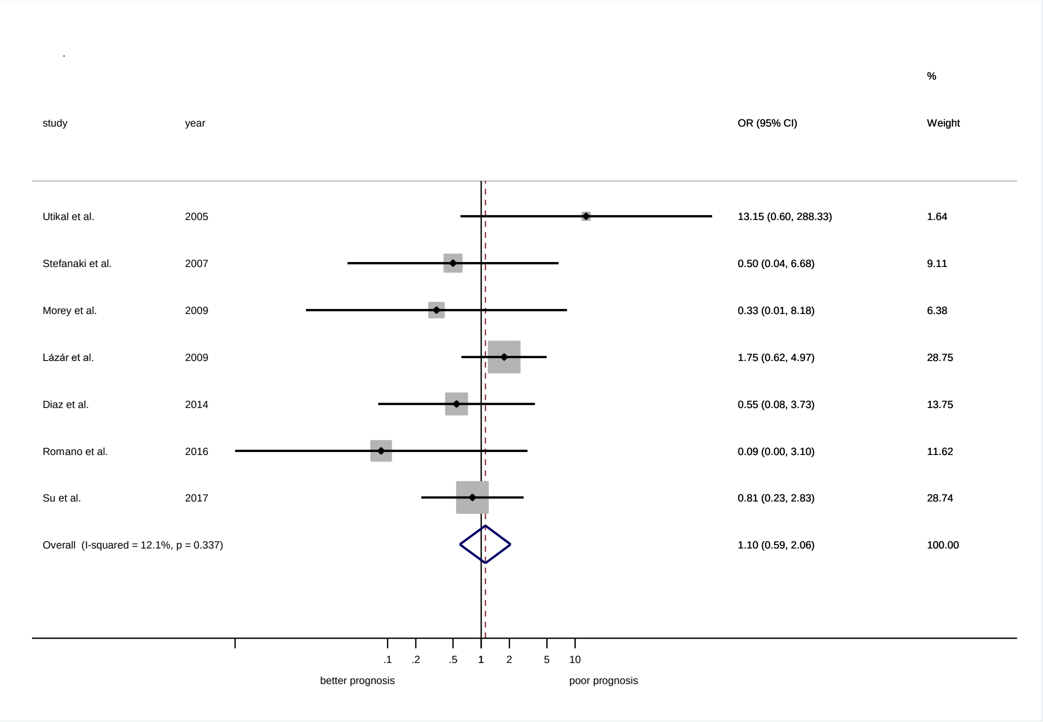

OR, odds ratio; CI, confidence intervals. Fixed-effect model (Mantel-Haenszel method) pooling odds ratios.

13. Meta-analysis on the association between CCND1/cyclin D1 alterations and Type of Melanoma in Cutaneous Melanoma

13.1 CCND1/cyclin D1 and Type of Melanoma by alteration

Figure S17. Forest plot graphically representing the association between *CCND1*/cyclin D1 alterations and types of melanoma (nodular vs. SSM/LMM/AM) in Cutaneous Melanomas.

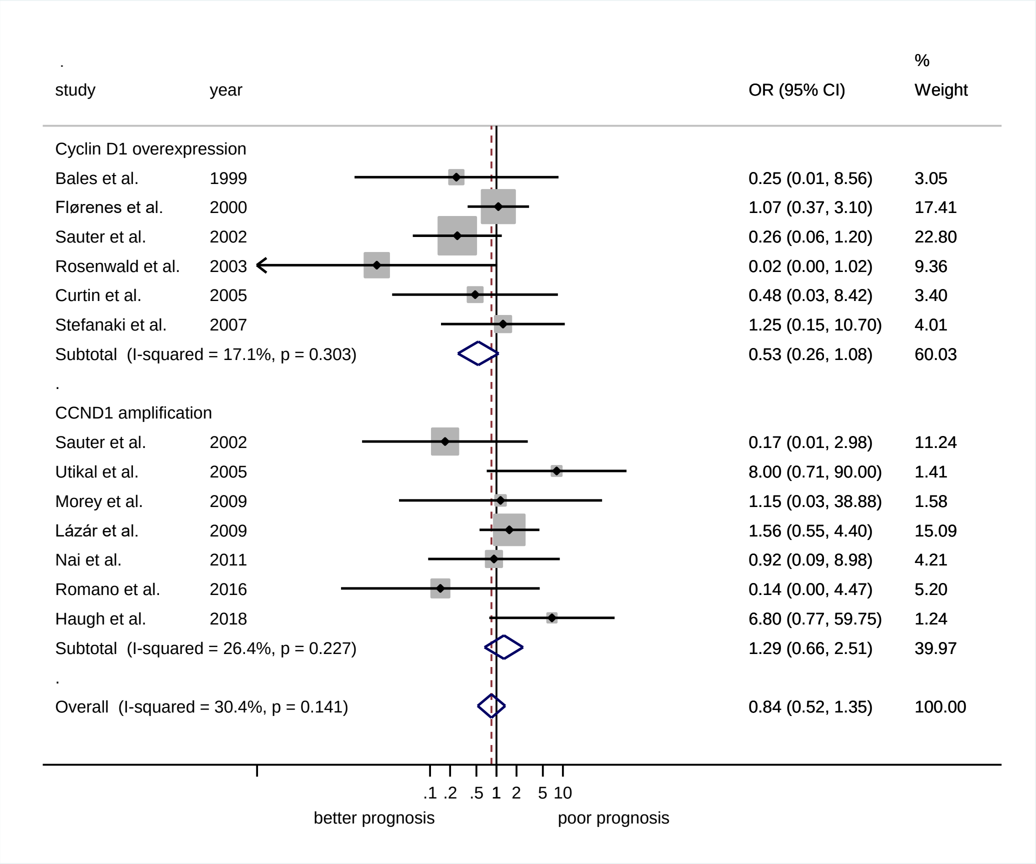

OR, odds ratio; CI, confidence intervals. Fixed-effect model (Mantel-Haenszel method) pooling odds ratios.

13.2 CCND1/cyclin D1 and Type of Melanoma by geographic area

Figure S18. Forest plot graphically representing the association between *CCND1*/cyclin D1 alterations and types of melanoma (nodular vs. SSM/LMM/AM) in Cutaneous Melanomas by geographic area.

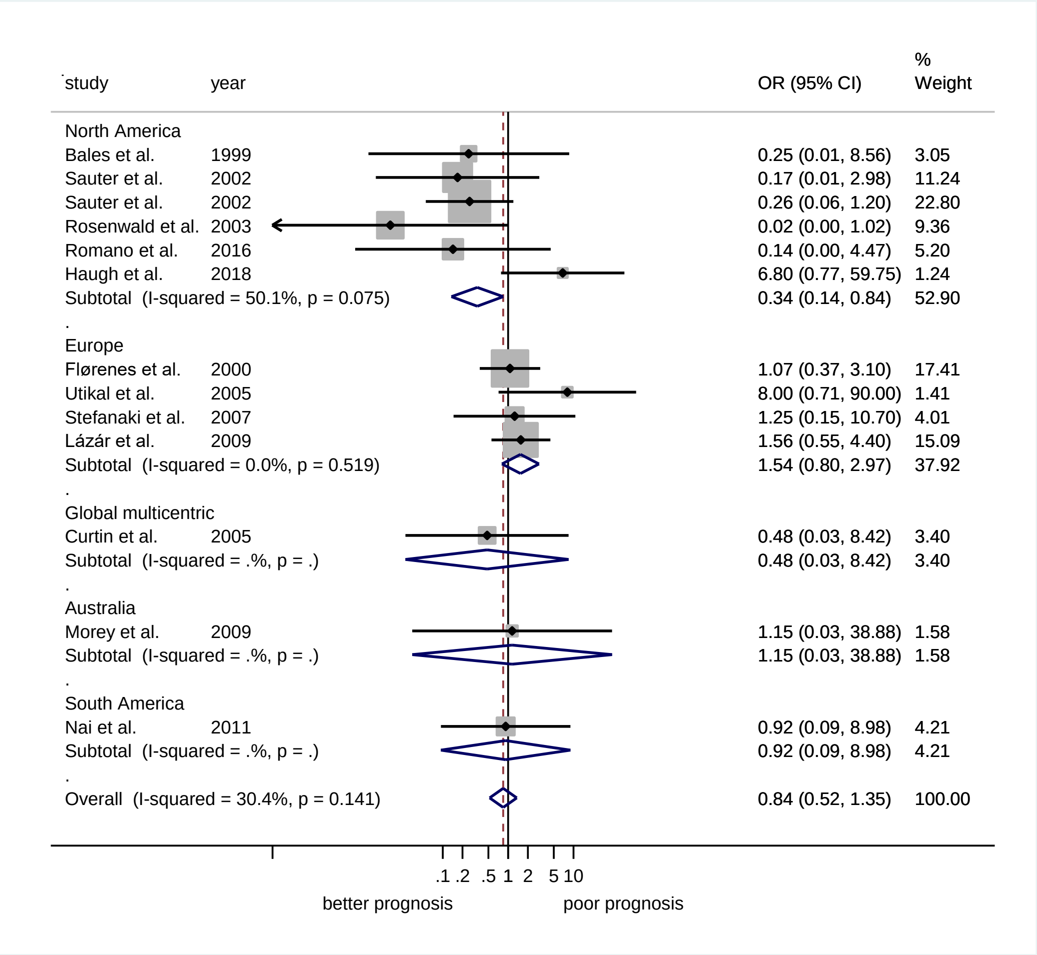

OR, odds ratio; CI, confidence intervals. Fixed-effect model (Mantel-Haenszel method) pooling odds ratios.

13.3 CCND1/cyclin D1 and Type of Melanoma by immunohistochemical pattern

Figure S19. Forest plot graphically representing the association between *CCND1*/cyclin D1 alterations and types of melanoma (nodular vs. SSM/LMM/AM) in Cutaneous Melanomas by immunohistochemical pattern.

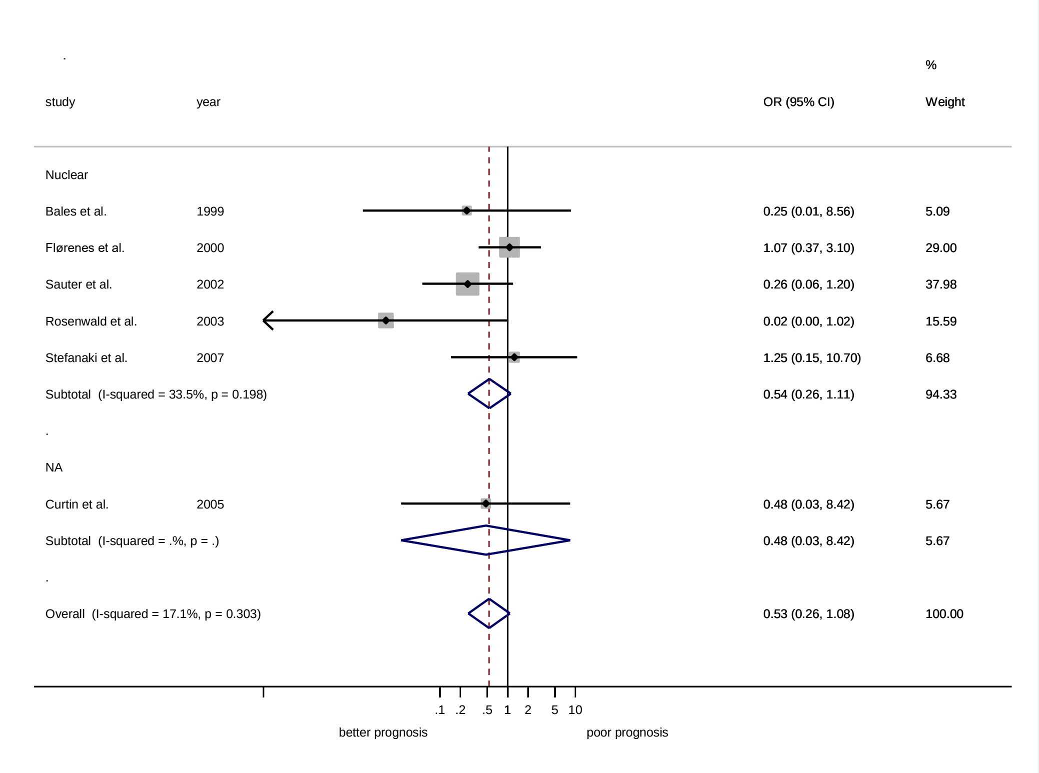

OR, odds ratio; CI, confidence intervals. Fixed-effect model (Mantel-Haenszel method) pooling odds ratios.

14. Meta-analysis on the association between CCND1/cyclin D1 alterations and Lymph node metastasis vs. primary tissue in Cutaneous Melanoma

Figure S20. Forest plot graphically representing the association between *CCND1*/cyclin D1 alterations and tisular expression (Lymph node metastasis vs. primary tissue) in Cutaneous Melanomas.

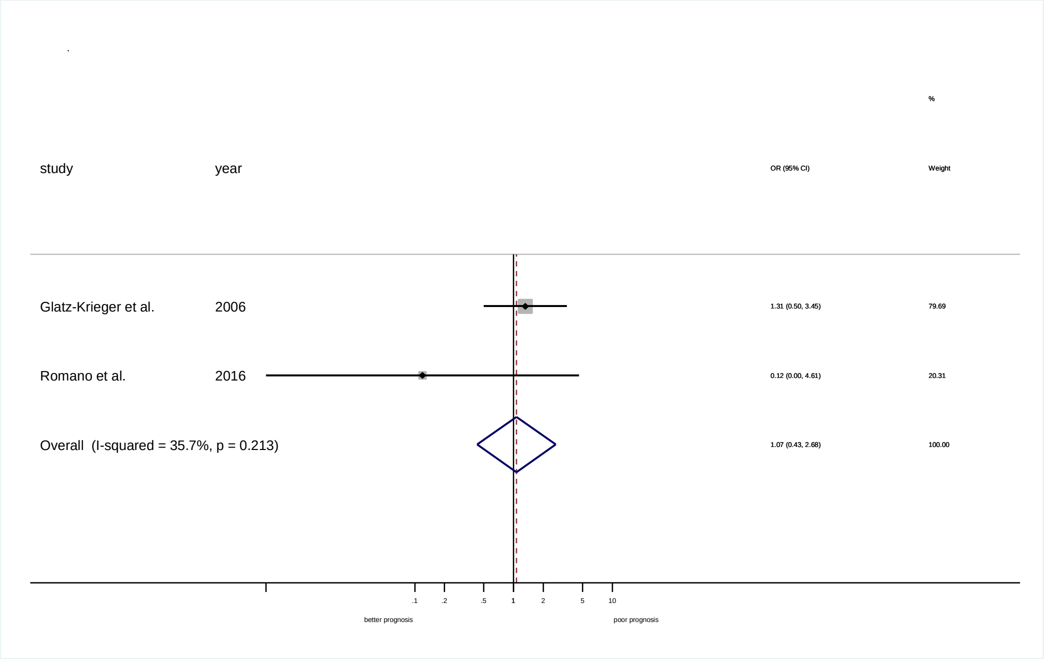

OR, odds ratio; CI, confidence intervals. Fixed-effect model (Mantel-Haenszel method) pooling odds ratios.

15. Meta-analysis on the association between CCND1/cyclin D1 alterations and Distance metastasis vs. Primary tissue in Cutaneous Melanoma

15.1 CCND1/cyclin D1 and Distance metastasis vs. Primary tissue by alteration

Figure S21. Forest plot graphically representing the association between *CCND1*/cyclin D1 alterations and tisular expression (distance metastasis vs. primary tissue) in Cutaneous Melanomas.

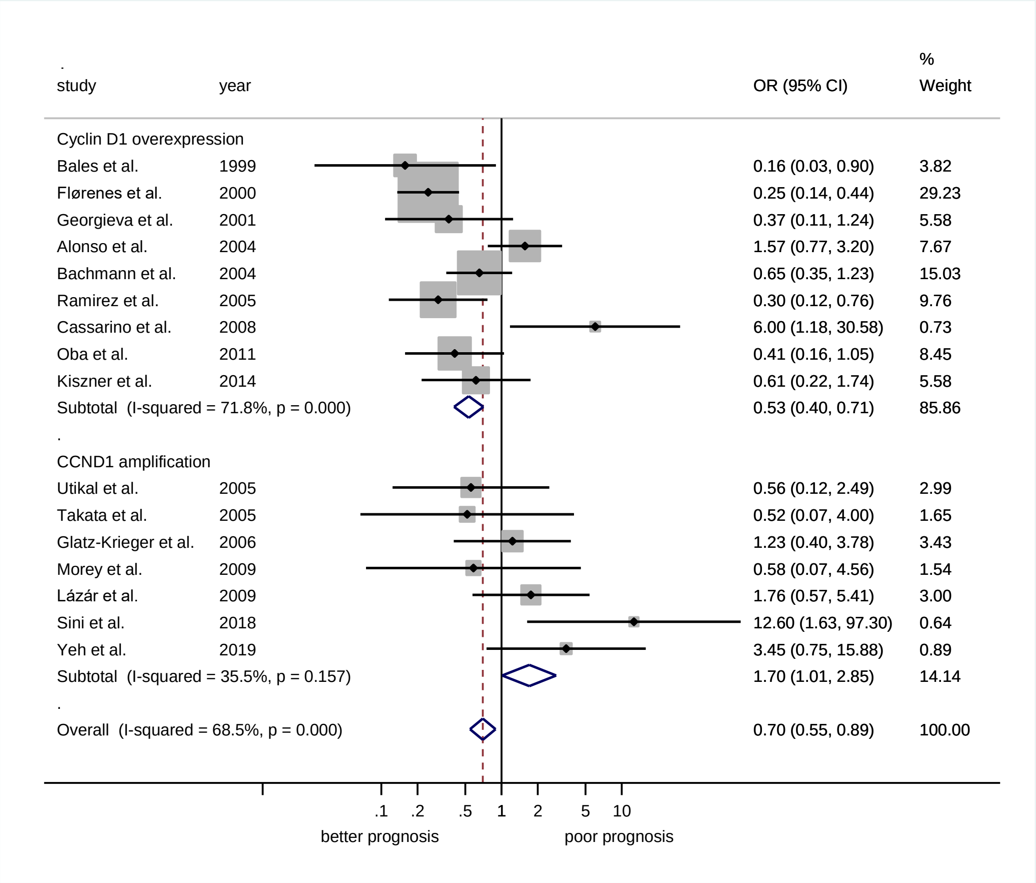

OR, odds ratio; CI, confidence intervals. Fixed-effect model (Mantel-Haenszel method) pooling odds ratios.

15.2 CCND1/cyclin D1 and Distance metastasis vs. Primary tissue by geographic area

Figure S22. Forest plot graphically representing the association between *CCND1*/cyclin D1 alterations and tisular expression (distance metastasis vs. primary tissue) in Cutaneous Melanomas by geographic area.

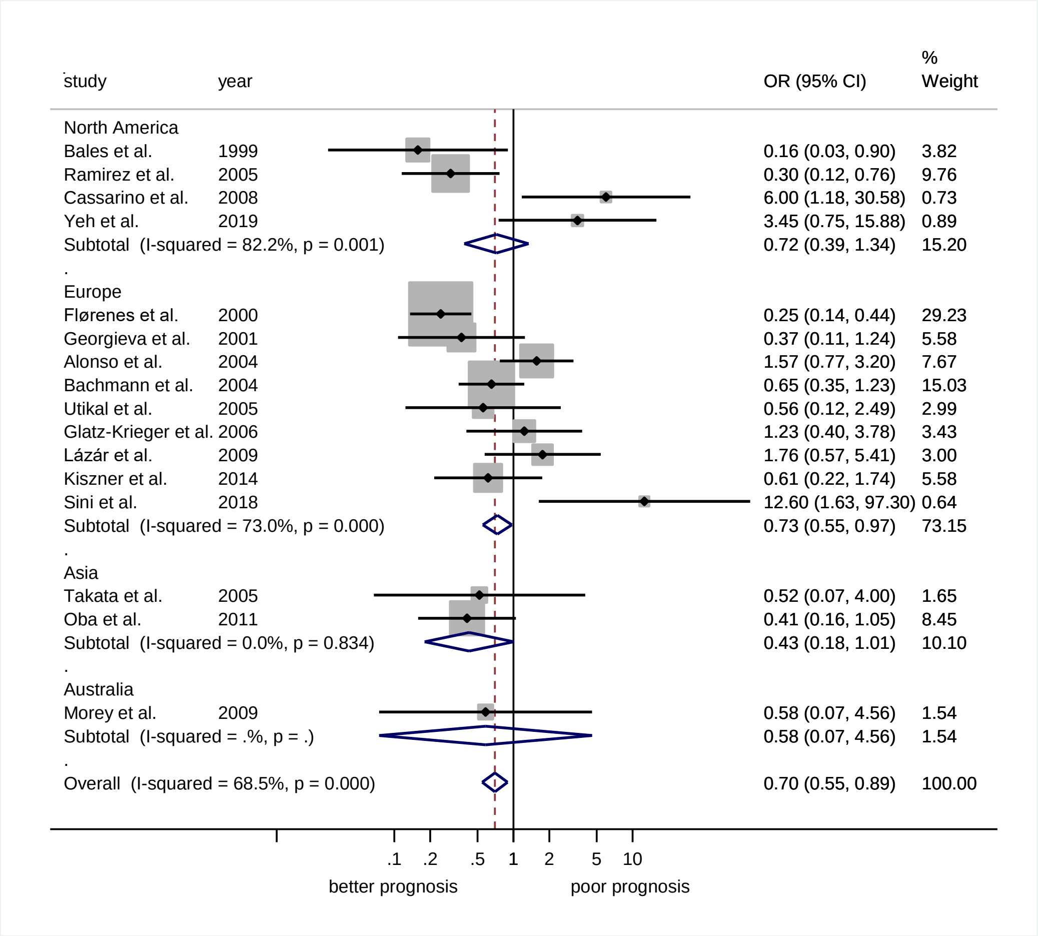

OR, odds ratio; CI, confidence intervals. Fixed-effect model (Mantel-Haenszel method) pooling odds ratios.

15.3 CCND1/cyclin D1 and Distance metastasis vs. Primary tissue by immunohistochemical pattern

Figure S23. Forest plot graphically representing the association between *CCND1*/cyclin D1 alterations and tisular expression (distance metastasis vs. primary tissue) in Cutaneous Melanomas by geographic area.

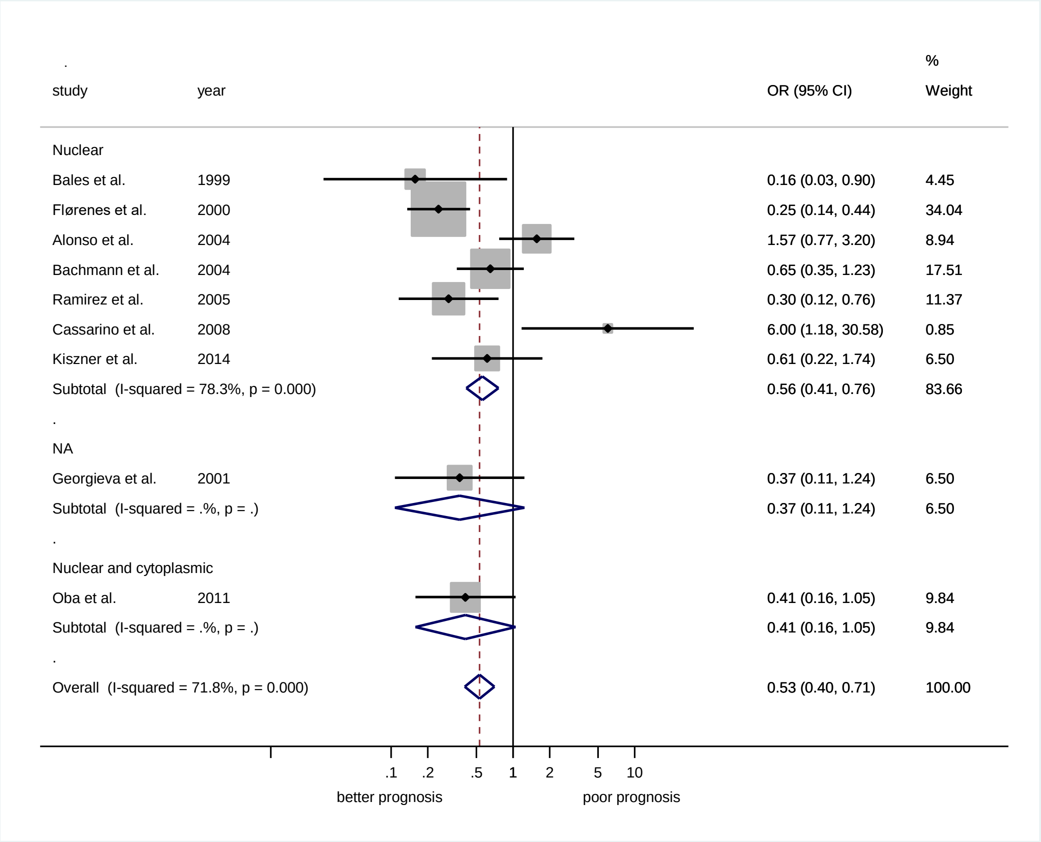

OR, odds ratio; CI, confidence intervals. Fixed-effect model (Mantel-Haenszel method) pooling odds ratios.

16. Meta-analysis on the frequency of CCND1/cyclin D1 alterations in uveal Melanoma

Figure S24. Forest plot graphically representing the frequency of *CCND1*/cyclin D1 alterations in Uveal Melanomas.

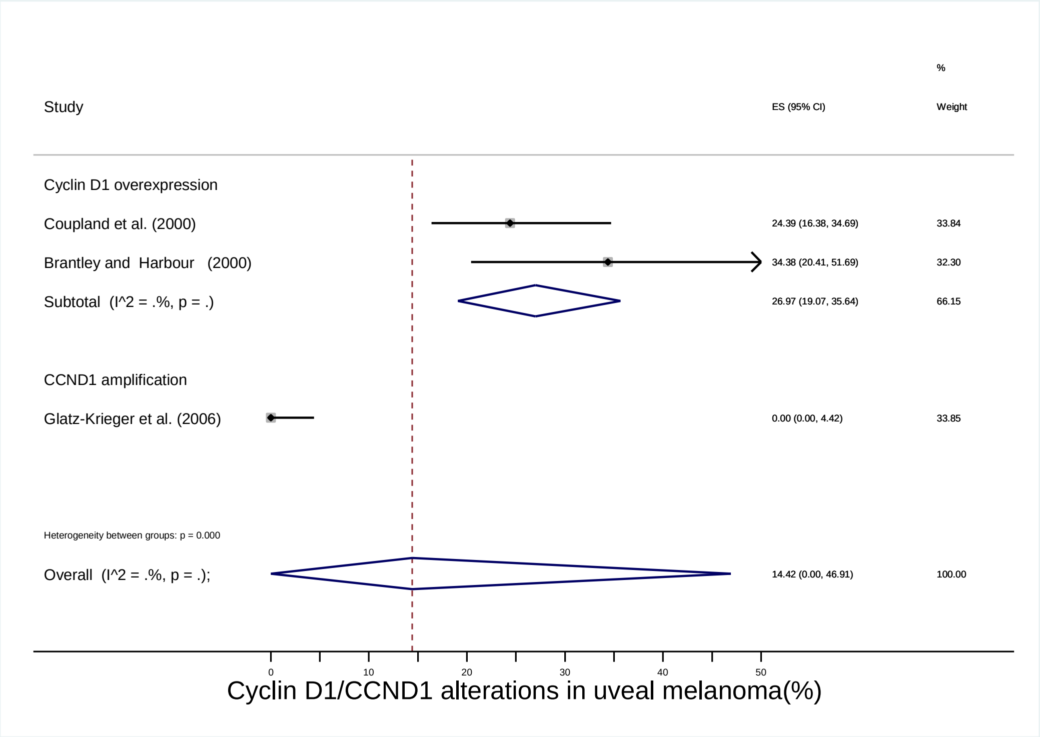

ES, estimation; CI, confidence intervals. Random-effects model (based on the DerSimonian and Laird method) using pooled proportions.

17. Meta-analysis on the association between CCND1/cyclin D1 alterations and Largest Basal Dimension in Uveal Melanoma

Figure S25. Forest plot graphically representing the association between *CCND1*/cyclin D1 alterations and Largest Basal Dimension in Uveal Melanomas.

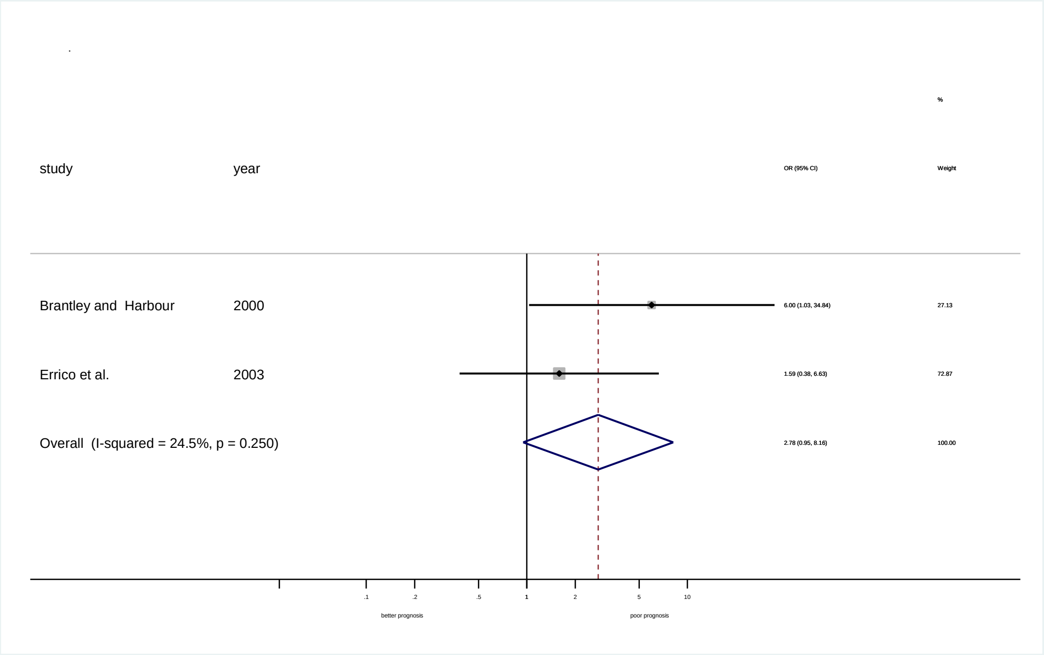

OR, odds ratio; CI, confidence intervals. Fixed-effect model (Mantel-Haenszel method) pooling odds ratios.

18. Meta-analysis on the association between CCND1/cyclin D1 alterations and Pathology in Uveal Melanoma

Figure S26. Forest plot graphically representing the association between *CCND1*/cyclin D1 alterations and pathology (epithelioid vs. spindle/mixed cell shape) in Uveal Melanomas.

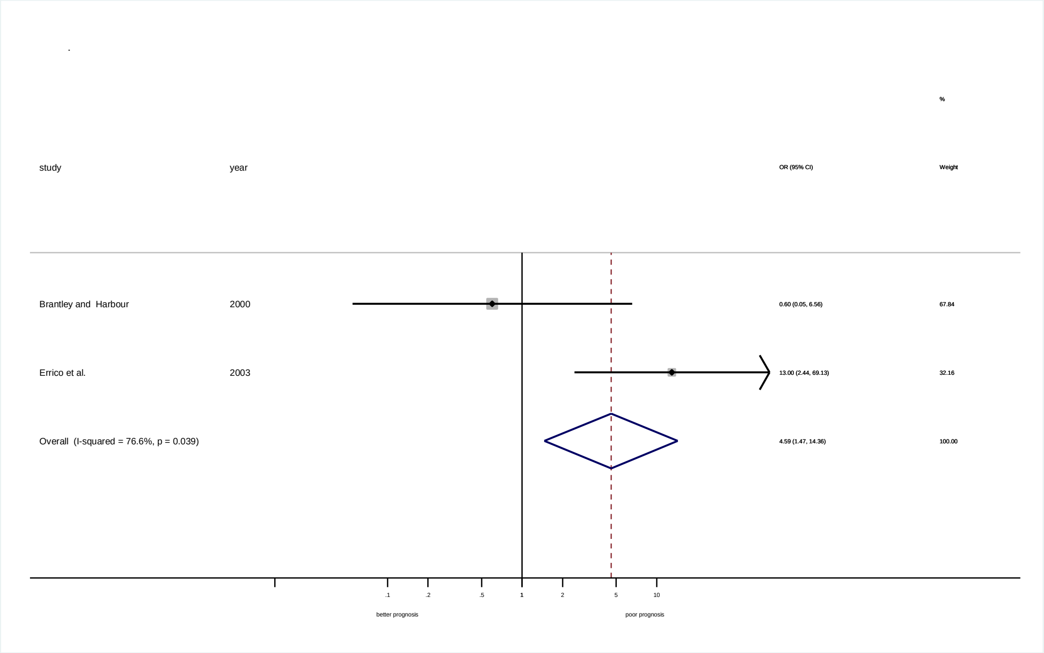

OR, odds ratio; CI, confidence intervals. Fixed-effect model (Mantel-Haenszel method) pooling odds ratios.

19. Meta-analysis on the frequency of CCND1/cyclin D1 alterations in Mucosal Melanoma

19.1 CCND1/cyclin D1 in Mucosal Melanomas by alteration

Figure S27. Forest plot graphically representing the frequency of *CCND1*/cyclin D1 alterations in Mucosal Melanomas.

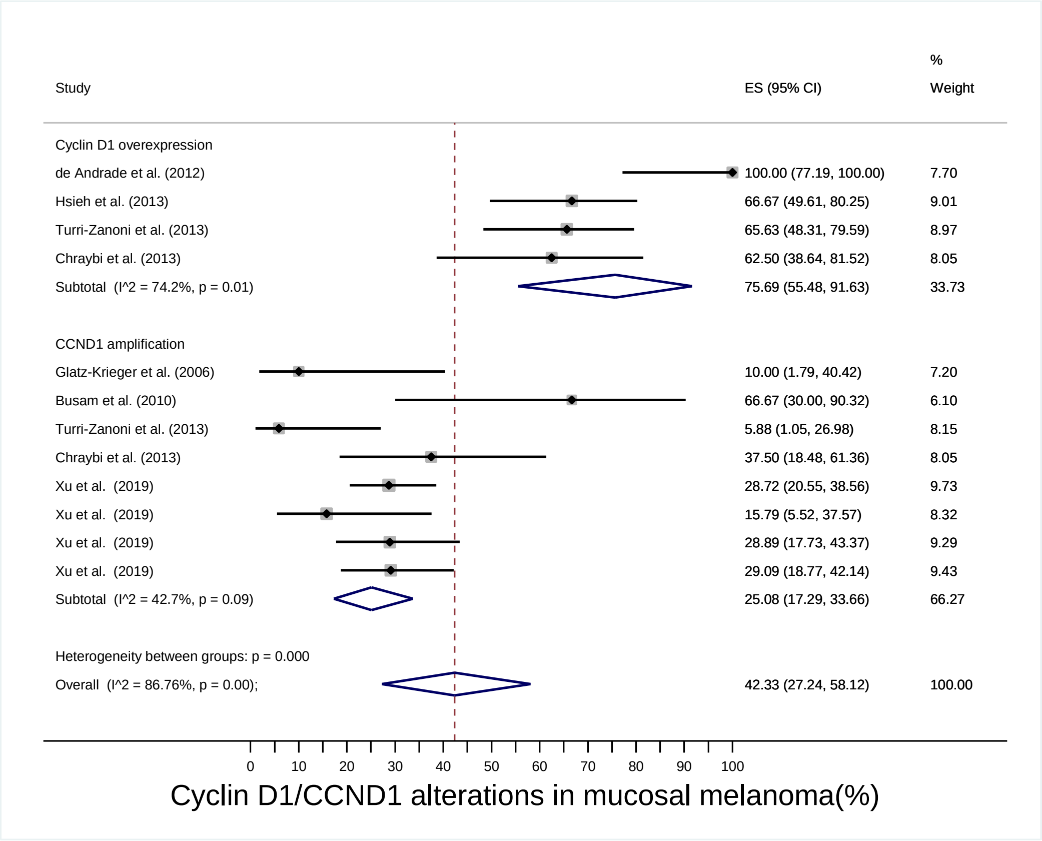

ES, estimation; CI, confidence intervals. Random-effects model (based on the DerSimonian and Laird method) using pooled proportions.

19.2 CCND1/cyclin D1 in Mucosal Melanomas by anatomical site

Figure S28. Forest plot graphically representing the frequency of *CCND1*/cyclin D1 alterations in Mucosal Melanomas by anatomical site.

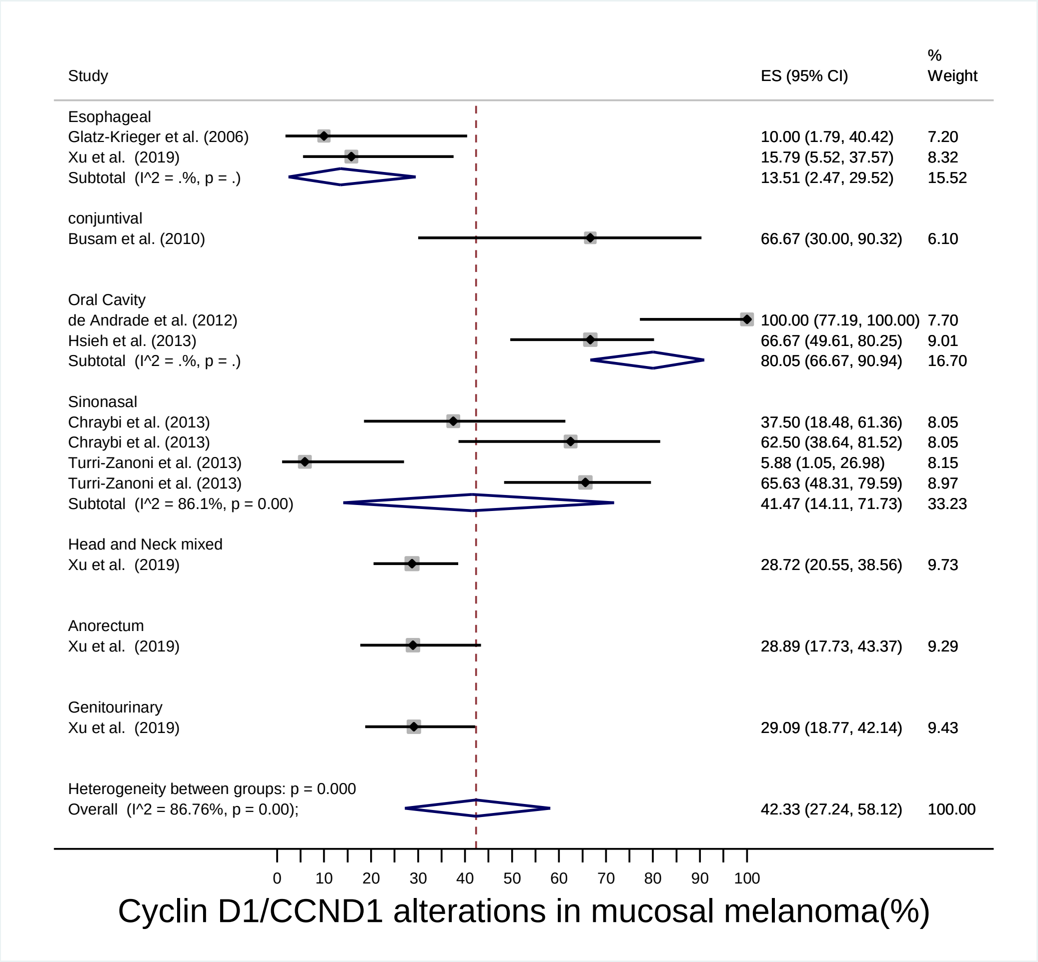

ES, estimation; CI, confidence intervals. Random-effects model (based on the DerSimonian and Laird method) using pooled proportions.

20. Meta-analysis on the association between CCND1/cyclin D1 alterations and Recurrence in Mucosal Melanoma

Figure S29. Forest plot graphically representing the association between *CCND1*/cyclin D1 alterations and Recurrence in Mucosal Melanomas.

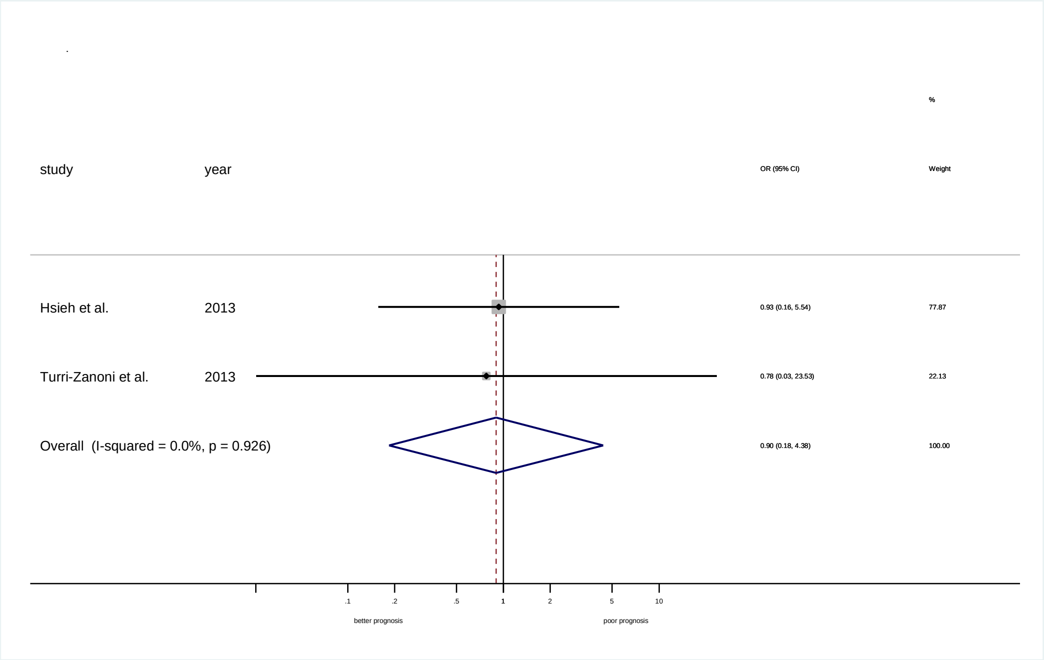

OR, odds ratio; CI, confidence intervals. Fixed-effect model (Mantel-Haenszel method) pooling odds ratios.

21. Meta-analysis on the association between CCND1/cyclin D1 alterations and Thickness in Mucosal Melanoma

Figure S30. Forest plot graphically representing the association between *CCND1*/cyclin D1 alterations and Thickness in Mucosal Melanomas.

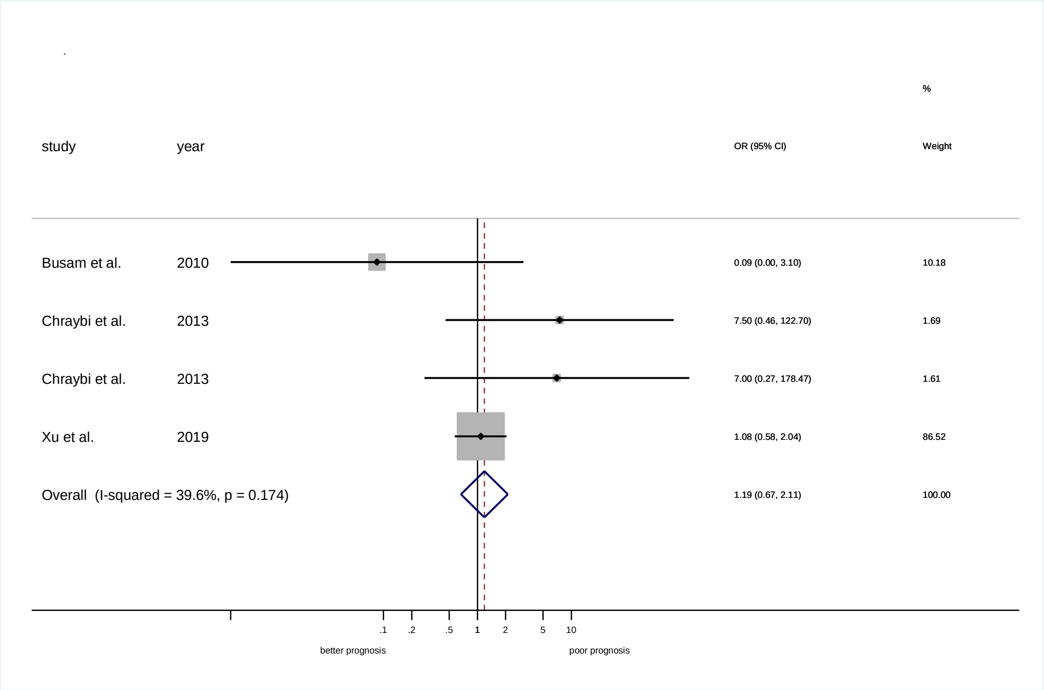

OR, odds ratio; CI, confidence intervals. Fixed-effect model (Mantel-Haenszel method) pooling odds ratios.

22. Meta-analysis on the association between CCND1/cyclin D1 alterations and M status in Mucosal Melanoma

Figure S31. Forest plot graphically representing the association between *CCND1*/cyclin D1 alterations and M status in Mucosal Melanomas.

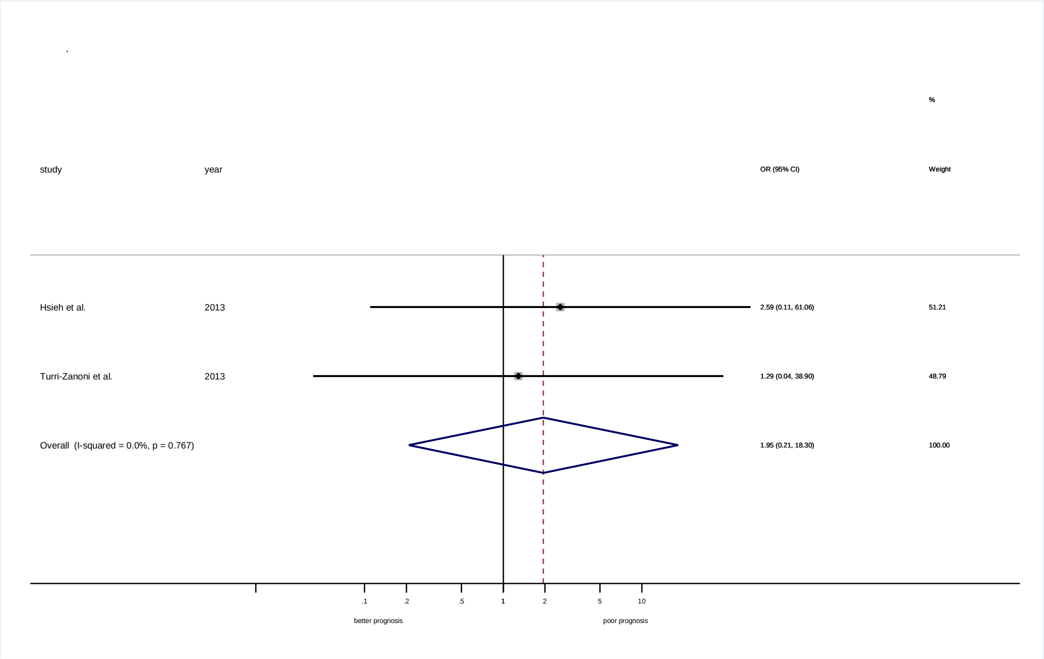

OR, odds ratio; CI, confidence intervals. Fixed-effect model (Mantel-Haenszel method) pooling odds ratios.

23. Meta-analysis on the association between CCND1/cyclin D1 alterations and necrosis in Mucosal Melanoma

Figure S32. Forest plot graphically representing the association between *CCND1*/cyclin D1 alterations and Necrosis in Mucosal Melanomas.

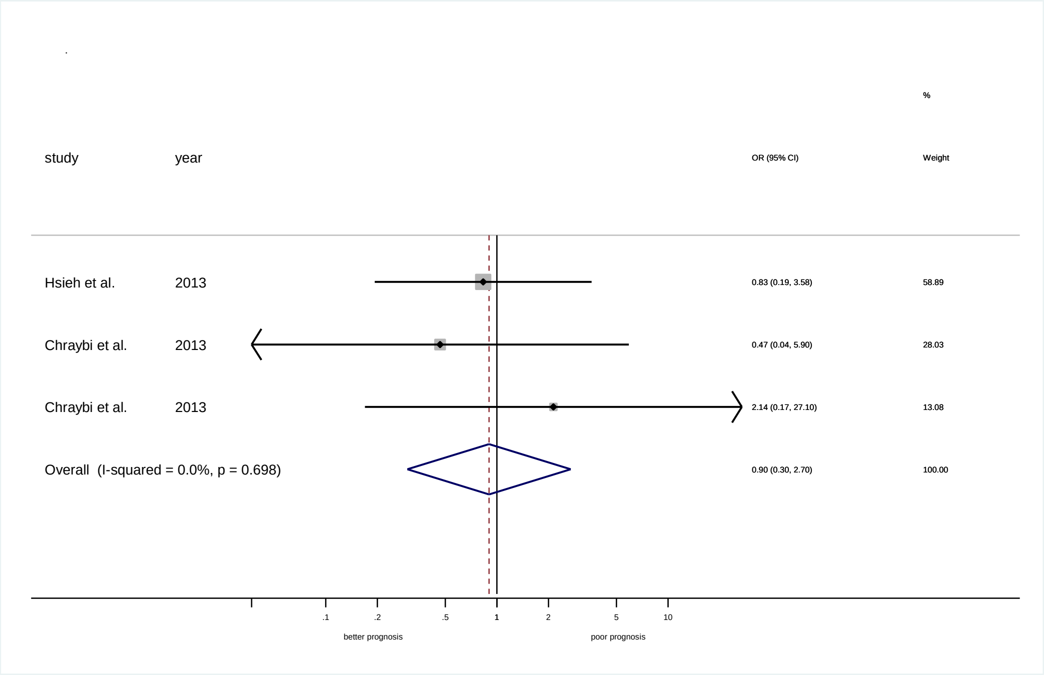

OR, odds ratio; CI, confidence intervals. Fixed-effect model (Mantel-Haenszel method) pooling odds ratios.

24. Analysis of small-study effects

24.1 CCND1/cyclin D1 alterations and Overall Survival in Cutaneous Melanomas

Figure 33. A funnel plot of estimated logHR against its standard error, graphically representing the analysis of small-study effects on Overall Survival in Cutaneous Melanomas.

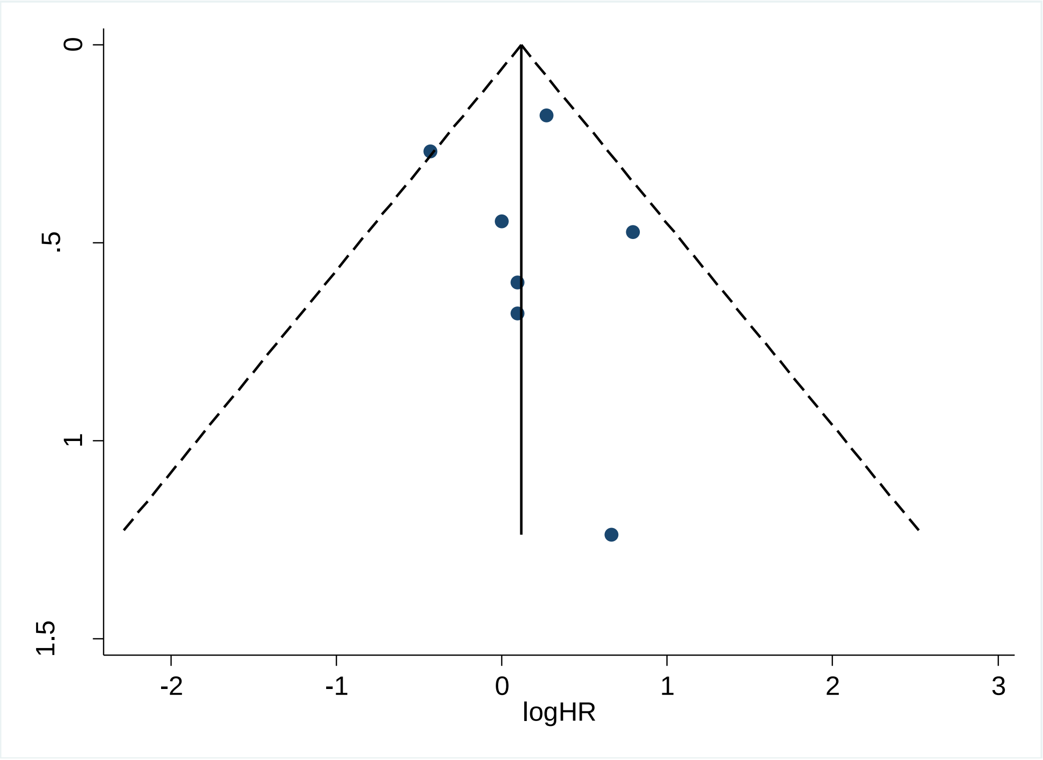

SE, standard error; HR, hazard ratio. The black vertical line corresponds to the pooled estimated prevalence. The two diagonal intermittent lines represent the pseudo-95% confidence interval. The circles represent the published studies.

24.2 CCND1/cyclin D1 alterations and Distance Metastasis vs. Primary tissue in Cutaneous Melanomas

Figure 34. A funnel plot of estimated logOR against its standard error, graphically representing the analysis of small-study effects on Distance Metastasis vs. Primary tissue in Cutaneous Melanomas.

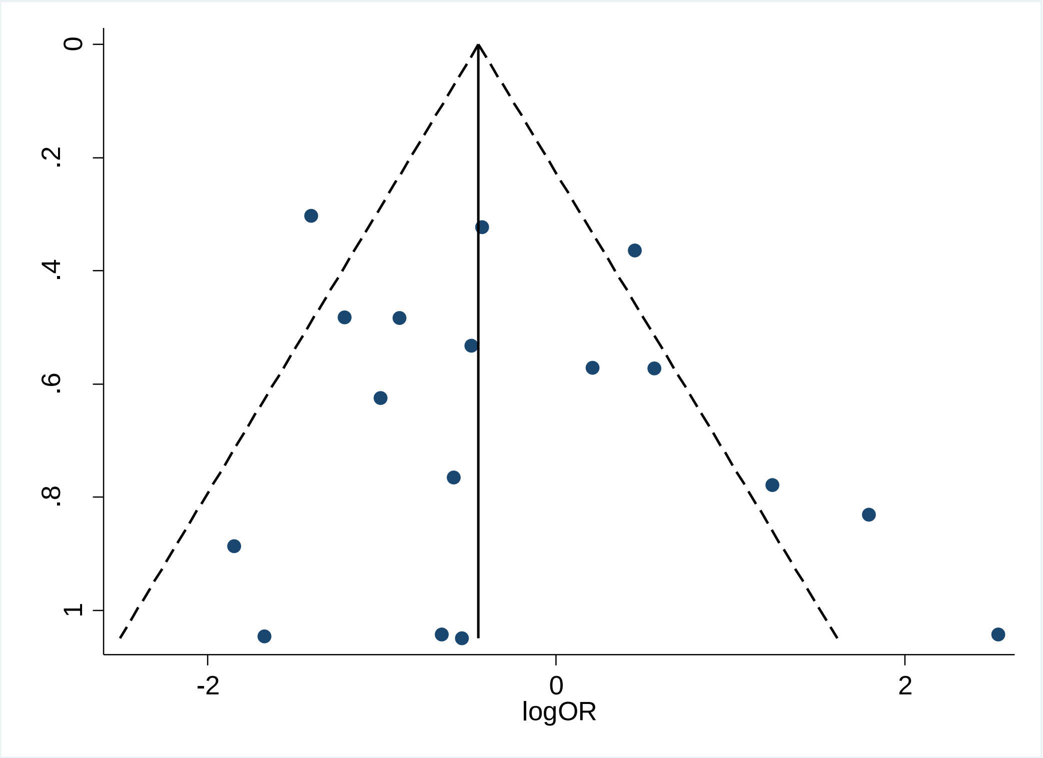

SE, standard error; OR, odds ratio. The black vertical line corresponds to the pooled estimated prevalence. The two diagonal intermittent lines represent the pseudo-95% confidence interval. The circles represent the published studies.

24.3 CCND1/cyclin D1 alterations and Type of Cutaneous Melanomas

Figure 35. A funnel plot of estimated logOR against its standard error, graphically representing the analysis of small-study effects on Type of Cutaneous Melanomas.

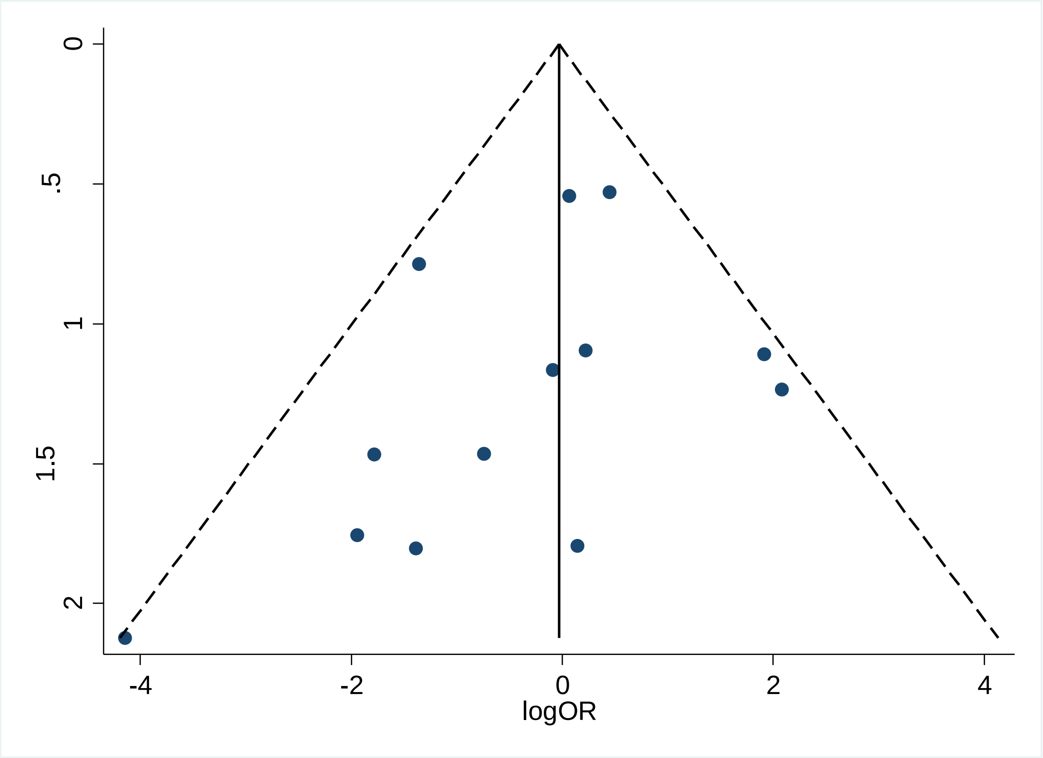

SE, standard error; OR, odds ratio. The black vertical line corresponds to the pooled estimated prevalence. The two diagonal intermittent lines represent the pseudo-95% confidence interval. The circles represent the published studies.

24.4 CCND1/cyclin D1 alterations and Ulceration in Cutaneous Melanomas

Figure 36. A funnel plot of estimated logOR against its standard error, graphically representing the analysis of small-study effects on Ulceration in Cutaneous Melanomas.

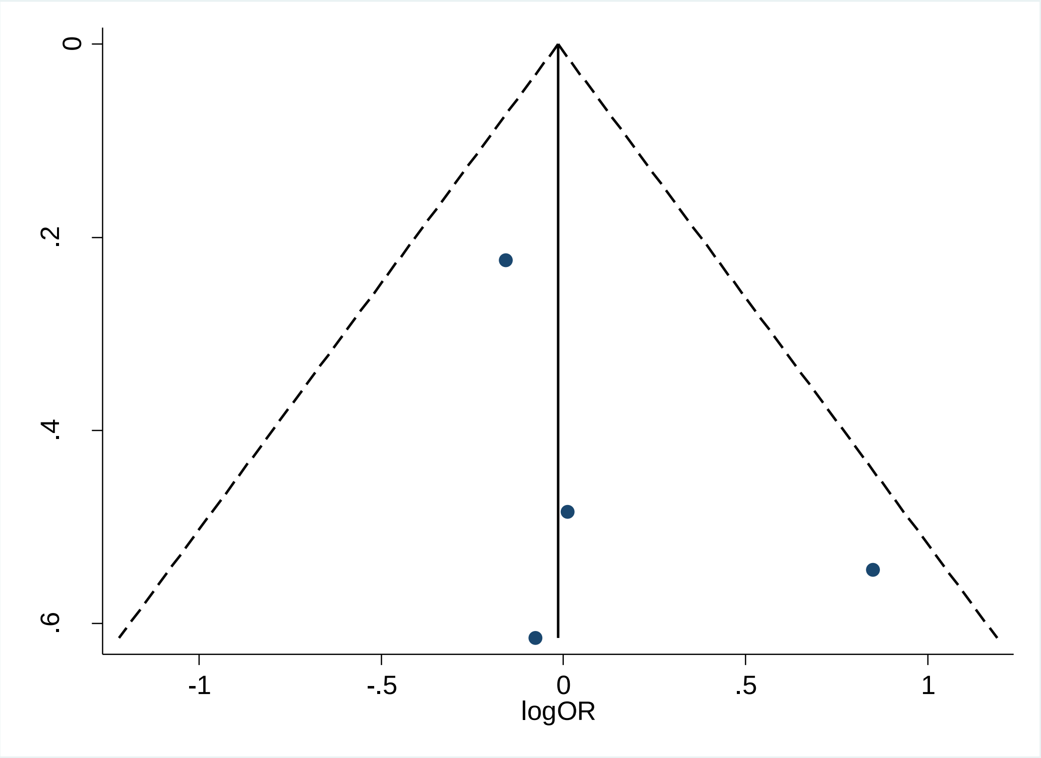

SE, standard error; OR, odds ratio. The black vertical line corresponds to the pooled estimated prevalence. The two diagonal intermittent lines represent the pseudo-95% confidence interval. The circles represent the published studies.

24.5 CCND1/cyclin D1 alterations and Clark levels in Cutaneous Melanomas

Figure 37. A funnel plot of estimated logOR against its standard error, graphically representing the analysis of small-study effects on Ulceration in Cutaneous Melanomas.

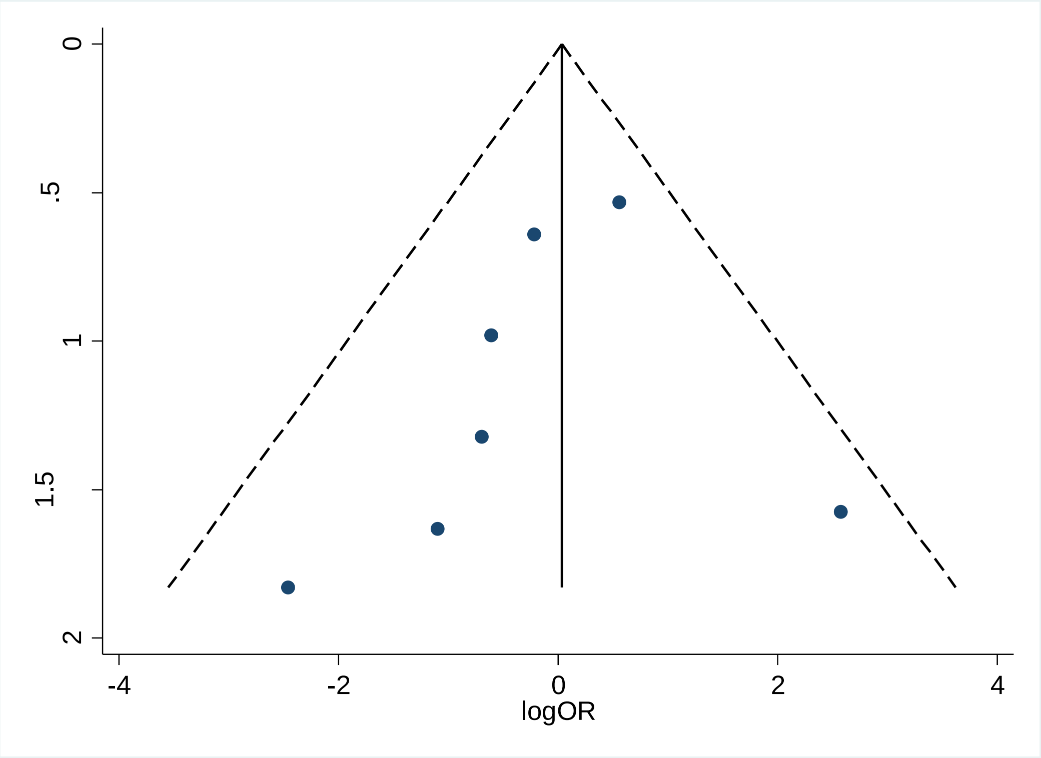

SE, standard error; OR, odds ratio. The black vertical line corresponds to the pooled estimated prevalence. The two diagonal intermittent lines represent the pseudo-95% confidence interval. The circles represent the published studies.

25 Sensitivity analysis (leave-one-out method).

25.1 CCND1/cyclin D1 and overall survival in cutaneous melanoma

Table S3. Sensitivity analysis of the studies pooled in the meta-analysis on the association between *CCND1*/cyclin D1 alterations and overall survival in cutaneous melanoma

Study omitted	Estimate	[95% Conf. Interval]	
Kong et al. (2017)	.99132621	0.65994644	1.4891021
Young et al. (2014)	1.1003208	0.77865422	1.5548697
Jurmeister et al. (2019)	1.0640217	0.81877315	1.38273
Flørenes et al. (2000)	1.1319236	0.77194953	1.6597601
Alonso et al. (2004)	1.1149724	0.77112538	1.6121417
Bachmann et al. (2004)	1.3211365	0.99138665	1.7605659
Oba et al. (2011)	1.114519	0.77431935	1.6041865
**Combined**	1.1109668	0.81321977	1.5177289

Sensitivity analysis ("leave-one-out" method) of the meta-analysis results, sequentially omitting one study at a time.

25.2 CCND1/cyclin D1 and Breslow Thickness in cutaneous melanoma

Table S4. Sensitivity analysis of the studies pooled in the meta-analysis on the association between *CCND1*/cyclin D1 alterations and Breslow Thickness in cutaneous melanoma

**Study omitted**	**Estimate**	**[95% Conf.Interval]**	
Bales et al. (1999)	1.5356706	1.0744609	2.1948535
Georgieva et al. (2001)	1.4116368	.97337258	2.047231
Curtin et al. (2005)	1.5370564	1.0684719	2.2111413
Utikal et al. (2005)	1.5671294	1.0913483	2.2503307
Glatz-Krieger et al. (2006)	1.5022691	1.0398775	2.1702678
Stefanaki et al. (2007)	1.6471213	1.1468467	2.3656244
Gerami et al. (2009)	1.5582688	1.0900351	2.2276361
Lázár et al. (2009)	1.5873477	1.0884719	2.3148718
Nai et al. (2011)	1.5453309	1.0764588	2.2184291
Oba et al. (2011)	1.4687469	1.0015131	2.1539581
Diaz et al. (2014)	1.5062068	1.0479358	2.1648831
Kiszner et al. (2014)	1.4033892	.96577382	2.0392985
Young et al. (2014)	1.6556984	1.1294576	2.4271274
Romano et al. (2016)	1.5179224	1.0621439	2.1692808
Donigan et al. (2017)	1.5668489	1.0944691	2.2431107
Su et al. (2017)	1.6822562	1.1588465	2.4420714
**Combined**	1.5416614	1.0810305	2.1985689

Sensitivity analysis ("leave-one-out" method) of the meta-analysis results, sequentially omitting one study at a time.

25.3 CCND1/cyclin D1 and Ulceration in cutaneous melanoma

Table S5. Sensitivity analysis of the studies pooled in the meta-analysis on the association between *CCND1*/cyclin D1 alterations and Ulceration in cutaneous melanoma

**Study omitted**	**Estimate**	**[95% Conf. Interval]**	
Lázár et al. (2009)	.88384908	.60531014	1.2905604
Young et al. (2014)	1.0819124	.60832918	1.9241794
Kong et al. (2017)	1.3015442	.70610696	2.3990946
Su et al. (2017)	1.0804726	.63683039	1.8331741
**Combined**	.98528418	.68962828	1.407693

Sensitivity analysis ("leave-one-out" method) of the meta-analysis results, sequentially omitting one study at a time.

25.4 CCND1/cyclin D1 and Clark in cutaneous melanoma

Table S6. Sensitivity analysis of the studies pooled in the meta-analysis on the association between *CCND1*/cyclin D1 alterations and Clark in cutaneous melanoma

**Study omitted**	**Estimate**	**[95% Conf. Interval]**	
Utikal et al. (2005)	.90166622	.46596026	1.7447882
Stefanaki et al. (2007)	1.1636142	.60903102	2.2232003
Morey et al. (2009)	1.1555958	.61029744	2.1881161
Lázár et al. (2009)	.84210449	.38295254	1.8517699
Diaz et al. (2014)	1.1920958	.61622804	2.3061144
Romano et al. (2016)	1.2369797	.64777076	2.3621302
Su et al. (2017)	1.222731	.59346306	2.519232
**Combined**	1.1031679	.59078521	2.0599353

Sensitivity analysis (“leave-one-out” method) of the meta-analysis results, sequentially omitting one study at a time.

25.5 CCND1/cyclin D1 and Type of cutaneous melanoma

Table S7. Sensitivity analysis of the studies pooled in the meta-analysis on the association between *CCND1*/cyclin D1 alterations and Type of cutaneous melanoma.

**Study omitted**	**Estimate**	**[95% Conf. Interval]**	
Bales et al. (1999)	.85516542	.52791876	1.3852658
Flørenes et al. (2000)	.78720784	.46082872	1.344743
Sauter et al. (2002)	.92139494	.5631001	1.5076692
Sauter et al. (2002)	1.007732	.60234076	1.6859623
Rosenwald et al. (2003)	.92139488	.56562394	1.5009416
Utikal et al. (2005)	.73446327	.44496459	1.2123128
Curtin et al. (2005)	.84937412	.52333879	1.3785266
Stefanaki et al. (2007)	.81941396	.50139642	1.3391384
Morey et al. (2009)	.83158827	.5131101	1.3477401
Lázár et al. (2009)	.70764017	.40940812	1.2231184
Nai et al. (2011)	.83316571	.51112133	1.3581221
Romano et al. (2016)	.87471694	.5388661	1.4198885
Haugh et al. (2018)	.76160532	.46451521	1.2487054
**Combined**	.83667837	.51879367	1.3493432

Sensitivity analysis ("leave-one-out" method) of the meta-analysis results, sequentially omitting one study at a time.

25.6 CCND1/cyclin D1 and Distance metastasis vs primary tissue in cutaneous melanoma

Table S8. Sensitivity analysis of the studies pooled in the meta-analysis on the association between *CCND1*/cyclin D1 alterations and Distance metastasis vs primary in cutaneous melanoma

**Study omitted**	**Estimate**	**[95% Conf. Interval]**	
Bales et al. (1999)	.71988779	.562841	.92075461
Flørenes et al. (2000)	.88554162	.67446262	1.1626797
Georgieva et al. (2001)	.71808535	.56011772	.92060399
Alonso et al. (2004)	.62613755	.48282185	.81199354
Bachmann et al. (2004)	.70646119	.54292858	.91925055
Utikal et al. (2005)	.70281672	.54929817	.89924079
Ramirez et al. (2005)	.74185169	.57648784	.95464963
Takata et al. (2005)	.70144165	.54906416	.89610732
Glatz-Krieger et al. (2006)	.67948002	.52983105	.87139672
Cassarino et al. (2008)	.65940285	.51483655	.84456342
Morey et al. (2009)	.70021999	.54812616	.89451671
Lázár et al. (2009)	.66556609	.51835096	.85459137
Oba et al. (2011)	.72528493	.56384766	.93294376
Kiszner et al. (2014)	.70341218	.5478285	.90318185
Sini et al. (2018)	.62192225	.48341301	.80011761
Yeh et al. (2019)	.67377269	.52672511	.86187202
**Combined**	.69841473	.54765845	.89067034

Sensitivity analysis (“leave-one-out” method) of the meta-analysis results, sequentially omitting one study at a time.

26. List of included studies in this systematic review and meta-analysis.

Alonso SR, Ortiz P, Pollán M, Pérez-Gómez B, Sánchez L, Acuña MJ, Pajares R, Martínez-Tello FJ, Hortelano CM, Piris MA, Rodríguez-Peralto JL. 2004. Progression in cutaneous malignant melanoma is associated with distinct expression profiles: a tissue microarray-based study. Am J Pathol [Internet]. 164(1):193–203. http://www.ncbi.nlm.nih.gov/pubmed/14695333

de Andrade BAB, León JE, Carlos R, Delgado-Azañero W, Mosqueda-Taylor A, de Almeida OP. 2012. Immunohistochemical expression of p16, p21, p27 and cyclin D1 in oral nevi and melanoma. Head Neck Pathol [Internet]. 6(3):297–304. http://www.ncbi.nlm.nih.gov/pubmed/22311377

Bachmann IM, Straume O, Akslen LA. 2004. Altered expression of cell cycle regulators Cyclin D1, p14, p16, CDK4 and Rb in nodular melanomas. Int J Oncol. 25(6):1559–1565.

Bales E, Dietrich C, Bandyopadhyay D, Schwahn D, Xu W, Didenko V, Leiss P, Conrad N, Pereira-Smith O, Orengo I, Medrano E. 1999. High levels of expression of p27KIP1 and cyclin E in invasive primary malignant melanomas. J Invest Dermatol. 113(6):1039–46.

Brantley MJ, Harbour J. 2000. Deregulation of the Rb and p53 pathways in uveal melanoma. Am J Pathol. 157(6):1795–801.

Busam KJ, Fang Y, Jhanwar SC, Pulitzer MP, Marr B, Abramson DH. 2010. Distinction of conjunctival melanocytic nevi from melanomas by fluorescence in situ hybridization. J Cutan Pathol. 37(2):196–203.

Cassarino DS, Cabral ES, Kartha R V., Swetter SM. 2008. Primary dermal melanoma: Distinct immunohistochemical findings and clinical outcome compared with nodular and metastatic melanoma. Arch Dermatol. 144(1):49–56.

Chraybi M, Abd Alsamad I, Copie-Bergman C, Baia M, André J, Dumaz N, Ortonne N. 2013. Oncogene abnormalities in a series of primary melanomas of the sinonasal tract: NRAS mutations and cyclin D1 amplification are more frequent than KIT or BRAF mutations. Hum Pathol [Internet]. 44(9):1902–11. http://www.ncbi.nlm.nih.gov/pubmed/23664541

Coupland SE, Anastassiou G, Stang A, Schilling H, Anagnostopoulos I, Bornfeld N, Stein H. 2000. The prognostic value of cyclin D1, p53, and MDM2 protein expression in uveal melanoma. J Pathol [Internet]. 191(2):120–126. http://doi.wiley.com/10.1002/%28SICI%291096-9896%28200006%29191%3A2%3C120%3A%3AAID-PATH591%3E3.0.CO%3B2-P

Curtin JA, Fridlyand J, Kageshita T, Patel HN, Busam KJ, Kutzner H, Cho K-H, Aiba S, Bröcker E-B, LeBoit PE, et al. 2005. Distinct Sets of Genetic Alterations in Melanoma. N Engl J Med [Internet]. 353(20):2135–2147. http://www.nejm.org/doi/abs/10.1056/NEJMoa050092

Diaz A, Puig-Butillé JA, Valera A, Muñoz C, Costa D, Garcia-Herrera A, Carrera C, Sole F, Malvehy J, Puig S, Alos L. 2014. TERT and AURKA gene copy

number gains enhance the detection of acral lentiginous melanomas by fluorescence in situ hybridization. J Mol Diagnostics. 16(2):198–206.

Donigan JM, De Luca J, Lum C. 2017. Cyclin D1 and p16 Expression in Blue Nevi and Malignant Melanoma. Appl Immunohistochem Mol Morphol AIMM [Internet]. 25(2):91–94. http://www.ncbi.nlm.nih.gov/pubmed/26766120

E.R. S, U.-C. Y, A. VS, W. Z, S. L, D.S. T, G. P, M. N, D. P, M. H, B.C. B. 2002. Cyclin D1 is a candidate oncogene in cutaneous melanoma. Cancer Res [Internet]. 62(11):3200–3206. http://www.embase.com/search/results?subaction=viewrecord&from=export&id=L34602414

Errico ME, Staibano S, Tranfa F, Bonavolonta G, Lo Muzio L, Somma P, Lucariello A, Mansueto G, D'Aponte A, Ferrara G, De Rosa G. 2003. Expression of cyclin-D1 in uveal malignant melanoma. Anticancer Res. 23(3 B):2701–2706.

Florenes VA, Faye RS, Maelandsmo GM, Nesland JM, Holm R. 2000. Levels of cyclin d1 and d3 in malignant melanoma: Deregulated cyclin D3 expression is associated with poor clinical outcome in superficial melanoma. Clin Cancer Res [Internet]. 6(9):3614–3620. http://www.embase.com/search/results?subaction=viewrecord&from=export&id=L30694945

Georgieva J, Sinha P, Schadendorf D. 2001. Expression of cyclins and cyclin dependent kinases in human benign and malignant melanocytic lesions. J Clin Pathol [Internet]. 54(3):229–35. http://www.ncbi.nlm.nih.gov/pubmed/11253137

Gerami P, Wass A, Mafee M, Fang Y, Pulitzer MP, Busam KJ. 2009. Fluorescence in situ hybridization for distinguishing nevoid melanomas from mitotically active nevi. Am J Surg Pathol. 33(12):1783–1788.

Glatz-Krieger K, Pache M, Tapia C, Fuchs A, Savic S, Glatz D, Mihatsch M, Meyer P. 2006. Anatomic site-specific patterns of gene copy number gains in skin, mucosal, and uveal melanomas detected by fluorescence in situ hybridization. Virchows Arch. 449(3):328–333.

Haugh AM, Zhang B, Quan VL, Garfield EM, Bubley JA, Kudalkar E, Verzi AE, Walton K, VandenBoom T, Merkel EA, et al. 2018. Distinct Patterns of Acral Melanoma Based on Site and Relative Sun Exposure. J Invest Dermatol. 138(2):384–393.

Hsieh R, Nico MMS, Coutinho-Camillo CM, Buim ME, Sangueza M, Lourenço S V. 2013. The CDKN2A and MAP kinase pathways: Molecular roads to primary oral mucosal melanoma. Am J Dermatopathol. 35(2):167–175.

J.A. R, J. G, M.S. R, L.K. D. 2005. Cyclin D1 expression in melanocytic lesions of the skin. Ann Diagn Pathol [Internet]. 9(4):185–188. http://www.embase.com/search/results?subaction=viewrecord&from=export&id=L41111827%0Ahttp://dx.doi.org/10.1016/j.anndiagpath.2005.04.018

Jurmeister P, Bockmayr M, Treese C, Stein U, Lenze D, Jöhrens K, Friedling F, Dietel M, Klauschen F, Marsch W, et al. 2019. Immunohistochemical analysis of

Bcl-2, nuclear S100A4, MITF and Ki67 for risk stratification of early-stage melanoma – A combined IHC score for melanoma risk stratification. JDDG - J Ger Soc Dermatology. 17(8):800–808.

Kiszner G, Wichmann B, Nemeth IB, Varga E, Meggyeshazi N, Teleki I, Balla P, Maros ME, Penksza K, Krenacs T. 2014. Cell cycle analysis can differentiate thin melanomas from dysplastic nevi and reveals accelerated replication in thick melanomas. Virchows Arch [Internet]. 464(5):603–12. http://www.ncbi.nlm.nih.gov/pubmed/24682564

Kong Y, Sheng X, Wu X, Yan J, Ma M, Yu J, Si L, Chi Z, Cui C, Dai J, et al. 2017. Frequent genetic aberrations in the CDK4 pathway in acral melanoma indicate the potential for CDK4/6 inhibitors in targeted therapy. Clin Cancer Res. 23(22):6946–6957.

Lázár V, Ecsedi S, Szöllosi AG, Tóth R, Vízkeleti L, Rákosy Z, Bégány A, Adány R, Balázs M. 2009. Characterization of candidate gene copy number alterations in the 11q13 region along with BRAF and NRAS mutations in human melanoma. Mod Pathol [Internet]. 22(10):1367–78. http://www.ncbi.nlm.nih.gov/pubmed/19633643

Morey AL, Murali R, McCarthy SW, Mann GJ, Scolyer RA. 2009. Diagnosis of cutaneous melanocytic tumours by four-colour fluorescence in situ hybridisation. Pathology. 41(4):383–387.

Nai G, Marques M. 2011. Role of ROC1 protein in the control of cyclin D1 protein expression in skin melanomas. Pathol Res Pract. 207(3):174–181.

Nathanson KL, Martin A-M, Wubbenhorst B, Greshock J, Letrero R, D'Andrea K, O'Day S, Infante JR, Falchook GS, Arkenau H-T, et al. 2013. Tumor Genetic Analyses of Patients with Metastatic Melanoma Treated with the BRAF Inhibitor Dabrafenib (GSK2118436). Clin Cancer Res [Internet]. 19(17):4868–4878. http://clincancerres.aacrjournals.org/cgi/doi/10.1158/1078-0432.CCR-13-0827

Oba J, Nakahara T, Abe T, Hagihara A, Moroi Y, Furue M. 2011. Expression of c-Kit, p-ERK and cyclin D1 in malignant melanoma: An immunohistochemical study and analysis of prognostic value. J Dermatol Sci [Internet]. 62(2):116–123. https://linkinghub.elsevier.com/retrieve/pii/S092318111100079X

Requena C, Rubio L, Traves V, Sanmartín O, Nagore E, Llombart B, Serra C, Fernández-Serra A, Botella R, Guillén C. 2012. Fluorescence in situ hybridization for the differential diagnosis between Spitz naevus and spitzoid melanoma. Histopathology. 61(5):899–909.

Romano RC, Shon W, Sukov WR. 2016. Malignant Melanoma of the Nail Apparatus: A Fluorescence in Situ Hybridization Analysis of 7 Cases. Int J Surg Pathol. 24(6):512–518.

Rosenwald IB, Wang S, Savas L, Woda B, Pullman J. 2003. Expression of translation initiation factor eIF-2? is increased in benign and malignant melanocytic and colonic epithelial neoplasms. Cancer [Internet]. 98(5):1080–1088. http://doi.wiley.com/10.1002/cncr.11619

Sini MC, Doneddu V, Paliogiannis P, Casula M, Colombino M, Manca A, Botti G, Ascierto PA, Lissia A, Cossu A, Palmieri G. 2018. Genetic alterations in main candidate genes during melanoma progression. Oncotarget. 9(9):8531–8541.

Stefanaki C, Stefanaki K, Antoniou C, Argyrakos T, Patereli A, Stratigos A, Katsambas A. 2007. Cell cycle and apoptosis regulators in Spitz nevi: Comparison with melanomas and common nevi. J Am Acad Dermatol. 56(5):815–824.

Su J, Yu W, Liu J, Zheng J, Huang S, Wang Y, Qi S, Ma X, Chen J, Zhang Y. 2017. Fluorescence in situ hybridisation as an ancillary tool in the diagnosis of acral melanoma: a review of 44 cases. Pathology. 49(7):740–749.

Takata M, Goto Y, Ichii N, Yamaura M, Murata H, Koga H, Fujimoto A, Saida T. 2005. Constitutive Activation of the Mitogen-Activated Protein Kinase Signaling Pathway in Acral Melanomas. J Invest Dermatol [Internet]. 125(2):318–322. http://linkinghub.elsevier.com/retrieve/pii/S0022202X15324064

Turri-Zanoni M, Medicina D, Lombardi D, Ungari M, Balzarini P, Rossini C, Pellegrini W, Battaglia P, Capella C, Castelnuovo P, et al. 2013. Sinonasal mucosal melanoma: Molecular profile and therapeutic implications from a series of 32 cases. Head Neck. 35(8):1066–1077.

Utikal J, Udart M, Leiter U, Peter RU, Krähn G. 2005. Additional Cyclin D(1) gene copies associated with chromosome 11 aberrations in cutaneous malignant melanoma. Int J Oncol [Internet]. 26(3):597–605. http://www.ncbi.nlm.nih.gov/pubmed/15703813

Xu L, Cheng Z, Cui C, Wu X, Yu H, Guo J, Kong Y. 2019. Frequent genetic aberrations in the cell cycle related genes in mucosal melanoma indicate the potential for targeted therapy. J Transl Med. 17(1).

Yeh I, Jorgenson E, Shen L, Xu M, North JP, Shain AH, Reuss D, Wu H, Robinson WA, Olshen A, et al. 2019. Targeted Genomic Profiling of Acral Melanoma. JNCI J Natl Cancer Inst [Internet]. 111(10):1068–1077. https://academic.oup.com/jnci/article/111/10/1068/5292473

Young RJ, Waldeck K, Martin C, Foo JH, Cameron DP, Kirby L, Do H, Mitchell C, Cullinane C, Liu W, et al. 2014. Loss of CDKN2A expression is a frequent event in primary invasive melanoma and correlates with sensitivity to the CDK4/6 inhibitor PD0332991 in melanoma cell lines. Pigment Cell Melanoma Res. 27(4):590–600.

27. Protocol

Review title.

Prognostic and clinicopathological significance of *CCND1*/cyclin D1 alterations in melanomas: a systematic review and meta-analysis protocol.

Anticipated or actual start date.

September, 2019

Anticipated completion date.

July, 2020

Stage of review at time of protocol submission (October, 2019)

Review stage	Started	Completed
Preliminary searches	Yes	Yes
Piloting of the study selection process	Yes	Yes
Formal screening of search results against eligibility criteria	Yes	No
Data extraction	No	No
Risk of bias (quality) assessment	No	No
Data analysis	No	No

Review team members and their organisational affiliations.

Lucía González-Ruiz - Dermatology Service, Ciudad Real General University Hospital, Ciudad Real, Spain.

Miguel Ángel González-Moles - School of Dentistry, University of Granada, Granada, Spain/Instituto de Investigación Biosanitaria ibs.GRANADA, Granada, Spain.

Isabel González-Ruiz - School of Dentistry, University of Granada, Granada, Spain.

Isabel Ruiz-Ávila - Pathology Service, San Cecilio Hospital Complex, Granada, Spain/Instituto de Investigación Biosanitaria ibs.GRANADA, Granada, Spain.

Pablo Ramos-García - School of Dentistry, University of Granada, Granada, Spain/Instituto de Investigación Biosanitaria ibs.GRANADA, Granada, Spain.

Funding sources/sponsors.

Research Group CTS-392 (Plan Andaluz de Investigación, Spain).

Review question.

What is the clinicopathological and prognostic significance of CCND1/cyclin D1 alterations in patients with melanomas?

Searches.

PubMed - ("cyclin d1"[MeSH Terms] OR ("cyclin"[All Fields] AND "d1"[All Fields]) OR "cyclin d1"[All Fields] OR "cyclind1"[All Fields] OR “ccnd1”[All Fields] OR “ccnd 1”[All Fields]) AND ("melanoma"[MeSH Terms] OR "melanoma"[All Fields])

Embase - ('cyclin d1'/exp OR 'cyclin d1' OR 'cyclind1' OR 'ccnd1' OR 'ccnd 1') AND ('melanoma'/exp OR 'melanoma')

Web of Science - TS=(cyclin d1 OR cyclind1 OR ccnd1 OR ccnd 1) AND TS=(melanoma)

Scopus - TITLE-ABS-KEY(("cyclin d1" OR "cyclind1" OR "ccnd1" OR "ccnd 1") AND ("melanoma"))

In adittion, a manual screening of the references lists of the included articles will also be performed to find relevant articles that might have been missed by the original search strategy.

Condition or domain being studied.

Malignant melanoma is the most aggressive form of skin cancer with unpredictable behavior. Although multiple approaches have been used in its treatment, the mortality of melanoma patients has barely improved in recent decades. Therefore, in addition to conventional clinicopathological prognostic parameters (e.g. Breslow thickness), the early prediction of the evolution of these patients through the use of molecular biomarkers could also be a promising tool in daily clinical practice.

Participants/population.

Patients diagnosed with melanoma.

Intervention(s), exposure(s).

We will evaluate studies in which *CCND1*/cyclin D1 alterations were analyzed in tumor tissue from patients diagnosed with melanoma.

Differences in the amplification levels of *CCND1* will be categorized as positive for the exposition group, and negative for the control group, based on the cut-off value chosen by the authors. Differences in the expression of cyclin D1 will be categorized as high expression/overexpression for the exposition group and low expression for the control group, based on the cut-off value chosen by authors.

Comparator(s)/control.

Control group will be represented by the group of patients with melanomas with negative *CCND1* amplification or low cyclin D1 expression.

Types of study to be included.

Criteria for eligibility of a study included in the qualitative and quantitative analysis will be:

- Original research articles published in English language.

- To assess CCND1/cyclin D1 alterations (gene amplification or protein expression) in biopsies from patients diagnosed with melanomas.

- To analyze the associations between CCND1/cyclin D1 alterations with survival endpoints (overall and/or disease-free survival) and/or clinicopathological features (type of melanoma, subtype, Breslow thickness, ulceration, N and M status, clinical stage, Clark level, growth phases, regression, satellitosis, mitotic index, anatomic location, sex, age and/or ethnicity).

Criteria for eligibility of a study excluded in the qualitative and quantitative analysis will be:

- Reviews and meta-analyses, case reports, editorials, letters, meeting abstracts, personal opinions or commentaries, books chapters and non-English language articles.

- No melanoma (Differents skin cancers will be excluded).

- Experimental analysis conducted in vitro or in animal models.

- To asses another *CCND1* alterations differents to gene amplification (e.g. polymorphisms).

- Lack of the precedent essential survival endpoints or clinicopathological features.

- Insufficient statistical data.

Primary outcome(s).

- Overall survival

- Disease free survival

Secondary outcome(s).

- Type/subtype of melanoma

- Breslow thickness

- Ulceration

- N status

- M status

- Clinical stage

- Clark level

- Growth phases

- Regression

- Satellitosis

- Mitotic index

Data extraction (selection and coding).

- First author

- Year of publication

- Country and continent where the study was conducted.

- Sample size

- Recruitment period

- Type of melanomas

- Anatomic location

- Follow up period

- Methods

- Cut off value

- Immunostaining pattern (nuclear/cytoplasmic)

- Survival endpoints and/or clinicopathological features.

Risk of bias (quality) assessment.

The risk of bias in individual studies will be assessed using the Quality in Prognostic Studies (QUIPS) tool, developped by Cochrane prognosis methods group. Specifically, it contains 6 domains: study participation, study attrition, prognostic factor measurement, outcome measurement, study confounding and statistical analysis and reporting. Each domain will be rated as low, moderate or high risk of bias for each study.

Strategy for data synthesis.

Differences in the alterations of CCND1/cyclin D1 will be categorized as positive/high and negative/low, according to the cut-off value chosen by the authors. Odds ratios (OR) and 95% confidence intervals (CI) will be used as the measure of association to determine the correlations between CCND1/cyclin D1 alterations and clinicopathological features. Hazard ratios (HR) and 95%CI will be used as the measure of association to estimate the impact of CCND1/cyclin D1 alterations on time-to-event parameters (overall survival and disease-free survival). In meta-analyses, OR and HR will be pooled when appropriate, using both fixed-effect and/or random-effects models. Forest plots will be used to examine the overall effect. Heterogeneity between studies will be checked using the χ^2 based Cochran's Q test ($p<0.10$). We will quantify the proportion of heterogeneity between studies with the I^2 statistic. In addition, sensitivity analyses will be performed to explore the influence of each individual study on the estimation of the overall effect and to test the reliability of the overall pooled results. Funnel plots will be constructed when appropriate, to assess small-study effects such as publication bias. Egger's test ($p<0.10$) will also be used to statistically assess funnel plots asymmetry. All statistical analyses will be performed with Stata version 14 (Stata Corp, College Station, TX, USA) using user-written commands. In meta-analyses, a two-tailed p-value <0.05 will be considered statistically significant.

Analysis of subgroups or subsets.

We will perform different subgroup analyses (by continent, type/subtype of melanoma, cut off value and immunostaining pattern) to asses potential confounding factors as a possible heterogeneity source, and to explore the relations between *CCND1*/cyclin D1 alterations and the precedent outcomes in these subgroups.
